# Correlations between the Electronic Structure and Energetics of the Catalytic Steps in Homogeneous Water Oxidation Catalysis

**DOI:** 10.1021/jacs.3c05741

**Published:** 2023-10-10

**Authors:** Daan den Boer, Dennis G. H. Hetterscheid

**Affiliations:** Leiden Institute of Chemistry, Leiden University, 2300RA, Leiden, The Netherlands

## Abstract

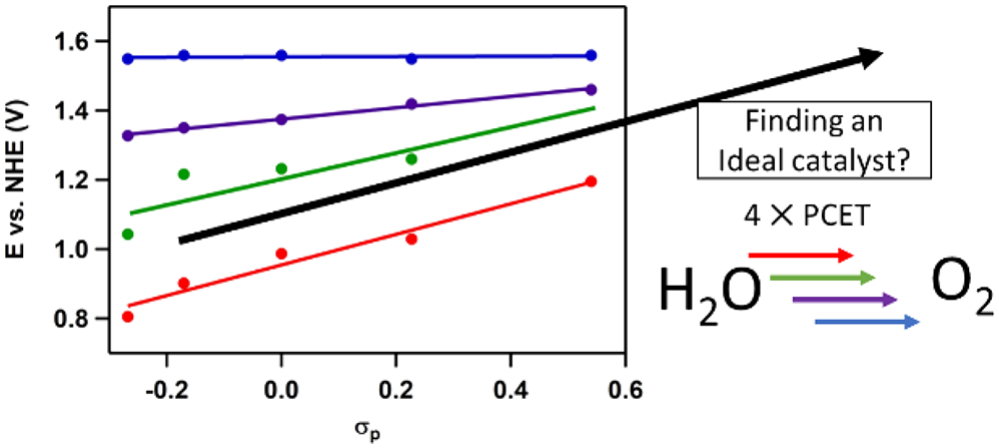

The development of an efficient electrocatalyst for the water oxidation reaction is limited by unfavorable scaling relations between catalytic intermediates, resulting in an overpotential. In contrast to heterogeneous catalysts, the electronic structure of homogeneous catalysts can be modified to a great extent due to a tailored ligand design. However, studies utilizing the tunability of organic ligands have rarely been conducted in a systematic manner and, as of yet, have not produced catalytic paths that avoid the aforementioned unfavorable scaling relations. To investigate the influence of electron-donating groups (EDGs) or electron-withdrawing groups (EWGs) on elementary steps in electrochemical water oxidation catalysis, *cis*-[Ru(bpy)_2_(H_2_O)]^2+^ (bpy = 2,2′-bipyridine) was selected as the scaffold that was modified with methyl, methoxy, chloro, and trifluoromethyl groups. This catalyst can undergo several electron transfer (ET), proton transfer (PT), and proton-coupled electron transfer (PCET) steps that were all probed experimentally. In this systematic study, it was found that PCET steps are relatively insensitive with respect to the presence of EDGs or EWGs, while the decoupled ET and PT steps are more heavily affected. However, the influence of the substituents decreases with an increasing oxidation state of Ru due to a lack of d-electrons available at the Ru center for π-backbonding to the bipyridine ligand. Therefore, the Ru^V/VI^ redox couple appears to be relatively unaffected by the substituent. Nevertheless, the implementation of EWGs can shift all oxidation events to a very narrow potential window. Not only do our findings illustrate how electronic substituents affect the entire potential energy landscape of the catalytic water oxidation reaction, but they also show that the *cis*-[Ru(bpy)_2_(H_2_O)]^2+^ compounds follow different design rules and scaling relations, as has been reported for every other oxygen evolution catalyst thus far.

## Introduction

In view of the worldwide increasing energy demand, a highly sustainable alternative energy source needs to be utilized, as the major share of this energy demand is currently extracted from fossil fuels.^1−5^ As a consequence, the use of fossil fuels has led to global warming and environmental pollution. A sustainable alternative can be provided by electricity obtained from, for example, solar and wind energy, while using dihydrogen as an energy carrier via water electrolysis. The design of efficient electrocatalysts to split water efficiently remains a challenge due to the complexity of the water oxidation (WO) reaction, which consists of removal of four electrons and four protons from two water molecules and additionally the formation of an O–O bond.^6−8^ In the past decade, many heterogeneous and homogeneous WO electrocatalysts have been reported.^9−14^ Nevertheless, an energy-efficient electrocatalyst that can operate at a low overpotential and with fast kinetics has not been found yet.

**Figure 1:**
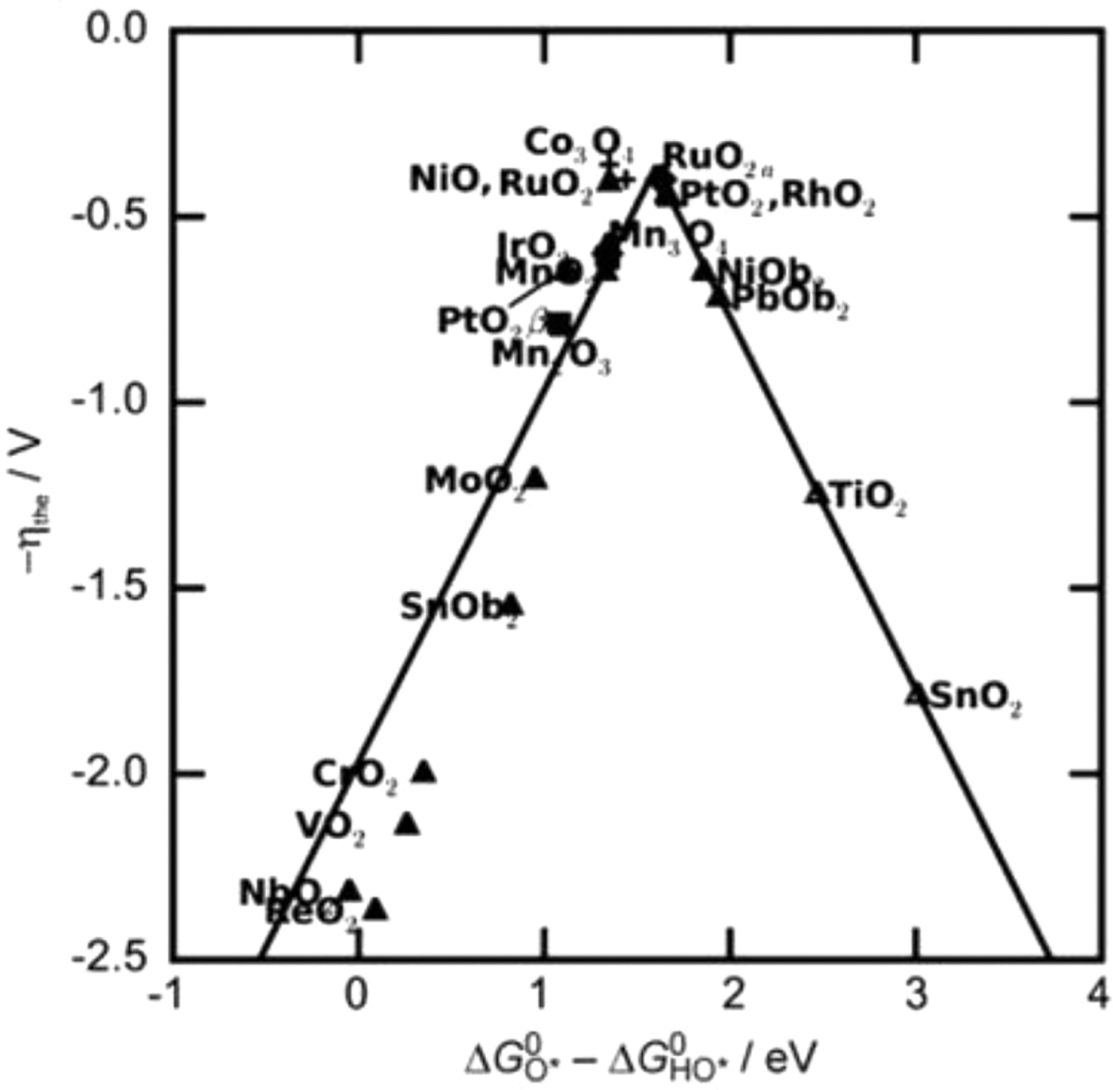
Volcano plot of the oxygen evolution reaction wherein the theoretical overpotential (η) is plotted as a descriptor that resembles the electronic structure of the catalyst. Reproduced from ref (23) with permission from Wiley.

For an ideal electrocatalyst, the Sabatier principle—“*a substrate should bind neither too strong nor too weak”*—should be met for all catalytic intermediates.^15^ For such an ideal catalyst, the thermodynamic barriers to access all relevant catalytic intermediates should be zero, resulting in a catalyst that does not require an additional driving force (overpotential) beyond the equilibrium potential of water to drive the forward reaction. In practice, balancing all catalytic intermediates one-by-one to the most ideal binding energy is very difficult, and typically, an additional potential must be applied to access all catalytic intermediates and to initiate the forward reaction. For two-electron reactions, such as the hydrogen evolution reaction, an ideal catalyst can be obtained due to the limited number of catalytic intermediates, which therefore are more easily aligned. However, multielectron reactions such as WO are far more challenging given that a significantly larger number of intermediates has to be aligned on the potential energy landscape of the catalytic cycle. Systematic studies in the field of heterogeneous catalysis have shown that it is impossible to adjust the binding energy of a single intermediate without affecting the binding energy of other intermediate species for multielectron reactions such as WO.^16−19^ In fact, some of the key binding energies scale linearly, which makes finding an ideal catalyst theoretically impossible. Therefore, the best WO catalysts are restricted to an overpotential of roughly 320 mV (1 M NaOH, 10 mA/cm^2^),^20−22^ while in terms of the Sabatier principle a best compromise is found for these catalytic systems, illustrated by the volcano plot in Figure 1.

Homogeneous catalysis provides a significant number of additional tools for catalyst design that are unavailable in the field of heterogeneous catalysis.^10,11^ The broad choice of ligands in combination with transition metals provides an enormous number of options. Herein, the number and type of ligands, the ligand hapticity, and ligand charge offer tools to fine-tune the electronic and geometric structure of homogeneous catalysts.^24^ In the past three decades, the focus was mostly on the design of stable, robust, and fast homogeneous electrocatalysts for WO under oxidative conditions.^8,25−31^ However, fine-tuning of the thermodynamics of elementary steps, in order to decrease the overpotential, has been investigated in less detail. The electronic structure of the catalysts and the elementary steps outlined above can be influenced by electronic effects induced by substituent groups that have an electron-donating or electron-withdrawing character. However, systematic studies of these electronic effects on the entire potential energy landscape of WOCs have rarely been documented. Yet such studies may unravel clear design principles similar to scaling relations in heterogeneous catalysis.

Correlations between structure and activity have been reported in some cases for ruthenium-,^32−36^ cobalt-,^37^ and copper^38−41^-based water oxidation catalysts. Yet, these structure–activity correlation studies have always been restricted to a single descriptor of the catalytic reaction, such as a turnover frequency or the onset potential for catalysis. The reported studies do show that the overpotential and catalytic activity for WO can be optimized by introducing electron-withdrawing group (EWG) and electron-donating group (EDG) substituents on the ligand. Yet, it has not been addressed how these modifications affect the many other elementary steps within the catalytic cycle and to which extent such modifications lead to unfavorable scaling of elementary steps. Nevertheless, high-throughput computation screening of large libraries of molecular water oxidation catalysts do show that homogeneous catalysts are limited by the same scaling relations as heterogeneous species, when these oxidize water via the same sequence of elementary steps.^42^

In the research described in this article, we set out to systematically study the effect of EDGs and EWGs on all elementary steps within a WO mechanism. As a scaffold we selected the compound *cis*-[Ru(bpy)_2_(H_2_O)]^2+^, which was already studied in detail by Dobson and Meyer in 1988.^43^ The advantage of the *cis*-[Ru(bpy)_2_(H_2_O)]^2+^ system is that all four redox steps that are necessary to oxidize water can be experimentally probed by cyclic voltammetry in a broad pH window. Moreover, the main selling point of employing the *cis*-[Ru(bpy)_2_(H_2_O)]^2+^ system is that it operates via a different sequence of elementary steps compared to all previous studies and, thus, may not be restricted by the same limiting scaling relations.^42^ Oxidation of the Ru complexes from the +II to the +VI oxidation state proceeds via a multitude of electron-transfer (ET), proton-transfer (PT), and proton-coupled electron-transfer (PCET) steps. We have investigated the effect of EDGs and EWGs on the thermodynamics and kinetics of all relevant elementary steps in the electrocatalytic WO reaction and as a function of pH. We now for the first time show the effect of electronic structure modifications on all local minima of the entire potential energy landscape of a homogeneous water oxidation catalyst and thereby provide a clear picture of how catalyst optimizations effect the entire potential energy surface. Moreover the correlations between the electronic structure and the energetics of all individual elementary steps illustrate future directions toward the development of an ideal water oxidation catalyst from a redox potential leveling point of view.

## Results

### Synthesis and Characterization

The effects of EDGs and EWGs on elementary ET, PT, and PCET steps in the WO mechanism of *cis*-[Ru^II^(4,4′-R_2_-bpy)_2_(H_2_O)_2_](OTf)_2_ were investigated by using complexes in which the 4- and 4′-positions (= R) of the 2,2′-bipyridine were modified with Me, OMe, Cl, and CF_3_ groups. One of the substituents on the 4- or 4′-positions will be positioned *trans* to the coordinated aqua ligands in the octahedral geometry, allowing for electron effects through conjugation. The substituents were selected on the basis of their Hammett parameters (σ_p_), which describe the electron-donating or -withdrawing effect.^44−46^ The selected substituents cover a broad range in terms of their Hammett parameters and do not contain acidic or basic functionalities, which could complicate the interpretation of the results.

**Scheme 1:**
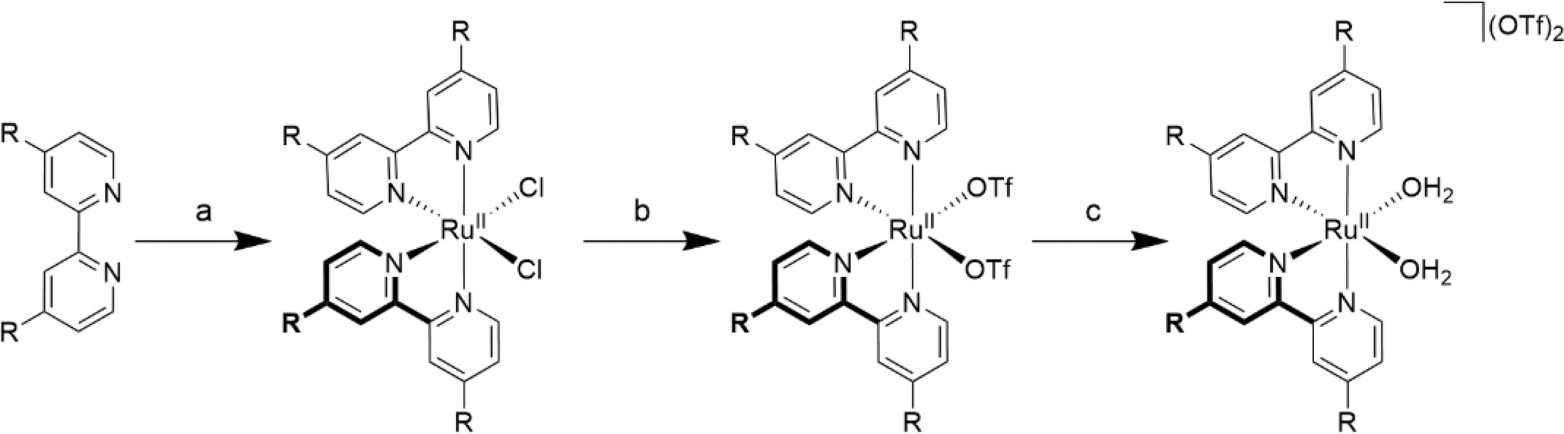
Synthesis of *cis*-[Ru(4,4′-R_2_-bpy)_2_(X)]^2+^ Derivatives (R = Me, OMe, H, Cl, or CF_3_ and X = Cl, OTf, or H_2_O) Reagents and conditions: (a) [Ru(cod)Cl_2_], 1,2-dichlorobenzene, reflux, 2.5 h; (b) triflic acid, 1,2-dichlorobenzene, room temperature, 1 h; (c) H_2_O.

The *cis*-[Ru^II^(4,4′-R_2_-bpy)_2_(H_2_O)_2_](OTf)_2_ complexes were synthesized in two steps. Complexes of *cis*-[Ru(4,4′- R_2_-bpy)_2_Cl_2_] were prepared by treatment of [Ru^II^(cod)Cl_2_] (cod = 1,5-cyclooctadiene) with 4,4′-R_2_-bpy (Scheme 1). Employing these chloride compounds as electrocatalysts was not attempted, as chloride ions are known to act as cocatalysts for WO by formation of HOCl.^47^ Furthermore, the chloride complexes have a significantly lower water solubility in comparison to their triflate analogues. Therefore, the chloride ligands are exchanged for triflates in a reaction with HOTf in dichlorobenzene to obtain *cis*-[Ru^II^(4,4′-R_2_-bpy)_2_(OTf)_2_]. Upon dissolution of these compounds in water, the triflate ions are displaced immediately by water molecules, resulting in the formation of *cis*-[Ru^II^(4,4′-R_2_-bpy)_2_(H_2_O)_2_](OTf)_2_.

A clear change in color was observed for the solutions containing *cis*-[Ru^II^(4,4′-R_2_-bpy)_2_(H_2_O)_2_]^2+^, varying from red/pink to light green for CF_3_ > Cl > H > Me > OMe. UV–vis spectra of the *cis*-[Ru^II^(4,4′-R_2_-bpy)_2_(H_2_O)_2_]^2+^ compounds were recorded in Milli-Q water (Figure S1). The area of 200 to 300 nm contains π–π* transitions of the bpy ligands.^48^ Furthermore, two metal-to-ligand charge-transfer (MLCT) bands are found in the ranges 330 to 355 and 475 to 505 nm. Finally, weak broad transitions are found in the range of 550 to 700 nm. The sharp π–π* and two MLCT bands were found to shift linearly as a function of the Hammett parameter as expected (Figure S1).

### Cyclic Voltammetry

**Figure 2:**
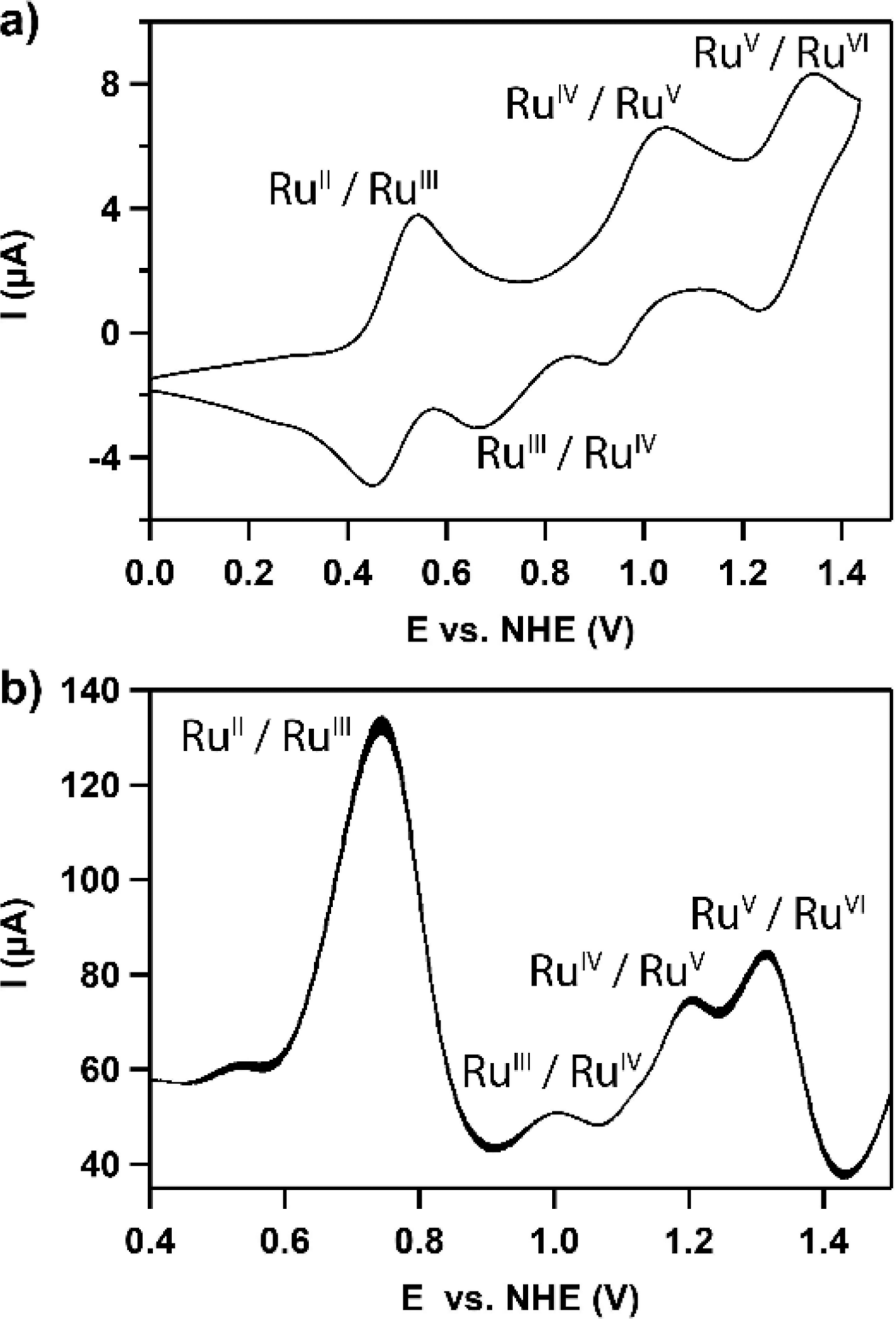
(a) CV of 0.5 mM *cis*-[Ru^II^(bpy)_2_(H_2_O)_2_]^2+^ in 100 mM pH 7 phosphate buffer at a scan rate of 100 mV/s. Boron-doped diamond (BDD), Au, and reversible hydrogen electrode (RHE) were used as the working electrode (WE), counter electrode (CE), and reference electrode (RE), respectively. Potentials were converted to the normal hydrogen electrode (NHE). (b) Example of differential pulse voltammogram of 1.0 mM *cis*-[Ru(bpy)_2_(H_2_O)]^2+^ in 100 mM pH 4 phosphate buffer, including four oxidative waves of which the one at 1.0 V vs NHE highlights the Ru^III/IV^ redox couple. Glassy carbon (GC), Au, and RHE were used as WE, CE, and RE, respectively. Potentials were converted to NHE.

The group of Meyer studied *cis*-[Ru^II^(bpy)_2_(H_2_O)_2_]^2+^ with cyclic voltammetry in an aqueous pH 7 phosphate buffer.^43^ We successfully reproduced these electrochemical experiments. The cyclic voltammogram (CV) of *cis*-[Ru^II^(bpy)_2_(H_2_O)_2_]^2+^ shows three oxidation and four reduction events in the range of 0.4 to 1.4 V vs NHE (Figure 2a). Three reversible waves assigned to the Ru^II/III^, Ru^IV/V^, and Ru^V/VI^ redox couples are found at *E*_1/2_ 0.50, 0.98, and 1.29 V vs NHE, respectively. For the Ru^III/IV^ redox couple, only a reductive wave is observed. Meyer et al. explained the absence of the oxidative wave by the slowness of the oxidation process, which mechanistically proceeds via disproportionation of two Ru^III^ species with the formation of Ru^II^ and Ru^IV^. However, Meyer et al. were able to detect the oxidative redox wave by utilization of “activated” GC electrodes.^43,49^ We applied differential pulse voltammetry (DPV) to visualize the oxidation of Ru^III^ to Ru^IV^ (see Figure 2b for an example). The Ru^III/IV^ oxidation was significantly more prominent in DPV plots obtained from acidic media than those from neutral or basic media (Figures S25–S29).

**Figure 3:**
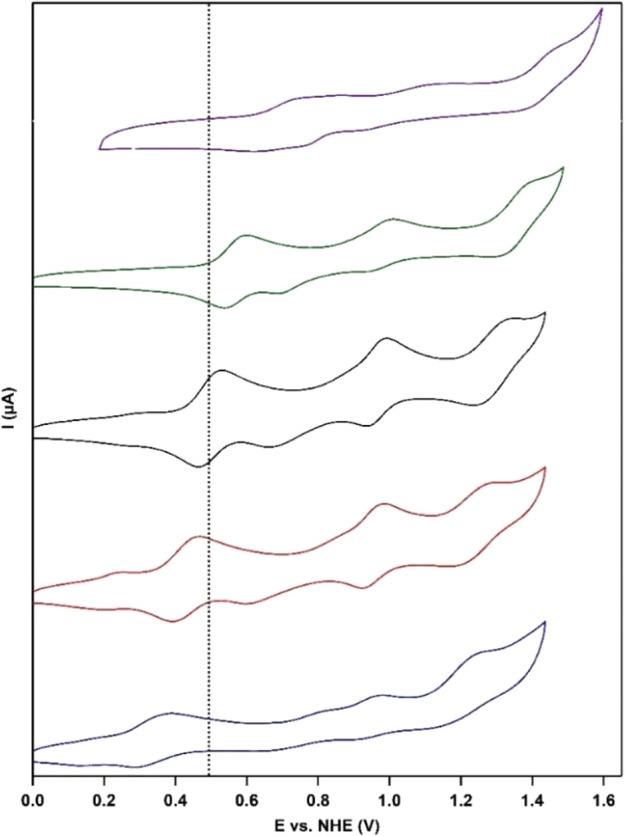
CVs of 0.5 mM *cis*-[Ru(4,4′-R_2_-bpy)_2_(H_2_O)_2_]^2+^ recorded in 100 mM pH 7 phosphate buffer at a scan rate of 100 mV/s. From top to bottom, R = CF_3_ (purple), Cl (green), H (black), Me (red), and OMe (blue). BDD, Au, and RHE were used as WE, CE, and RE, respectively. Potentials were converted to NHE. The dotted black line corresponds to *E*_1/2_(Ru^II^/Ru^III^) of the benchmark *cis*-[Ru^II^(bpy)_2_(H_2_O)_2_]^2+^.

CVs were also recorded for the *cis*-[Ru(4,4′-R_2_-bpy)_2_(H_2_O)_2_]^2+^ (R = Me, OMe, Cl, and CF_3_) derivatives in a pH 7 phosphate buffer (Figure 3). Again three oxidation and four reduction events were found, as was shown for the benchmark system (R = H). As expected, the redox couples shift as a function of the electron-donating or -withdrawing effect of the substituent.

### Pourbaix Diagrams

**Scheme 2:**
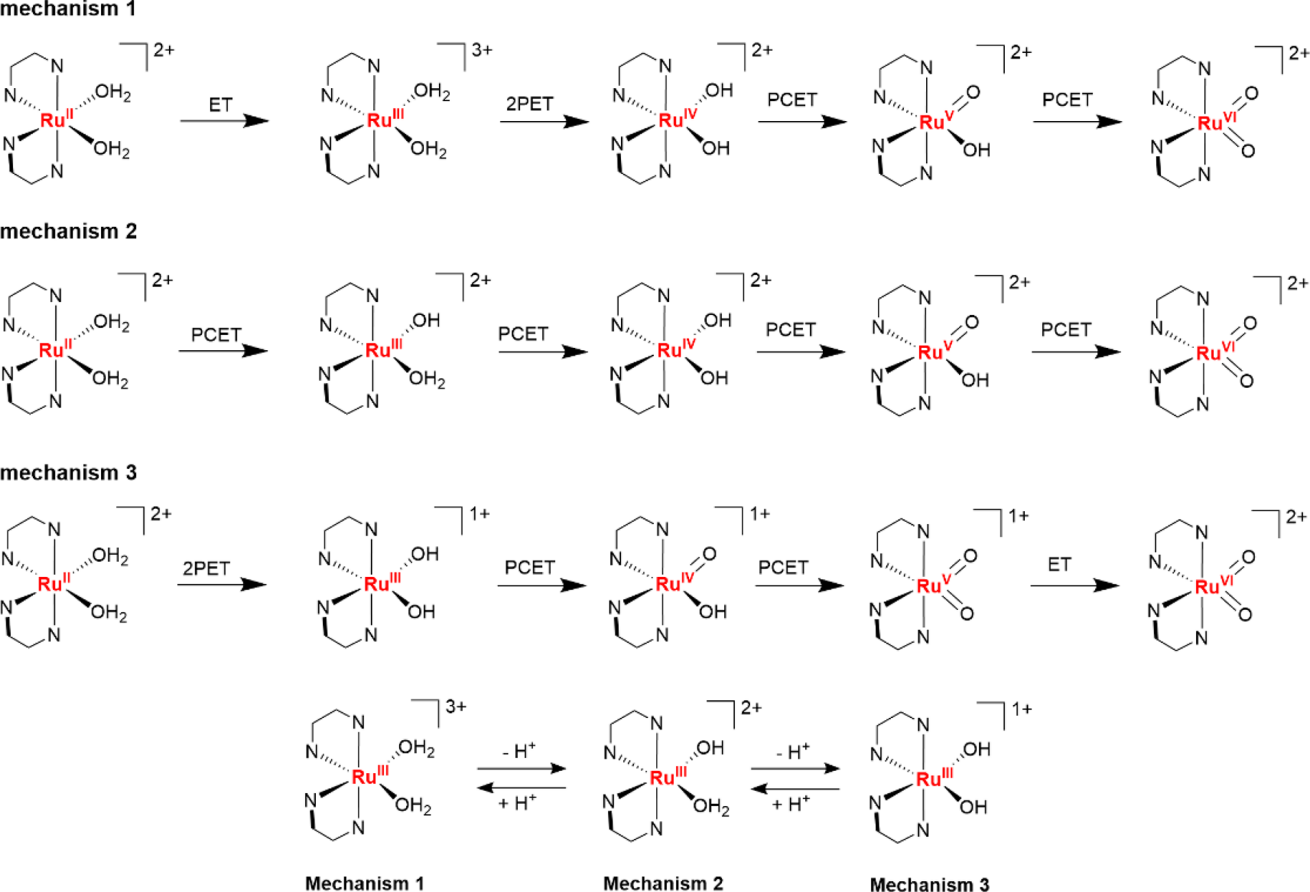
Three Mechanistic Pathways Leading to the Formation of Ru^VI^(=O)_2_ and Their Dependence on the pH Illustrated for the Ru^III^ Intermediate

**Figure 4:**
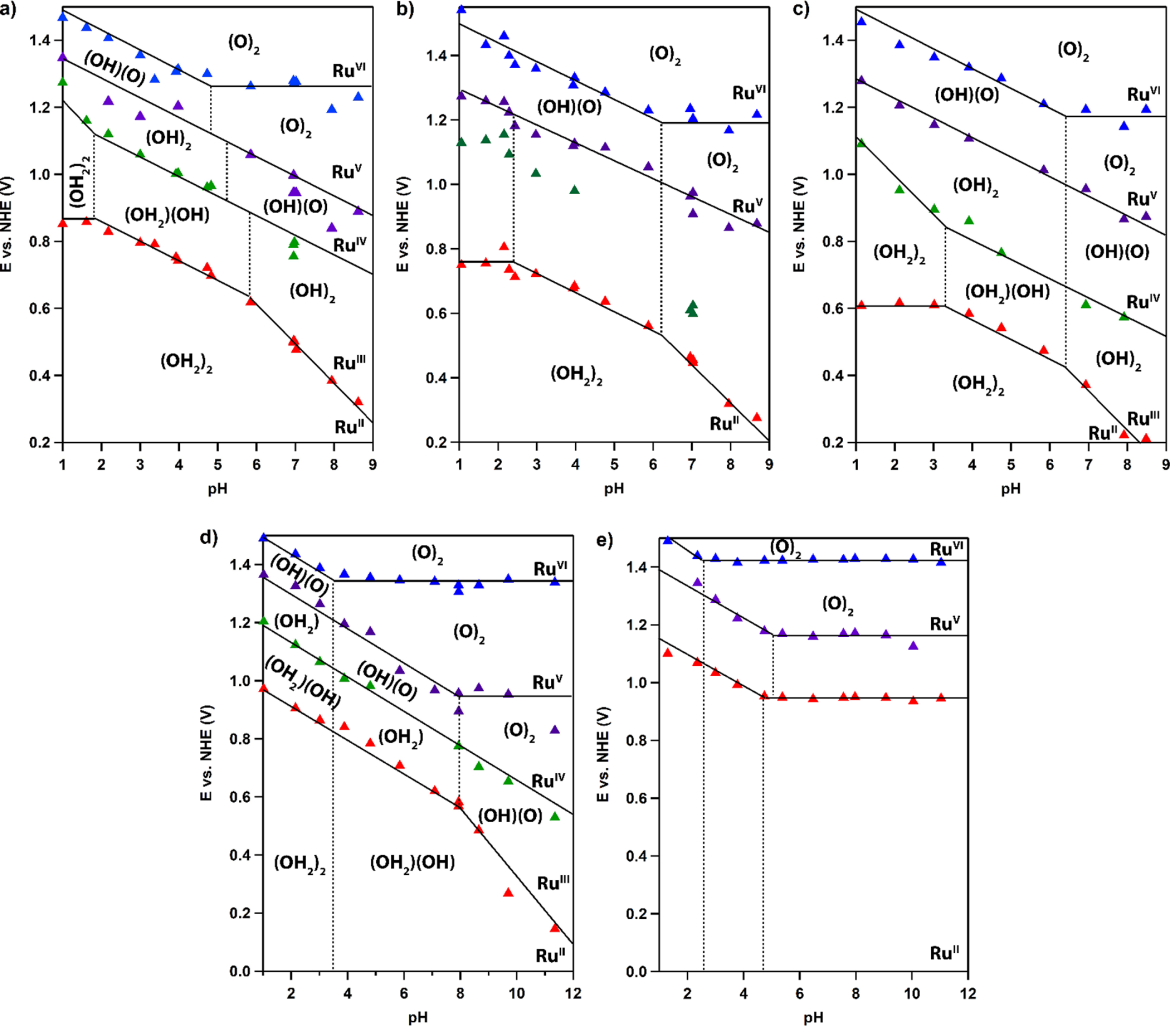
Pourbaix diagrams of *cis*-[Ru^II^(4,4′-R_2_-bpy)_2_(H_2_O)_2_]^2+^ with R = H (a), Me (b), OMe (c), Cl (d), and CF_3_ (e). Red, green, purple, and blue correspond to redox potentials obtained from CV and DPV experiments for Ru^II/III^ to Ru^V/VI^, respectively.

Cyclic voltammetry and DPV experiments were recorded for *cis*-[Ru^II^(bpy)_2_(H_2_O)_2_]^2+^ in aqueous solutions as a function of pH to construct a Pourbaix diagram (Figure 4a). Our findings are in excellent agreement with the Pourbaix diagram previously reported by the Meyer group (Figure S2).^43^ Three pH regions are found, in which the active species for WO, *cis*-[Ru^VI^(bpy)_2_(=O)_2_]^2+^, is generated via different pathways. These pathways are discussed one by one. The first mechanism appears in the acidic region below pH 2 (Scheme 2, mechanism 1). Via an electron-transfer, a two-protons-one-electron-transfer (2PET), and two proton-coupled electron-transfer steps, the active species is obtained. In the range of pH 2–5, *cis*-[Ru^VI^(bpy)_2_(=O)_2_]^2+^ is obtained via four PCET steps (Scheme 2, mechanism 2). Above pH 5 the formation of *cis*-[Ru^VI^(bpy)_2_(=O)_2_]^2+^ proceeds via sequential 2PET, PCET, PCET, and ET steps (Scheme 2, mechanism 3). In this case, a Ru(=O)_2_ species is already formed at the +V oxidation state. However, this species must first undergo another ET step for it to be able to evolve dioxygen, as no catalytic wave was observed directly after the formation of this Ru^V^ species.

Several things must be noted when comparing the three reaction paths by which *cis*-[Ru^VI^(bpy)_2_(=O)_2_]^2+^ is formed. First, in the change of mechanism 2 to mechanism 3, the final oxidation step changes from a PCET to an ET. Consequently, the formation of *cis*-[Ru^VI^(bpy)_2_(=O)_2_]^2+^ and thus the overpotential for WO scale differently with pH above and below pH 4.8. The overpotential for WO remains roughly 320 mV in the potential window of pH 1 to 4.8, where it scales with the NHE reference electrode. In contrast, the overpotential rapidly increases with 59 mV per pH unit in the potential window of pH 4.8 to 9. Finally, the shift in mechanism between mechanisms 1, 2, and 3 is linked by the p*K*_a_ values of the Ru^III^ (Scheme 2), Ru^IV^, and Ru^V^ aqua or hydroxo species.

Pourbaix diagrams were also constructed for the other *cis*-[Ru^II^(4,4′-R_2_-bpy)_2_(H_2_O)_2_]^2+^ derivatives (Figure 4b–e). The pH region of pH 1 to 9 was investigated for R = Me and OMe, as it has been done for R = H. However, for R = Cl and CF_3_ the pH range was extended to pH 12, as above pH 9 new features were observed in the Pourbaix diagram. As a result of the electronic effects induced by EDGs and EWGs, the pathways described above occur in different pH ranges for R = Me, OMe, Cl, and CF_3_. For the EDGs mechanism 1 occurs in a broader pH range, namely, in the range of pH 1–2.4 for Me and pH 1–3.0 for OMe. For the EWGs Cl and CF_3_, mechanism 1 was not observed in the selected pH window. In contrast, mechanisms 2 and 3 do appear in the Pourbaix diagrams of all catalysts. The pH window in which mechanism 2 occurs has shifted to another pH range for Me and OMe, namely, from pH 1.8–4.8 to 2.4–6.2 for Me and 3.3–6.4 for OMe. In the case of the EWGs Cl and CF_3_, mechanism 2 occurs below pH 3.5 and 2.4, respectively. Also, in the case of the Cl complex, the Ru^II^ species is already deprotonated in the pH window in which mechanism 3 occurs. As a result, the first oxidation step proceeds via PCET instead of a 2PET step.

**Scheme 3:**
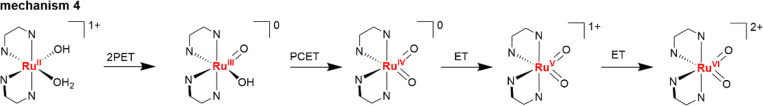
Mechanistic Pathway Leading to the of the Formation of Ru^VI^(=O)_2_ Occurring at Alkaline Conditions and with Strong EWGs

Furthermore, in the pH window of 8–12 a fourth mechanism is found for the catalyst with Cl substituents. Starting from the deprotonated Ru^II^ oxidation state, 2PET, PCET, and two ET steps occur sequentially to form *cis*-[Ru^VI^(bpy)_2_(=O)_2_]^2+^ (Scheme 3, mechanism 4). In this mechanism, a Ru(=O)_2_ species is formed already in the +IV oxidation state, with the +III and +IV ruthenium complexes having a neutral overall charge.

For all Ru(=O)_2_ species the catalytic wave starts after the formation of the +VI oxidation state in acidic and neutral pH. However, in the case of R = Cl and at high pH (=11.4), a catalytic wave was observed after the formation of the +V species (Figure S5). Thus, when functionalized with a strong EWG, Ru(V) can also catalyze WO. Nevertheless, the oxidative wave assigned to the Ru^V/VI^ transition can still be observed in the catalytic wave. It must be noted that an overpotential of at least +800 mV is required for WO by formation of Ru^VI^ under these conditions.

For the more electron-withdrawing CF_3_ group, the redox couples shift closely toward each other, especially in the acidic range of pH 1–2.4 (Figure 4e). As a result, detection of the Ru^III/IV^ redox event becomes impossible, as it lies in very close proximity to the Ru^II/III^ redox couple, and a merged oxidative and reductive wave is observed. Therefore, we have refrained from assigning all of the intermediates in the Pourbaix diagram of *cis*-[Ru^II^(4,4′-(CF_3_)_2_-bpy)_2_(H_2_O)_2_]^2+^. However, in the acidic pH range of pH 1–2.4, all four redox couples occur within a range of 360 mV.

Based on the constructed Pourbaix diagrams, it becomes clear that changes in the electronic structure have a strong effect on the electrochemical behavior of the catalyst, with the direct consequence that the pH window in which a particular mechanism is operational shifts considerably. In the case of the EWGs that were employed, even a new mechanistic pathway was found. These results illustrate that modified catalysts should not be compared in structure–activity correlation studies as long as their mechanistic pathway is not verified, as the mechanism to which water is oxidized might differ significantly from the catalyst to which it is compared to.

### Hammett Correlations

Comparing the Pourbaix diagrams of the various complexes allows us to directly correlate the Hammett parameter (σ_p_) of the substituents with the thermodynamic values for the ET, PT, and PCET events. The value of σ_p_ describes the electronic effect by the substituent group and is negative for EDGs and positive for EWGs relative to H (σ_p_ = 0).^44−46^ The *E*_1/2_ redox potentials of the redox couples were extracted from the Pourbaix diagrams by extrapolation of the slopes in the Pourbaix diagram to pH 0. These *E*_1/2_ redox potentials were determined for redox transitions observed in all Pourbaix diagrams (Scheme 2, Figure 4). The p*K*_a_ values were estimated from the Pourbaix diagram at points where functions change from ET to PCET and vice versa. The slope in the Hammett plots equals rho (ρ), which describes the correlation between the redox potential or p*K*_a_ of the Ru system with σ_p_ and thereby the electronic effect of the EDGs or EWGs. The magnitude of the σ_p_ effect can be compared for different ET or PCET and PT steps by comparing the ρ values. If ρ increases, then the dependence of *E*_1/2_ of the redox couple or p*K*_a_ on σ_p_ also increases. The influence of σ_p_ on the p*K*_a_ values and redox potentials of the Ru intermediates is discussed step-by-step.

Two ρ values of −5.4 and −2.2 can be identified for the p*K*_a_ of the first and second deprotonation of Ru^III^(OH_2_)_2_ (Scheme 2 and Figure S6). Thus, for both deprotonations, the p*K*_a_ increases as a function of the electron-donating character of the substituent, as expected and in agreement with negative ρ values. The difference in the values of ρ indicates that the first deprotonation is more sensitive to the electron-donating character of the substituent than the second deprotonation (Scheme 2). For the Cl and CF_3_ substituents, the p*K*_a_ of the first deprotonation could not be determined experimentally. Extrapolating the correlation between ρ and the Hammett parameter for the first deprotonation suggests that the p*K*_a_ of *cis*-[Ru^II^(4,4′-Cl_2_-bpy)_2_(H_2_O)_2_]^2+^ must lie below 1, which is in agreement with the absence of mechanism 1 in the Pourbaix diagram of R = Cl and CF_3_.

Depending on the pH, oxidation of Ru^II^ to Ru^III^ may occur via ET, PCET, or 2PET steps. The transition of the species Ru^II^(OH_2_)_2_ to Ru^III^(OH_2_)_2_ proceeds via an ET step with a ρ value of +0.95 (Figure S6). The transition of Ru^II^(OH_2_)_2_ to Ru^III^(OH_2_)(OH) via a PCET step proceeds with a ρ value of +0.44 (Figure S6), while the transition of Ru^II^(OH_2_)_2_ to Ru^III^(OH)_2_ proceeds via a 2PET step with a ρ value of +0.53 (Figure S6). Thus, the sensitivity to σ_p_ is the highest for the ET process, while the ρ values for the PCET and 2PET steps are of the same order. This indicates that the simultaneous subtraction of protons during electron-transfer processes decreases the sensitivity to the σ_p_ of the substituent.

It is challenging to find a sufficiently accurate equilibrium potential for the Ru^III/IV^ redox couple for all substituted compounds due to the disproportionation process associated with this oxidation reaction. Despite the error in the *E*_1/2_, it is clear from our data that the Ru^III^/Ru^IV^ couple in all cases is located between the Ru^II^/Ru^III^ and the Ru^IV^/Ru^V^ redox couples and shifts along accordingly.

Oxidation of Ru^IV^ to Ru^V^ was found to proceed via PCET throughout the entire pH window for R = H, Me, and OMe. For this PCET step, a ρ value of +0.16 was found, which is smaller than the ρ values found for the Ru^II/III^ redox couple (Figure S6), indicating that the electronic properties of the substituents do not have a large influence on this process.

Formation of Ru^VI^(=O)_2_ proceeds either via a PCET or via an ET step. It is the p*K*_a_ of the Ru^V^(=O)(OH) intermediate that underlies the change between the PCET and ET steps. The p*K*_a_, extracted from the Pourbaix diagrams, depends on the Hammett parameter with a ρ of −5.3 (Figure S6). The effect of σ_p_ on p*K*_a_ is on the same order for Ru^III^(OH_2_)_2_ and Ru^V^(=O)(OH) with values of −5.4 and −5.3, respectively.

**Figure 5:**
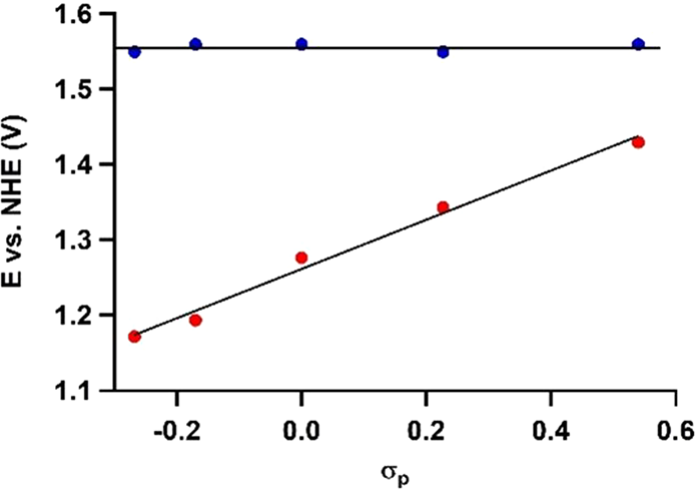
Potential of the Ru^V^/Ru^VI^ redox couple proceeding via PCET (blue) according to mechanisms 1 and 2 (i.e., oxidation of *cis*-[Ru^V^(4,4′-R_2_-bpy)_2_(=O)(OH)]^2+^ to *cis*-[Ru^VI^(4,4′-R_2_-bpy)_2_(=O)_2_]^2+^) and via an ET step (red) in mechanism 3 (i.e., via oxidation of *cis*-[Ru^V^(4,4′-R_2_-bpy)_2_(=O)(=O)]^+^ to produce *cis*-[Ru^II^(4,4′-R_2_-bpy)_2_(=O)_2_]^2+^) versus the Hammett parameter. The formation of Ru^VI^ via PCET and ET reactions occurs with ρ values of 0 and +0.33, respectively, and occurs in different pH windows.

Formation of the Ru^VI^(=O)_2_ species is critical for the success of the WO reaction, as it is the nucleophilic attack of water on this species that leads to formation of the O–O bond. Our data show that the potential at which formation of Ru^VI^(=O)_2_ forms via PCET from Ru^V^(=O)(OH) does not depend on σ_p_ (Figures 5 and S4).

The oxidation potential of the oxidation of Ru^V^(=O)_2_ to Ru^VI^(=O)_2_ proceeding via an ET step was found to depend on the Hammett parameter with a ρ value of +0.33, indicating that the ET is dependent on σ_p_ of the substituent. This is in contrast to the σ_p_-independent PCET transition. However, this sensitivity is significantly lower for the Ru^V/VI^ than for the Ru^II/III^ redox couple.

**Scheme 4:**
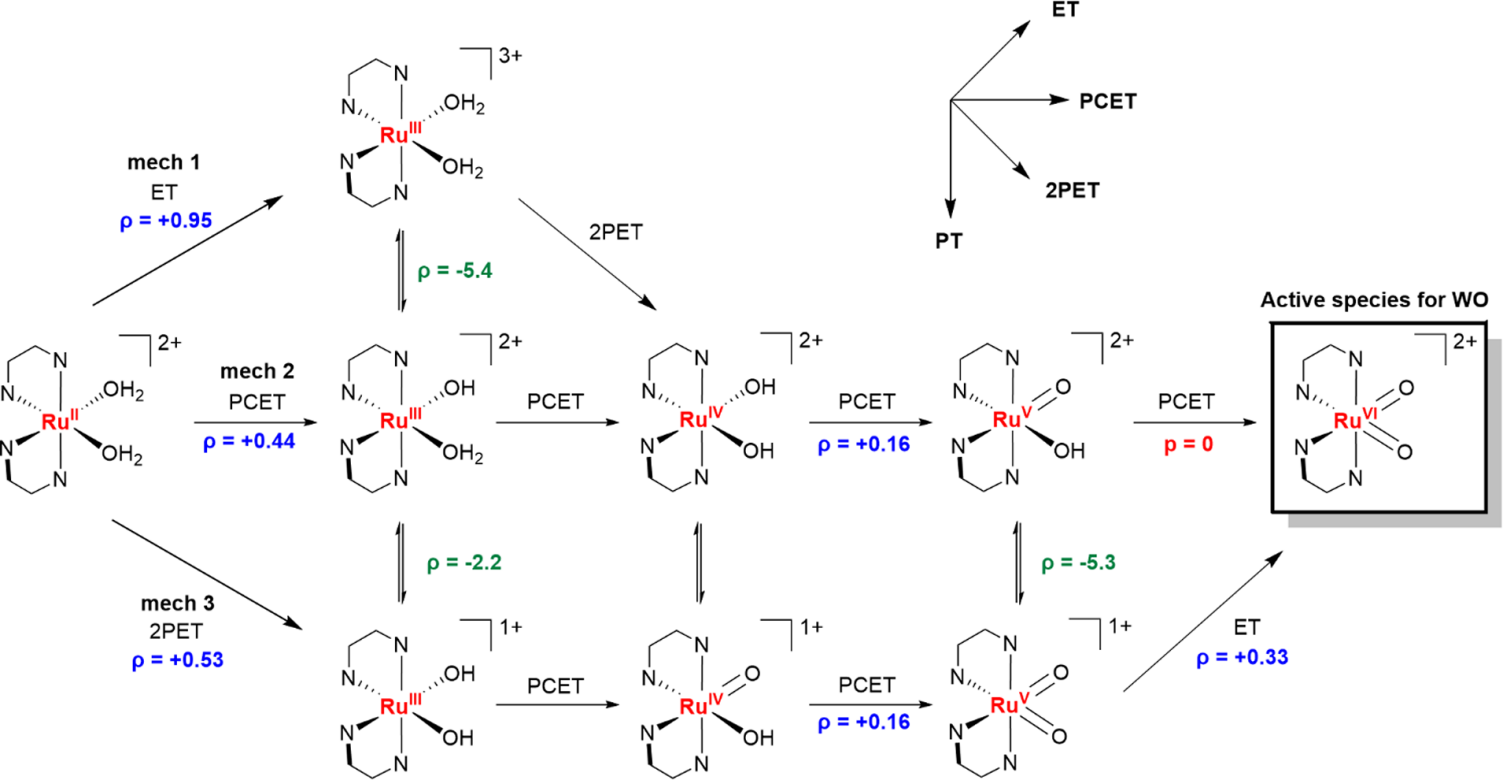
Overview of ρ Values for the p*K*_a_ and Redox Potentials of All Intermediates Relevant to the Water Oxidation Reaction

Overall, the potentials at which ET steps take place are significantly more influenced by EDGs and EWGs than by the PCET steps (Scheme 4). Moreover, the sensitivity to σ_p_ decreases with increasing oxidation states. This decrease is illustrated in mechanism 2, where all steps proceed via PCET and the ρ decreases gradually from +0.44 to zero (Scheme 4).

### Kinetic Analysis

The mechanism of O–O bond formation by *cis*-[Ru^II^(bpy)_2_(H_2_O)_2_]^2+^ was investigated previously by the group of Llobet, who studied WO in the presence of sacrificial Ce^IV^ salts.^50^ In this study, two mechanistic pathways of the formation of an O–O bond were considered, consisting of WNA or intramolecular bond formation. Based upon their ^18^O-isotopic labeling experiments in combination with DFT and CASSCF/CASPT2 calculations, the O–O bond formation was shown to proceed via a water nucleophilic attack (WNA) on a Ru^VI^=O intermediate. In contrast, Pushkar et al. suggested the O–O bond formation to proceed via the “blue dimer”, based on DFT studies and detection of the dimeric intermediate [{(bpy)_2_Ru^V^(OH)}^51^_2_(μ_2_-O_2_)]^2+^ with EPR spectroscopy and on the basis of kinetic studies with sacrificial reagents.^52^ Our electrochemical data, in terms of both redox couples and catalytic activity, remain unchanged over many cycles in the cyclic voltammogram (Figure S12). This strongly suggests that water oxidation catalysis occurs via the mononuclear mechanism postulated by Llobet et al.

From the Pourbaix diagrams, it is apparent that the catalytic species Ru^VI^(=O)_2_ can be formed from the Ru^V^ intermediate via a PCET or an ET process. To compare the catalytic activity of the complexes with varying σ_p_, independent of the pH and varying buffering capacities of the electrolyte solutions, we selected the pH 2.5 phosphate buffer solution for the comparison. At pH 2.5, the Ru^VI^(=O)_2_ species is formed via a PCET reaction for all EWGs and EDGs, except for the compound with CF_3_ substituents. The formation of Ru^VI^(=O)_2_ via PCET was found to proceed at 1.40 V vs NHE for all complexes in this study. This results in a kinetic comparison, where an equal driving force is applied that lies 450 mV above the Ru^V/VI^ redox couple.

Given that no plateau current could be obtained in the CV, we plotted for all species the catalytic current (*i*_cat_) obtained at 1.85 V vs NHE at a 100 mV/s scan rate, divided by cathodic peak current (*i*_p_) of the Ru^II/III^ redox couple to compare the reaction kinetics. Comparing only the catalytic currents was considered to be inappropriate due to differences in diffusion rates of the ruthenium complexes (more information on the correction factors can be found in Supporting Information, Table S1 and Figures S6–S10). In this kinetic study, the catalyst with CF_3_ groups was not taken into account as the redox couples are partly merged, and assignment of the cathodic peak current of the Ru^II/III^ redox couple (*i*_p_) is therefore imprecise. The *i*_p_ and *i*_cat_ currents were corrected for the background currents. The activity at pH 2.5, where Ru^VI^(=O)_2_ is formed via PCET, depends positively on the Hammett parameter (Figure S11). This indicates that the rate of O–O bond formation increases with more EWGs.

## Discussion

### Electronic Structure Considerations

Cramer and Llobet et al. characterized all *cis*-[Ru(bpy)_2_(H_2_O)_2_]^2+^ intermediates occurring in mechanism 1 experimentally with UV–vis, EPR, and XAS spectroscopy and theoretically by DFT and CASSCF/CASPT2 calculations (Scheme 2).^48^ All intermediates were obtained via oxidation with stoichiometric amounts of Ce^IV^ at pH 1, while EPR analysis and DFT studies showed that all compounds have either a singlet or doublet ground state.^48^ For all catalytic intermediates with oxidation states of +II to +VI DFT geometry optimizations in the gas phase were performed based upon the crystal structure of *cis*-[Ru(bpy)_2_(H_2_O)_2_]^2+^.^48^ Bond lengths, coordination angles, and dihedral angles were calculated following the reaction sequence of mechanism 1. The geometry of the + IV oxidation state could potentially be one of two possible tautomers, *cis*-[Ru(bpy)_2_(OH)_2_]^2+^ and *cis*-[Ru(bpy)_2_(O)(H_2_O)]^2+^. The dihydroxy tautomer was found to be more stable with 0.6 kcal/mol compared to the aqua/oxo tautomer. On top of that, EXAFS analysis indicates that Ru=O is not formed in the +IV oxidation and that oxidation to the +V oxidation state must occur for the first Ru=O moiety to form.^48^

Overall, upon going from Ru^II^ to Ru^VI^, the Ru–O bonds become significantly shorter from 2.226 to 1.688 Å, which is in line with oxygen atoms becoming better donors. The decreasing Ru–O bond length with increasing oxidation states were confirmed with XAS analysis.^48^ As the Ru–O bond shortens, the O–Ru–O angle increases due to electronic repulsion of lone pairs of the oxygen atoms, which also results in a decreasing dihedral angle between the two bipyridine planes. The opposite effect is observed for the Ru–N bond lengths, which increase from 2.02–2.07 to 2.214–2.218 Å with increasing oxidation state of Ru from +II to +VI. This might be the result of a loss of d-electrons that are involved in π-backbonding from the ruthenium d-orbitals into the π*-orbitals of bipyridine.^53−56^

When comparing Ru^II^(OH_2_)_2_ and Ru^III^(OH_2_)_2_, Cramer and Llobet et al. found minor changes in bond lengths and little other geometrical changes between the Ru complexes.^48^ However, the largest Hammett dependence with a ρ of +0.95 was found for the Ru^II/III^ oxidation, indicating a strong influence by the induced electronic effect of the substituent. For the Ru^III/IV^ redox couple, which proceeds via 2PET, the bond lengths are different by almost 0.3 Å for Ru–O and 0.1 Å for Ru–N. On top of that, the O–Ru–O angle increases over 25° and the dihedral angle decreases over 10°. These geometry observations show that PCET and 2PET steps involve significant changes in the geometry, while this is not the case for ET steps.

Given that the highest ρ of +0.95 is found for the oxidation of the lowest oxidation state Ru via an ET transfer, the influence of π-backbonding from the metal center to the bpy ligands must play a major role in the observed electronic effects. The π-backbonding effect decreases with an increasing oxidation state of Ru and an increase of the π-donor strength of the hydroxide and oxide ligands. Eventually, π-backbonding toward the bipyridine ligand becomes difficult for the Ru center in the + VI oxidation state as the remaining two d-electrons are located in nonbonding orbitals.^48^ This decrease in π-backbonding affinity is illustrated by the decreasing ρ with increasing oxidation states.

### Correlations between the Electronic Structure and Energetics of the Catalytic Steps

Our findings illustrate how the Pourbaix and Hammett plots form a huge 3D *E* vs pH vs ρ network, wherein all local minima of the potential energy landscape of water oxidation catalysis mediated by the Ru(bpy)_2_ family of complexes can be described. The acidic part of these diagrams shows reaction sequences wherein four sequential PCET pathways take place that all occur in a relatively small *E*-window, especially in the case of electron-withdrawing substituents such as R=CF_3_. In contrast, at the alkaline area of the diagram the oxidation of Ru(II) species and the formation of Ru(VI) species can easily be 1.5 V apart. Because all elementary steps are affected differently by these electronic substituents, there are significant shifts of the pH window in which particular reaction sequences occur as a function of EWGs and EDGs. More dramatic particular reaction sequences may completely disappear from the diagram for particular EWGs, while new, and previously not documented, reaction sequences can be observed instead. This illustrates that the Hammett parameter adds an intriguing new dimension in the case of a four-electron-transfer *E* vs pH diagram, thereby proving significant new insights.

For all p*K*_a_ values abstracted from the Pourbaix diagrams electronic effects by the substituent with a negative ρ were found, which is in line with negative ρ dependences found for the p*K*_a_ of organic molecules like benzoic acid,^57,58^ aniline,^59^ phenol,^60^ phenyl boronic acid,^61^ and benzoxaboroles.^62^ Since the aqua or hydroxyl ligand is located *trans* to the substituted bipyridine ligand in an octahedral geometry, the EDGs or EWGs directly affect the O–H bond of the aqua or hydroxyl ligand. Overall, the substituent effects on the p*K*_a_ values should not be underestimated because the different protonation states directly influence the operational mechanistic pathway. Such p*K*_a_ values, for example, directly relate the area between PCET and ET steps, resulting in significantly different *E* vs pH behavior and thus overpotentials.

The WO activity of the catalyst is strongly affected by the number of d-electrons at the metal site, which is related to the charge of the Ru complex. Although *cis*-[Ru^II^(4,4′-Cl_2_-bpy)_2_(H_2_O)_2_]^2+^ forms the Ru(=O)_2_ intermediate already in the +IV oxidation state, this species is incapable of oxidizing water. By increasing the charge of the Ru complex to +2, for Ru^VI^(=O)_2_, the complex becomes more electrophilic, which lowers the energy barrier for a water nucleophilic attack. For efficient water oxidation catalysis the trick is to obtain such a species at the lowest possible overpotential. The results show that incorporation of electronic substituents does not decrease the oxidation potential of high-oxidation-state PCET steps, resulting in an overpotential that is constant irrespective of the substituent introduced on the bipyridine ligand. Nonetheless, the use of substituents on the ligand framework can expand the PCET window over a broader pH range. If the Ru^VI^(=O)_2_ species is formed via an ET step, the oxidation potential at which this occurs can be adjusted by introduction of substituents on the ligand, although by definition, a higher overpotential is required for an ET process compared to a PCET process that produces the same species. WO catalysis may in principle be possible via a Ru=O intermediate with a lower oxidation state, thereby allowing for a decrease of the overpotential. However, due to the low charge of the Ru complex there is a lack of reactivity as the Ru=O moiety is not electrophilic enough to undergo a WNA.

Minor effects were found on the kinetics of the WO reaction by changes in the electronic structure. Substituents on the bpy ligand influence the π-acceptor strength of the ligand and, as a result, the π-donation from the oxo group to ruthenium. The catalytic activity therefore increases with EWG and diminishes with EDG in a pH 2.5 solution.

### Scaling Effects

**Figure 6:**
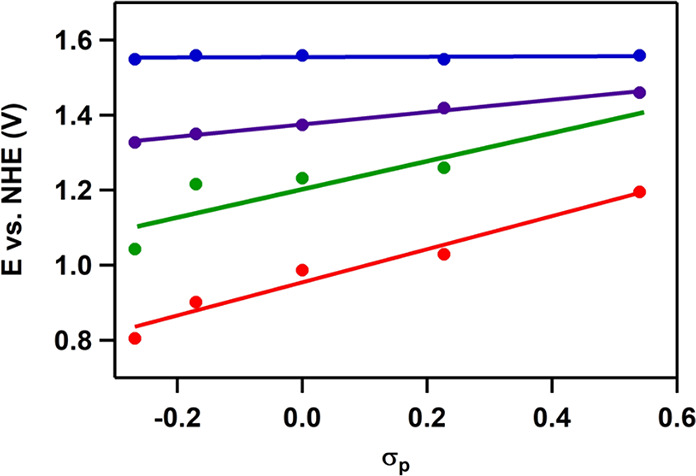
*E*_1/2_ values of the Ru^II^/Ru^III^ (red), Ru^III^/Ru^IV^ (green), Ru^IV^/Ru^V^ (purple), and Ru^V^/^VI^ (blue) redox couples reacting via PCET steps as a function of σ_p_.

Given that the low-oxidation-state species are more significantly affected by electronic effects, introducing EWGs results in a shift of all redox couples via PCET to higher potentials, apart from the +V/+VI couple (Scheme 2 and Scheme 4, mechanism 2). Consequently, all four redox steps of the catalytic WO reaction are found in a narrow potential window of 360 mV in the case of R = CF_3_ in the pH window of 1–2.4 (Figure 4e). This is more or less the limit to which four sequential redox events can be pushed together according to scaling relations in heterogeneous catalysis.^20^ Although we have not been able to push the redox potential of the individual redox steps further toward each other upon installing substituents with even higher σ_p_, extrapolation of our results indicates that all redox potentials can be pushed even closer to each other (Figure 6). In other words, scaling relations similar to those documented in heterogeneous catalysis do not seem to be at play for these *cis*-[Ru(bpy)_2_(H_2_O)]^2+^ catalysts. Obtaining an electrochemically “*ideal*” catalyst that has all the redox couples of the catalytic cycle leveled at the same redox potential thus seems to be possible in the case of homogeneous catalysis. However, the window in which the particular family of catalysts under study should be operational shifts rapidly due to the acidity of the catalytic species also being heavily affected by σ_p_. In the case of *cis*-[Ru(bpy)_2_(H_2_O)]^2+^ we anticipate finding the most ideal catalytic behavior at an operating window around pH −3, which is not a realistic value. It is ironic that despite having been able to decrease the potential window wherein all elementary steps take place from 800 mV (R = OMe) to 360 mV (R = CF_3_), the redox potential and activity of the +VI oxidation state species remains relatively unaffected. Recent efforts by the scientific community to introduce redox-active ligands to accommodate the more difficult oxidation steps^63−65^ and proton shuttles to facilitate the kinetically cumbersome O–O bond formation^51,66,67^ certainly may aid in overcoming these issues.

## Conclusion

The influence of electronic effects by the introduction of EDGs and EWGs on the catalyst scaffold system *cis*-[Ru(bpy)_2_(H_2_O)]^2+^ was investigated through thermodynamic and kinetic studies of all elementary steps involved during WO catalysis. The PCET steps are relatively insensitive with respect to σ_p_, while the decoupled ET and PT steps are strongly affected by σ_p_. As a direct consequence, significant changes in the electronic structure of the catalyst caused by substituent modifications give access to new and alternative reaction pathways. However, the sensitivity on σ_p_ decreases with increasing oxidation state, which is due to the lack of d-electrons available for π-backbonding. Yet, empty d-orbitals are required to obtain a sufficiently electrophilic ruthenium oxo species to form the actual O–O bond. Upon introduction of EWGs, the window in which all four redox couples can be found can be significantly decreased, and extrapolation of our data suggests that a limit has not yet been reached. This suggests that the particular Ru(bpy) system may not necessarily suffer from highly unfavorable linear scaling of the individual redox steps through electronic effects, which offers a significant advantage over heterogeneous catalysis. Nevertheless, the correlations between the electronic structure and the energetics of the elementary steps necessary for turnover, as we have unraveled in this study, do show results that it remains highly challenging to design an “ideal” catalyst that (1) catalyzes the WO reaction near the equilibrium potential of water; (2) operates in a realistic pH window; and (3) is synthetically accessible and sufficiently stable. We believe that thorough systematic studies aimed at mapping *E* vs pH and ρ for multiple elementary steps simultaneously as pioneered in this study are crucial to pinpoint the design principles for such “ideal” catalysts that will eventually allow for efficient and economically viable water splitting.

## Supporting Information Available

Experimental, synthetic protocols, additional figures, and NMR spectra (PDF)

## ABBREVIATIONS

2PET: two-protons-one-electron transfer
BDD: boron-doped diamond
CE: counter electrode
CV: cyclic voltammogram
DPV: differential pulse voltammetry
EDGs: electron-donating groups
ET: electron transfer
EWGs: electron-withdrawing groups
GC: glassy carbon
MLCT: metal-to-ligand charge transfer
NHE: normal hydrogen electrode
PCET: proton-coupled electron transfer
PT: proton transfer
RE: reference electrode
RHE: reversible hydrogen electrode
WNA: water nucleophilic attack
WO: water oxidation
