# Supplementary material for: Correlations between the Electronic Structure and Energetics of the Catalytic Steps in Homogeneous Water Oxidation Catalysis
Source: J Am Chem Soc. 2023 Oct 10;145(42):23057–67. doi: 10.1021/jacs.3c05741 (PMC10603781; doi:10.1021/jacs.3c05741)
Supplement: Supplementary file 1 — ja3c05741_si_001.pdf [file ja3c05741_si_001.pdf]

Supporting information

Correlations between the electronic structure and energetics of the catalytic steps in homogeneous water oxidation catalysis

*Daan den Boer and Dennis G. H. Hetterscheid**

Leiden Institute of Chemistry, Leiden University, Einsteinweg 55, 2300 RA, Leiden, The Netherlands

**Corresponding author:* d.g.h.hetterscheid@chem.leidenuniv.nl

Contents

I. Experimental information 3
General information 3
Synthetic protocols 5
II. UV-vis 8
III. Construction of Pourbaix diagrams 9
IV. Electrochemistry 10
Background currents 10
Catalytic activity for Ru(V) 11
Hammett correlations 12
Kinetic analysis 13
V Stability 16
VI. NMR spectra 17
VI. DPV data used to construct Pourbaix Diagrams 29
VII. References 68

I. Experimental information

General information

All chemicals used were commercially purchased and used without any further purification. Dichloro(1,5-cyclooctadiene)ruthenium(II) was purchased from Fluorchem, 4,4'-dichloro-2,2'-bipyridine was purchased from Biosynth Carbosynth and 4,4'-bis(trifluormethyl)-2,2'-bipyridine was purchased from Strem Chemicals Inc. All other chemicals were purchased from Sigma Aldrich. 1,2-dichlorobenzene was dried on 3 Å molecular sieves (Alfa Aeser). *cis*-[Ru(bpy)$_2$Cl$_2$] was commercially purchased and not synthesized (Sigma Aldrich). All Ru complexes discussed were racemic mixtures of the Λ and Δ enantiomers. ^{1}H , ^{13}C APT and ^{19}F NMR spectra were recorded at room temperature on a Bruker AV-400 (400/101 MHz) or Bruker AV-500 (500 MHz) using the residual solvent as internal standard (DMSO-*d*6 or D_2O). Mass spectra were obtained by LC-MS (Thermo Finnigan AQA ESI-MS). Elemental analysis was performed by Mikroanalytisches Laboratorium Kolbe in Oberhausen, Germany. UV-Vis spectra were recorded on a Varian Cary 50 Spectrophotometer in a quartz cuvette (path length 1 cm).

UV-vis spectra were recorded in Milli-Q water between 200 and 800 nm. For each *cis*-[Ru(4,4'-R$_2$-bpy)$_2$(H$_2$O)]$^{2+}$ compound a 10 mL 0.5 mM stock solution was prepared. This stock solution was diluted 20 times to obtain a 25 µM solution, suitable for UV-vis analysis. The solutions were kept in the dark by wrapping the solutions in aluminum foil. This was done to prevent *cis* to *trans* isomerization of all Ru compounds.[1]

Electrochemical solutions were prepared using high-purity chemicals (Suprapur® grade, Merck): H_3PO_4 (85% aq.), NaH_2PO_4 (99.99%), Na_2HPO_4 (99.99%), Na_2SO_4 (99.99%), $NaOH\cdot H_2O$ (99.995%) and HNO_3 (65% aq.). The pH of the electrochemical solutions was measured with a Radiometer PHM220, which was calibrated using IUPAC buffers. In some cases, the ionic strength of the buffers was adjusted by addition of Na_2SO_4.
Custom made single-compartment glass cells with a three-electrode setup were used to perform experiments. For the cleaning of glassware and preparation of electrochemical solutions, Milli-Q ultrapure water was used (resistivity >18.2 MΩ·cm). Electrochemical glassware was routinely cleaned by storing the glassware in $KMnO_4$ solution (1 g/L $KMnO_4$ in 0.5 M H_2SO_4) overnight, followed by rinsing the glassware with Milli-Q water. Manganese byproducts were removed by storing the glassware in water for 30 minutes with addition of ~20 mL of H_2O_2 (35% in aq.) and a few drops of H_2SO_4. Afterwards, all glassware was rinsed thoroughly with water and boiled two or three times in Milli-Q water. Prior to each electrochemical experiment the glassware was again boiled in Milli-Q water and rinsed thoroughly.

Electrochemical experiments were performed on Autolab potentiostats controlled by NOVA 2.0 software. For DPV experiments the following settings were used: Potential step: 0.001 V, Modulation amplitude: 0.05 V, Modulation time: 0.003 s, Interval time: 0.05 s using Nova 2.0 software.

Electrochemical solutions were deaerated by bubbling argon (Linde, 5.0) through the solution for 20 minutes before starting the experiment. During the experiment, the electrochemical cell was under a continuous flow of argon, to prevent air flowing into the cell. Electrochemical solutions containing Ru-based catalysts were stored in brown colored volumetric flasks (VWR) and electrochemical cells were wrapped in alumina foil to prevent light induced *cis* to *trans* isomerization.[1]

Glassy carbon (Autolab) and BDD (Windsor scientific Ltd) were used as working electrode. Both electrodes had a geometric surface of 0.07 cm^2. The GC working electrode was manually polished on a microcloth using alumina slurries of 1.0, 0,3, and 0.05 µm, respectively (Buehler). The excess of polish material was removed by rinsing the electrode with water and subsequently sonicating the electrode in water for 10 minutes. The BDD electrode was sonicated in water for 10 minutes and subsequently electro polished by 200 cycles in 0.1 M HNO_3 between –1.0 to 2.25 V vs. RHE with a scan rate of 1000 mV/s. A gold wire (MaTeck) was used as counter electrode. Prior to the experiment the electrode was rinsed with water and flame-annealed. The reference electrode was a RHE consisting of a Pt wire and mesh (MaTeck) in a blank solution saturated with H_2 (Linde, 5.0) containing the same pH as the bulk electrolyte. The RHE electrode and bulk solution were connected via a Luggin capillary. The reference potential was converted to NHE.

Synthetic protocols

cis-Dichlorido-bis(4,4'-dimethyl-2,2'-bipyridine)ruthenium(II)

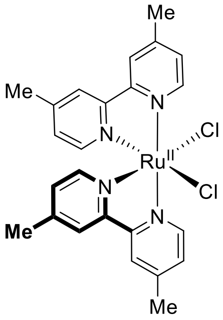

The following procedure was based on methods described in literature.[2-4] Dichloro(1,5-cyclooctadiene)ruthenium(II) (0.25 g, 0.90 mmol, 1 equiv.), 4,4'-dimethyl-2,2'-bipyridine (0.33 g, 1.82 mmol, 2 equiv.) and dry 1,2-dichlorobenzene (3.5 mL) were added to a flame dried flask in the dark and under nitrogen atmosphere. The mixture was deoxygenated by bubbling nitrogen through the mixture for 30 minutes. The mixture was refluxed for 2.5 hours and was allowed to cool to room temperature. Diethyl ether (~30 mL) was added to the mixture and the mixture was filtered over a Büchner filter. The solid product was washed thoroughly with diethyl ether and dried *in vacuo* at 40°C to yield a dark purple powder (0.32 g, 0.58 mmol, 65%). ^{1}H NMR (400 MHz, DMSO-d_6) δ 9.77 (d, *J* = 5.9 Hz, 2H), 8.47 (s, 2H), 8.31 (s, 2H), 7.59 (dd, *J* = 6.1, 1.5 Hz, 2H), 7.31 (d, *J* = 5.9 Hz, 2H), 6.92 (dd, *J* = 6.6, 1.9 Hz, 2H), 2.61 (s, 6H, Me), 2.33 (s, 6H, Me). The H-NMR spectrum was in agreement with previous reported data.[5]

cis-Dichlorido-bis(4,4'-dimethoxy-2,2'-bipyridine)ruthenium(II)

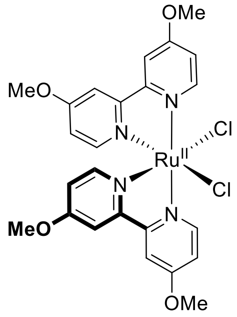

This compound has been reported previously,[6] and was obtained via the same protocol as was employed for *cis*-[Ru(4,4'-Me_2-bpy)$_2Cl_2$]. Reagents: Dichloro(1,5-cyclooctadiene)ruthenium(II) (0.49 g, 1.77 mmol, 1 equiv.), 4,4'-dimethoxy-2,2-bipyridine (0.76 g, 3.53 mmol, 2 equiv.) and 1,2-dichlorobenzene (3.5 mL). Yield: 0.83 g, 1.37 mmol, 78%. ^{1}H NMR (400 MHz, DMSO-d_6) δ 9.71 (d, *J* = 6.6 Hz, 2H), 8.27 (d, *J* = 2.8 Hz, 2H), 8.12 (d, *J* = 2.8 Hz, 2H), 7.43 (dd, *J* = 6.6, 2.7 Hz, 2H), 7.29 (d, *J* = 6.6 Hz, 2H), 6.79 (dd, *J* = 6.7, 2.8 Hz, 2H), 4.07 (s, 6H), 3.86 (s, 6H).

cis-Dichlorido-bis(4,4'-dichloro-2,2'-bipyridine)ruthenium(II)

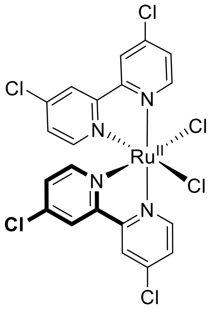

This compound has been reported previously,[7] and was obtained via the same protocol as was employed for *cis*-[Ru(4,4'-Me_2-bpy)$_2Cl_2$]. Reagents: Dichloro(1,5-cyclooctadiene)ruthenium(II) (0.24 g, 0.87 mmol, 1 equiv.), 4,4'-dichloro-2,2-bipyridine (0.40 g, 1.79 mmol, 2 equiv.) and 1,2-dichlorobenzene (35 mL). Yield: 0.49 g, 0.87 mmol, 91%. ^{1}H NMR (400 MHz, DMSO-d_6) δ 9.83 (d, *J* = 6.3 Hz, 2H), 8.99 (d, *J* = 2.3 Hz, 2H), 8.84 (d, *J* = 2.4 Hz, 2H), 7.98 (dd, *J* = 6.2, 2.3 Hz, 2H), 7.59 (d, *J* = 6.3 Hz, 2H), 7.26 (dd, *J* = 6.3, 2.4 Hz, 2H). ^{13}C NMR (101 MHz, DMSO-d_6) δ 160.51, 158.82, 153.53, 141.57, 140.21, 126.09, 125.80, 123.87, 123.56

cis-Dichlorido-bis(4,4′-bis(trifluormethyl)-2,2′-bipyridine)ruthenium(II)

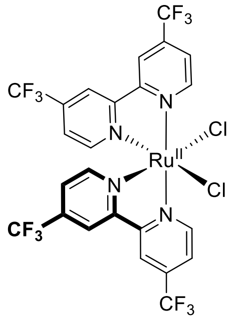

This compound has been reported previously,[8] and was obtained via the same protocol as was employed for *cis*-[Ru(4,4′-Me_2-bpy)$_2Cl_2$]. Reagents: Dichloro(1,5-cyclooctadiene)ruthenium(II) (0.12 g, 0.43 mmol, 1 equiv.), 4,4′-bis(trifluormethyl)-2,2′-bipyridine (0.25 g, 0.86 mmol, 2 equiv.) and 1,2-dichlorobenzene (5 mL). Yield: 0.18 g, 0.24 mmol, 56%. <u>^{1}H NMR</u> (500 MHz, DMSO-d_6) δ 10.14 (d, *J* = 5.9 Hz, 2H), 9.41 (s, 2H), 9.25 (s, 2H), 8.26 (d, *J* = 5.2 Hz, 1H), 7.94 (d, *J* = 6.1 Hz, 2H), 7.48 (dd, *J* = 6.3, 2.0 Hz, 2H). <u>^{19}F NMR</u> (471 MHz, DMSO) δ -62.25 (s, 6F), -62.60 (s, 6F).

cis-Bis(2,2′-bipyridine)bis(trifluoromethanesulfonato)ruthenium(II)

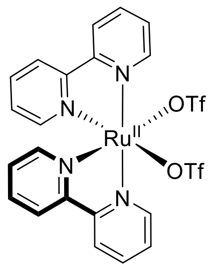

The following procedure was based on methods described in literature.*[9-10]* *cis*-[Ru(bpy)$_2Cl_2$] (0.21 g, 0.44 mmol, 1 equiv.) was added to a round bottom flask under nitrogen atmosphere. Deoxygenated and dry 1,2-dichlorobenzene (20 mL, 30 min purged with nitrogen) was added to the flask and the mixture was stirred under nitrogen atmosphere. Triflic acid (0.70 mL, 1.19 mmol, 17.8 equiv.) was added to the mixture and the color changed from dark purple to red. **CAUTION*:*** *triflic acid is a strong corrosive acid*. The mixture was stirred in the dark at room temperature for 1 hour, followed by filtration over a Büchner filter. The remaining solid was thoroughly washed with diethyl ether and dried *in vacuo* at 40°C to yield a burgundy red powder (0.081 g, 0.11 mmol, 26%). <u>^{1}H NMR</u> (400 MHz, D_2O) δ 9.31 (d, *J* = 5.3 Hz, 2H), 8.52 (d, *J* = 8.2 Hz, 2H), 8.31 (d, *J* = 8.1 Hz, 2H), 8.23 – 8.14 (m, 2H), 7.83 (t, *J* = 6.6 Hz, 2H), 7.72 (t, *J* = 7.8 Hz, 2H), 7.68 (d, *J* = 5.7 Hz, 2H), 7.08 – 7.00 (m, 2H). ^{1}H-NMR Spectra in D_2O are in agreement with spectra of c*is*-[Ru(bpy)$_2$(H_2O)$_2$](X), where X represents (OTf)$_2$ or SO_4^{2-} anions.[11-12] <u>MS (ESI)</u> m/z: calc. for [Ru^{II}(bpy)$_2$(MeCN)(H_2O)]$^{2+}$: 236.5, found 236.3; calc. for [Ru^{II}(bpy)$_2$(MeCN)$_2$]$^{2+}$: 248.0, found 247.8; calc. for [Ru^{II}(bpy)$_2$(OTf)]$^+$: 563.0, found 562.8; calc. for [Ru^{II}(bpy)$_2$(MeCN)(OTf)]$^+$: 604.0, found 603.9. <u>UV-vis</u> absorption peaks (Milli-Q water): 243, 291, 340, and 487 nm. <u>Elemental analysis</u>: calc. for $C_{22}H_{16}F_6N_4O_6RuS_2\cdot 0.2H_2O$: C 36.95, H 2.31, N 7.83%; found: C 36.97, H 2.31, N 7.81%.

cis-Bis(4,4′-dimethyl-2,2′-bipyridine)bis(trifluoromethanesulfonato)ruthenium(II)

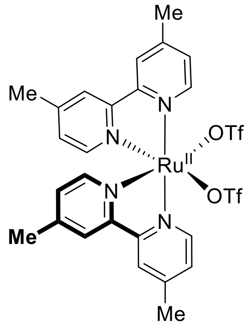

The synthetic protocol for *cis*-[Ru(bpy)$_2$(OTf)$_2$] was followed. Reagents: *cis*-[Ru(4,4′-Me_2-bpy)$_2Cl_2$] (0.22 g, 0.40 mmol, 1 equiv.), 1,2-dichlorobenzene (18 mL) and triflic acid (0.17 mL, 1.93 mmol, 4.8 equiv.). Yield: 0.078 g, 1.0 mmol, 25%. <u>^{1}H NMR</u> (400 MHz, D_2O) δ 9.10 (d, *J* = 5.7 Hz, 2H), 8.34 (s, 2H), 8.13 (s, 2H), 7.66 (dd, *J* = 5.8, 0.9 Hz, 2H), 7.45 (d, *J* = 5.9 Hz, 2H), 6.88 (dd, *J* = 6.0, 1.1 Hz, 2H), 2.66 (s, 6H), 2.37 (s, 6H). <u>MS (ESI)</u> m/z: calc. for [Ru^{II}(4,4′-Me_2-bpy)$_2$(H_2O)(MeCN)]$^{2+}$: 264.6, found 264.2; calc. for [Ru^{II}(4,4′-Me_2-bpy)$_2$(MeCN)]$^{2+}$: 276.1, found 275.7; calc. for [Ru^{II}(4,4′-Me_2-bpy)$_2$(OTf)]$^+$: 619.0, found 618.1; calc. for [Ru^{II}(4,4′-Me_2-bpy)$_2$(OTf)(MeCN)]$^+$: 660.1, found 660.2. <u>UV-vis</u> absorption peaks

(Milli-Q water): 206, 248, 289, 336, and 481 nm. Elemental analysis: calc. for $C_{26}H_{24}F_6N_4O_6RuS_2 \cdot 0.2\ H_2O$: C 40.48, H 3.19, N 7.26%; found: C 40.20, H 3.15, N 7.21%.

cis-Bis(4,4'-dimethoxy-2,2'-bipyridine)bis(trifluoromethanesulfonato)ruthenium(II)

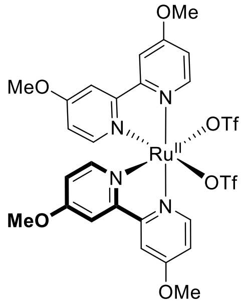

The synthetic protocol for *cis*-$[Ru(bpy)_2(OTf)_2]$ was followed. Reagents: *cis*-$[Ru(4,4'\text{-}(OMe)_2\text{-}bpy)_2Cl_2]$ (0.20 g, 0.33 mmol, 1 equiv.), 1,2-dichlorobenzene (16 mL) and triflic acid (0.15 mL, 1.70 mmol, 5.1 equiv.). Yield: 0.19 g 0.23 mmol, 71%. ^{1}H NMR (400 MHz, D_2O) δ 9.07 (d, *J* = 6.5 Hz, 2H), 8.02 (d, *J* = 2.7 Hz, 2H), 7.84 (d, *J* = 2.8 Hz, 2H), 7.49 (d, *J* = 6.7 Hz, 2H), 7.43 (dd, *J* = 6.5, 2.7 Hz, 2H), 6.70 (dd, *J* = 6.7, 2.8 Hz, 2H), 4.10 (s, 6H), 3.86 (s, 6H). MS (ESI) m/z: calc. for $[Ru^{II}(4,4'\text{-}(OMe)_2\text{-}bpy)_2(H_2O)(MeCN)]^{2+}$: 296.3, found 296.3; calc. for $[Ru^{II}(4,4'\text{-}(OMe)_2\text{-}bpy)_2(MeCN)_2]^{2+}$: 308.1, found 308.0; calc. for $[Ru^{II}(4,4'\text{-}(OMe)_2\text{-}bpy)_2(OTf)]^{+}$: 683.0, found 683.0; calc. for $[Ru^{II}(4,4'\text{-}(OMe)_2\text{-}bpy)_2(MeCN)(OTf)]^{+}$: 724.1, found 724.1. UV-vis absorption peaks (Milli-Q water): 209, 224, 266, 333, and 479 nm. Elemental analysis: calc. for $C_{26}H_{24}F_6N_4O_{10}RuS_2 \cdot 1.5\ H_2O$: C 36.26, H 3.16, N 6.52%; found: C 36.28, H 3.14, N 6.45%.

cis-Bis(4,4'-dichloro-2,2'-bipyridine)bis(trifluoromethanesulfonato)ruthenium(II)

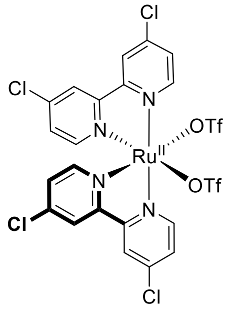

The synthetic protocol for *cis*-$[Ru(bpy)_2(OTf)_2]$ was followed. Reagents: *cis*-$[Ru(4,4'\text{-}(OMe)_2\text{-}bpy)_2Cl_2]$ (0.20 g, 0.32 mmol, 1 equiv.), 1,2-dichlorobenzene (20 mL) and triflic acid (0.14 mL, 1.61 mmol, 5.0 equiv.). Yield: 0.22 g 0.26 mmol, 80%. ^{1}H NMR (400 MHz, D_2O) δ 9.21 (d, *J* = 6.1 Hz, 2H), 8.63 (d, *J* = 2.1 Hz, 2H), 8.43 (d, *J* = 2.3 Hz, 2H), 7.94 (dd, *J* = 6.2, 2.1 Hz, 2H), 7.62 (d, *J* = 6.3 Hz, 2H), 7.20 (dd, *J* = 6.3, 2.2 Hz, 2H). MS (ESI) m/z: calc. for $[Ru^{II}(4,4'\text{-}Cl_2\text{-}bpy)_2(MeCN)(H_2O)]^{+}$: 304.9, found 305.3; calc. for $[Ru^{II}(4,4'\text{-}Cl_2\text{-}bpy)_2(MeCN)_2]^{+}$: 317.0, found 317.1; calc. for $[Ru^{II}(4,4'\text{-}Cl_2\text{-}bpy)_2(OTf)]^{+}$: 700.8, found 700.8; calc. for $[Ru^{II}(4,4'\text{-}Cl_2\text{-}bpy)_2(MeCN)(OTf)]^{+}$: 741.9, found 741.9. UV-vis absorption peaks (Milli-Q water): 216, 249, 293, 354, and 505 nm. Elemental analysis: calc. for $C_{22}H_{12}Cl_4F_6N_4O_6RuS_2 \cdot 0.15\ H_2O$: C 31.01, H 1.46, N 6.58%; found: C 30.70, H 1.41, N 6.44%.

cis-Bis(4,4'-bis(trifluormethyl)-2,2'-bipyridine)bis(trifluoromethanesulfonato)-ruthenium(II)

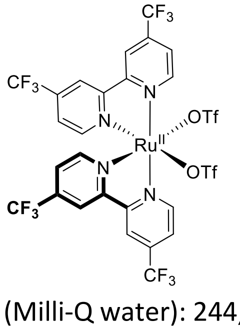

The synthetic protocol for *cis*-$[Ru(bpy)_2(OTf)_2]$ was followed. Reagents: *cis*-$[Ru(4,4'\text{-}(CF_3)_2\text{-}bpy)_2Cl_2]$ (0.10 g, 0.13 mmol, 1 equiv.), 1,2-dichlorobenzene (20 mL) and triflic acid (0.063 mL, 0.71 mmol, 5.5 equiv.). Yield: 0.074 g, 0.075 mmol, 58%. ^{1}H- NMR (500 MHz, D_2O) δ 9.62 (d, *J* = 5.9 Hz, 2H), 9.04 (s, 2H), 8.83 (s, 2H), 8.28 (d, *J* = 5.9 Hz, 2H), 7.94 (d, *J* = 6.1 Hz, 2H), 7.50 (d, *J* = 6.2 Hz, 2H). ^{19}F NMR (471 MHz, D_2O) δ -64.85 (s, 6F), -65.09 (s, 6F), -78.96 (s, 6F). MS (ESI) m/z: calc. for $[Ru^{II}(4,4'\text{-}(CF_3)_2\text{-}bpy)_2(MeCN)_2]^{+}$: 384.0, found 384.0. UV-vis absorption peaks (Milli-Q water): 244, 299, 360 and 505 nm.

II. UV-vis

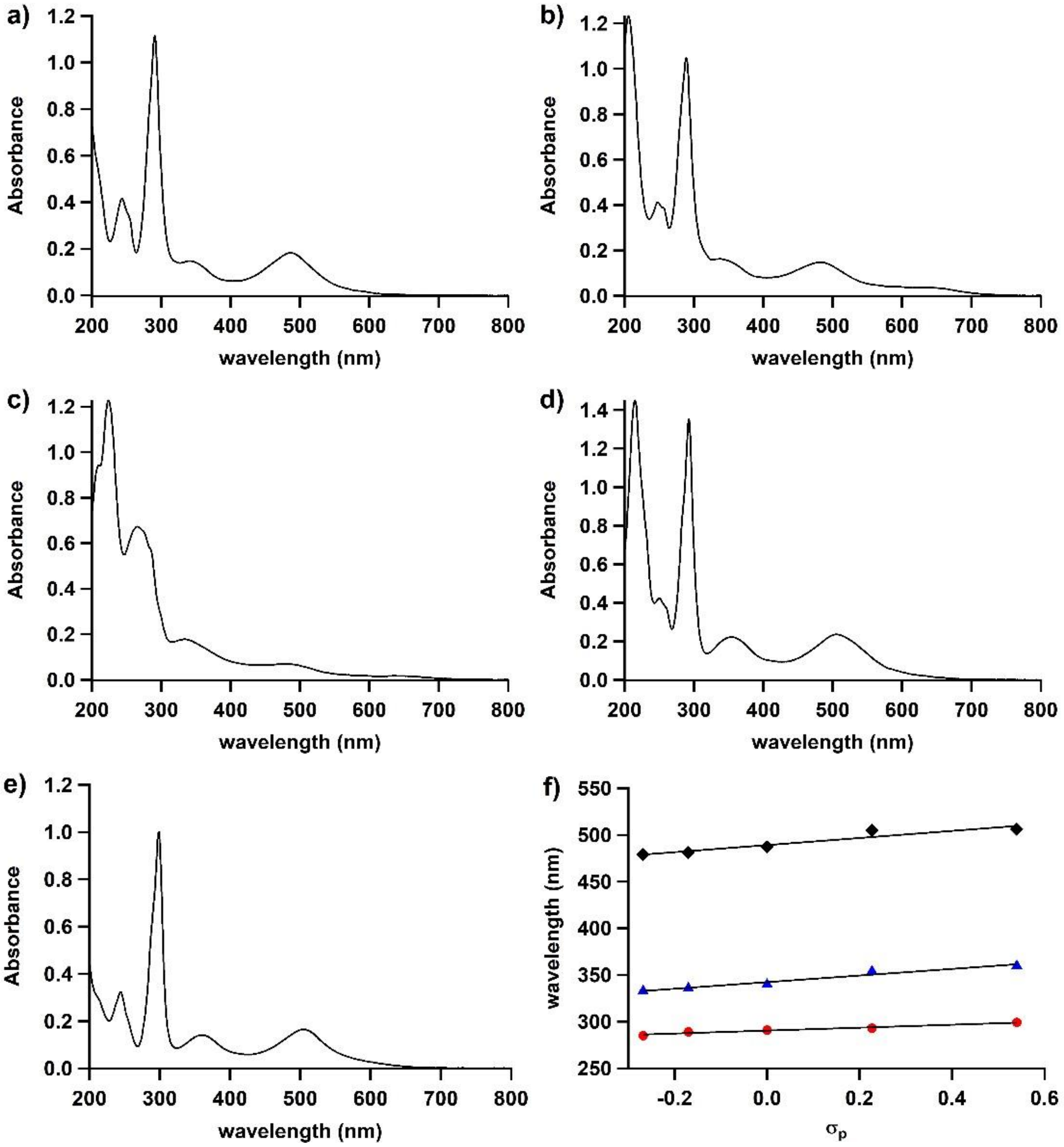

Figure S1. a-e) UV-vis spectra of 25 μM *cis*-$[Ru(4,4'\text{-}R_2\text{-bpy})_2(H_2O)_2]^{2+}$ in Milli-Q water in which R = H (a), Me (b), OMe (c), Cl (d) and CF_3 (e). f) Correlation of the absorbance bands assigned to the π–π* (red, R^2 = 0.96) and MLCT (blue, R^2 = 0.96; black, R^2 = 0.89) transitions with the Hammett parameter (σ_p).

III. Construction of Pourbaix diagrams

Electrochemical experiments were compared with the original constructed Pourbaix by Dobson and Meyer (Figure S2).[13] Data was extracted from the Pourbaix diagram using ScanIt software from amsterCHEM. Small differences in experimental settings were found. In our experiments, we used a boron doped diamond (BDD) or glassy carbon (GC) working electrode, Au wire counter electrode and a reversible hydrogen electrode (RHE). The group of Meyer used a GC working electrode, a Pt counter electrode and a saturated sodium chloride calomel electrode (SSCE).[14] The found reference potentials were converted to NHE by the following equations:

$$\mathrm{E\ (NHE) = E\ (RHE) - 0.059 \cdot pH = \ E(SSCE) + 0.236}$$

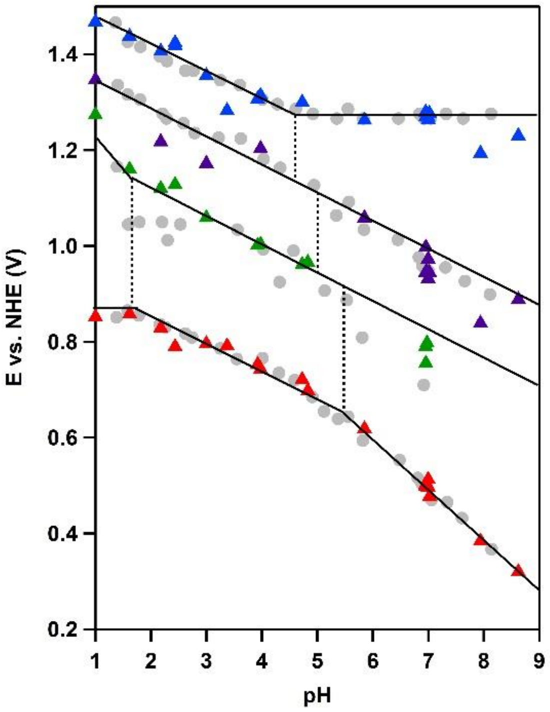

Figure S2. Pourbaix diagram of *cis*-$[Ru^{II}(bpy)_2(H_2O)_2]^{2+}$. Red, green, purple, blue corresponds to redox potentials obtained from cyclic and differential pulse voltammetry experiments for the $Ru^{II/III}$ to $Ru^{V/VI}$ redox couples, respectively. Grey points: original data points obtained from Dobson and Meyer.[13] All potentials were converted to NHE.

IV. Electrochemistry

Background currents

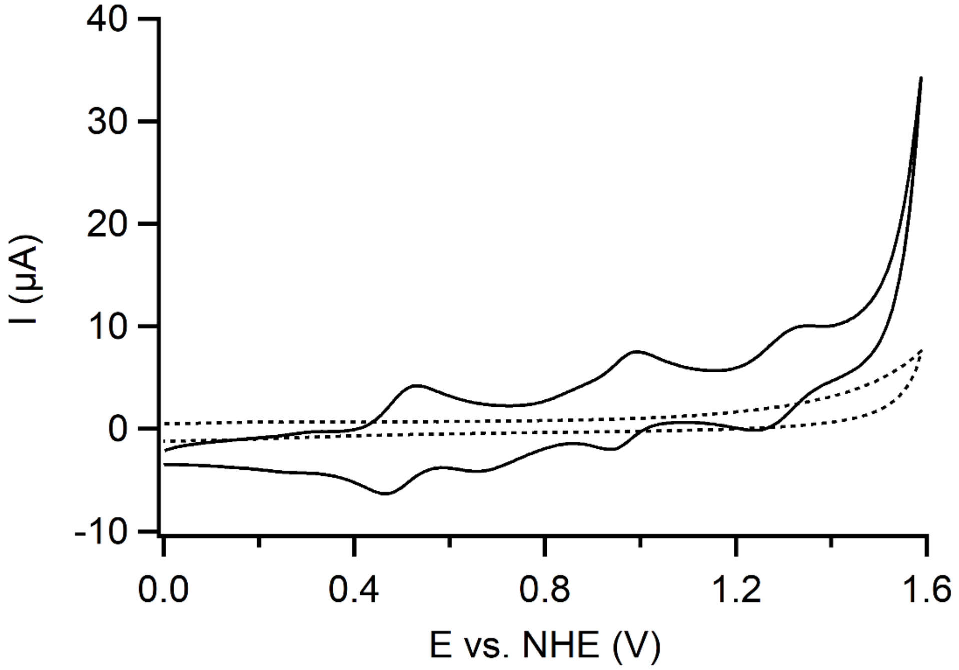

Figure S3. CV in presence (black line) of 0.5 mM *cis*-$[Ru^{II}(bpy)_2(H_2O)_2]^{2+}$ and without (dotted line) in 100 mM pH 7 phosphate buffer at a scan rate of 100 mV/s. GC, Au and RHE were used as WE, CE and RE, respectively. Potentials were converted to normal hydrogen electrode (NHE).

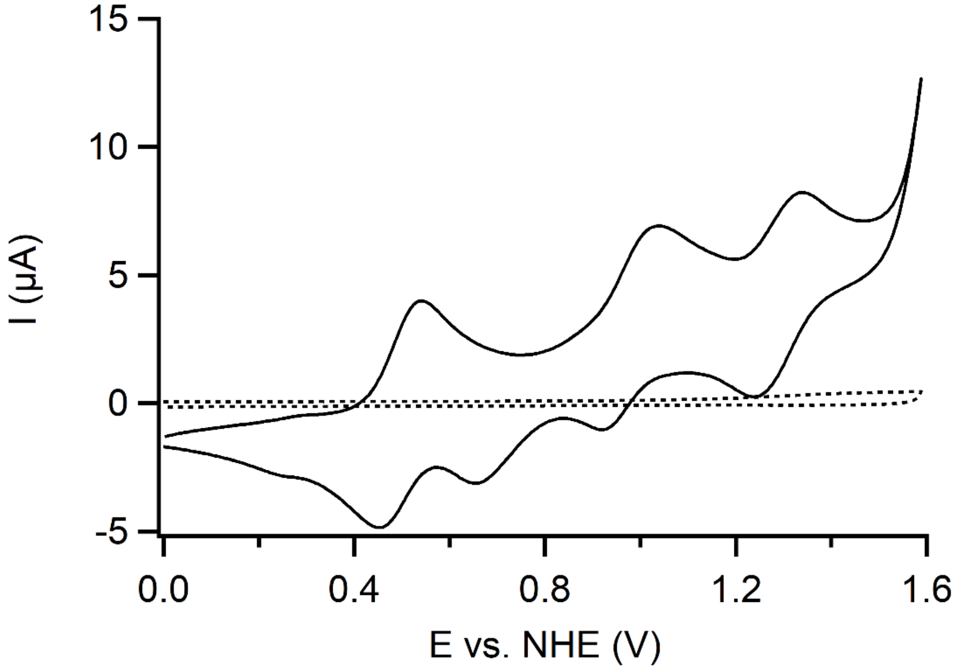

Figure S4. CV in presence (black line) of 0.5 mM *cis*-$[Ru^{II}(bpy)_2(H_2O)_2]^{2+}$ and without (dotted line) in 100 mM pH 7 phosphate buffer at a scan rate of 100 mV/s. BDD, Au and RHE were used as WE, CE and RE, respectively. Potentials were converted to normal hydrogen electrode (NHE).

Catalytic activity for Ru(V)

Two CVs of *cis*-$[Ru^{II}(4,4'\text{-}Cl_2\text{-bpy})_2(H_2O)_2]^{2+}$ in phosphate buffer of pH 5.9 and 11.4 were recorded (Figure A2). Both mechanisms proceed via the intermediate *cis*-$[Ru^{V}(4,4'\text{-}Cl_2\text{-bpy})_2(=O)_2]^{2+}$. However, at pH 11.4, a catalytic wave is present before the redox couple of $Ru^{V/VI}$.

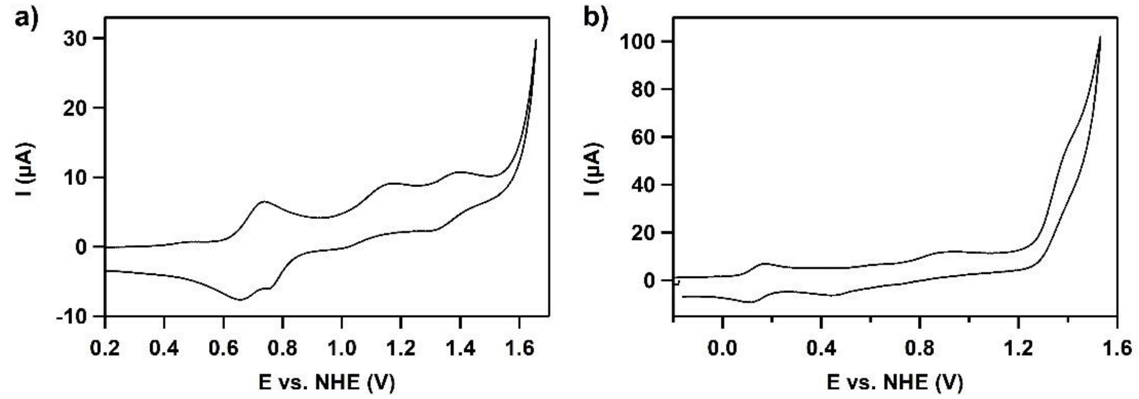

Figure S5. CV of 1.0 mM *cis*-$[Ru^{II}(4,4'\text{-}Cl_2\text{-bpy})_2(H_2O)_2]^{2+}$ in 100 mM phosphate buffer pH 5.9 (a) and 11.4 (b) at a scan rate of 100 mV/s. GC, Au and RHE were used as WE, CE and RE, respectively. Potentials were converted to NHE.

Hammett correlations

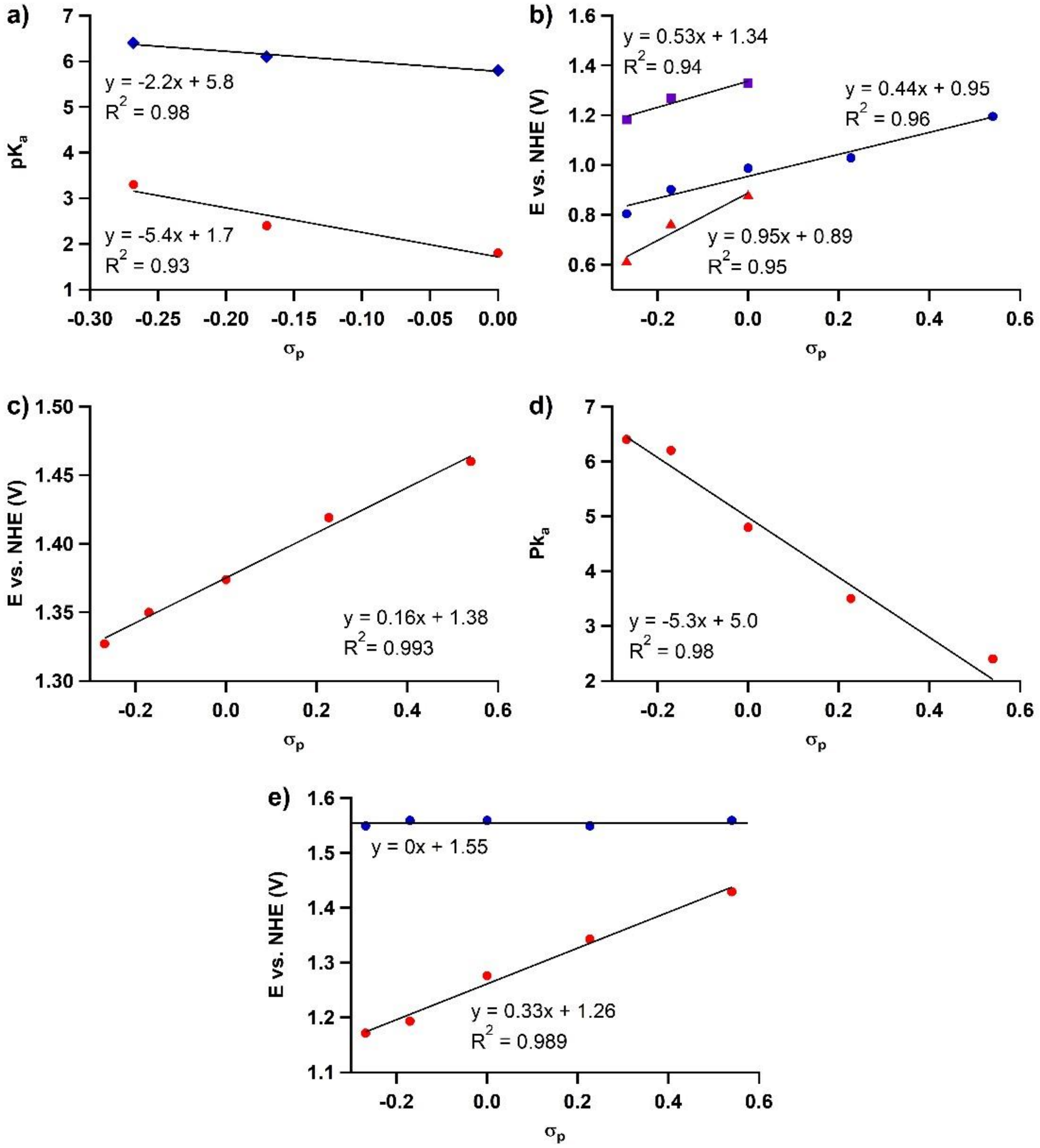

Figure S6. a) pK_a values of $Ru^{III}(OH_2)_2/Ru^{III}(OH)(OH_2)$, in red and $Ru^{III}(OH)(OH_2)/Ru^{III}(OH)_2$, in blue versus the Hammett parameter with ρ values of –5.4 and –2.2, respectively. b) The potential of the Ru^{II}/Ru^{III} redox couple proceeding via ET (red), PCET (blue) and 2PET (purple) versus the Hammett parameter with ρ values of +0.95, +0.44 and +0.53, respectively. c) The potential of the Ru^{IV}/Ru^{V} redox couple proceeding via PCET versus the Hammett parameter with a ρ value of +0.16. d) The pK_a value of $Ru^{V}(=O)(OH)/Ru^{V}(=O)_2$, versus the Hammett parameter with a ρ value of –5.3. e) The potential of the Ru^{V}/ Ru^{VI} redox couple proceeding via PCET (blue) in mechanism 1 and 2 and ET (red) in mechanism 3 versus the Hammett parameter with ρ values of 0 and +0.33, respectively.

Kinetic analysis

CVs of *cis*-$[Ru^{II}(4,4'\text{-}R_2\text{-bpy})_2(H_2O)_2]^{2+}$ derivatives (R = Me, OMe and Cl) were measured in 100 mM phosphate buffers of pH 2.5. The ionic strength of both buffers was adjusted to 0.3 M, by addition of Na_2SO_4. As no limiting plateau current was achieved in the catalytic wave and no redox couple is observed underneath the catalytic wave, implementation of foot-of-the-wave analysis is non-applicable.[15]

Comparisons of obtained catalytic current at a certain potential was also non-applicable as redox processes were unequally fast due to differences in the diffusion coefficient (Table S1). CVs were recorded of the $Ru^{II/III}$ redox couples of all *cis*-$[Ru^{II}(4,4'\text{-}R_2\text{-bpy})_2(H_2O)_2]^{2+}$ with different scan rates in a random order in order to easily distinguish between scan rate and time dependent processes.[16] The peak currents of the oxidative and reductive wave were plotted versus the square root of the scan rate (Figure A3-A6). The diffusion coefficient was calculated following the Randles–Ševčík equation:

$$i_p = 0.4463 \cdot n \cdot F \cdot A \cdot C \cdot \sqrt{\frac{nFvD}{RT}}$$

The equation could be rewritten for standard conditions (25°C):

$$i_p = 269000 \cdot n^{\frac{3}{2}} \cdot A \cdot C \cdot \sqrt{D} \cdot \sqrt{v}$$

From the slope in the i_p versus the square root of the scan rate plot, the diffusion coefficient can be calculated. The slope in this plot is equal to:

$$slope = 269000 \cdot n^{\frac{3}{2}} \cdot A \cdot C \cdot \sqrt{D}$$

In these equations the quantities and units are: i_p – peak current (A) of the oxidation or reduction wave of the $Ru^{II/III}$ redox couple; n – number of electrons involved in the redox event, which was 1 in this case; F – Faraday constant (96485 $C \cdot mol^{-1}$); A – (geometric) electrode surface area (0.071 cm^2 for the used GC electrode); C – concentration of [Ru] ($0.5 \cdot 10^{-6}$ $mol \cdot cm^{-3}$ in this case); v – scan rate ($V \cdot s^{-1}$); R – universal gas constant (8.314 $J \cdot mol^{-1} \cdot K^{-1}$); T – absolute temperature (298.15 K); D – diffusion coefficient ($cm^2 \cdot s^{-1}$).

Table S1. Diffusion coefficients determined for *cis*-[Ru^{II}(4,4'-R_2-bpy)$_2$(H_2O)$_2$]$^{2+}$ derivatives.

**Substituent R =**	**$D_{oxidation}$ $Ru^{II/III}$ ($cm^2 \cdot s^{-1}$)**	**$D_{reduction}$ $Ru^{II/III}$ ($cm^2 \cdot s^{-1}$)**
**H**	$2.76 \cdot 10^{-6}$	$2.11 \cdot 10^{-6}$
**Me**	$1.98 \cdot 10^{-6}$	$1.60 \cdot 10^{-6}$
**OMe**	$5.77 \cdot 10^{-7}$	$5.89 \cdot 10^{-7}$
**Cl**	$3.38 \cdot 10^{-6}$	$1.97 \cdot 10^{-6}$

To compare catalytic activity between all *cis*-[Ru^{II}(4,4'-R_2-bpy)$_2$(H_2O)$_2$]$^{2+}$ derivatives, the current enhancement (i_{cat}/i_p) of the catalytic wave (i_{cat}) and the $Ru^{II/III}$ redox couple (i_p) was utilized. In this manner, a correction is applied for the differences in diffusion coefficient. To correct also for background processes, the current obtained in a blank CV, consisting of the corresponding phosphate buffer was subtracted. Hereby i_{cat} and i_p are defined as the current obtained in the CV within the presence of *cis*-[Ru^{II}(4,4'-R_2-bpy)$_2$(H_2O)$_2$]$^{2+}$, subtracted by the current in the blank CV, without the presence of *cis*-[Ru^{II}(4,4'-R_2-bpy)$_2$(H_2O)$_2$]$^{2+}$.

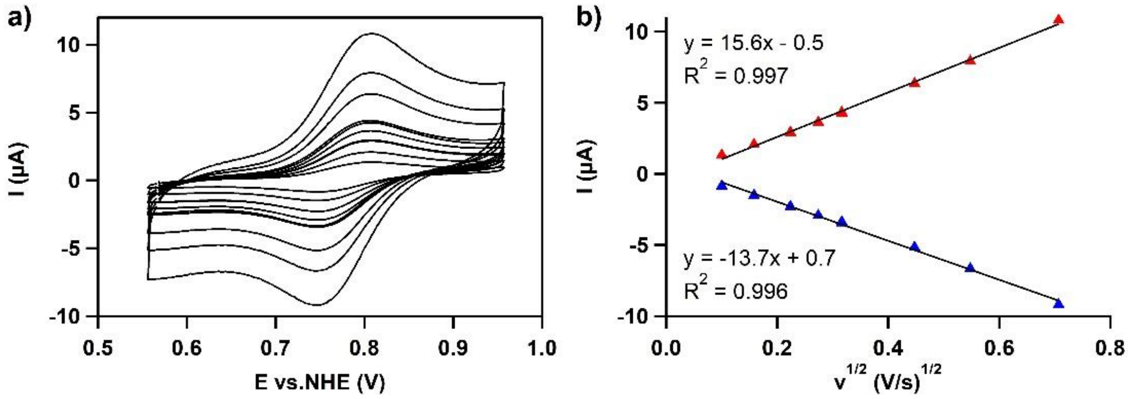

Figure S7. a) CVs of the $Ru^{II/III}$ redox couple of a 0.5 mM *cis*-[Ru^{II}(bpy)$_2$(H_2O)$_2$]$^{2+}$ in 100 mM pH 2.5 phosphate buffer at varying scan rate. GC, Au and RHE were used as WE, CE and RE, respectively. Potentials were converted to NHE. b) Linear correlation between the cathodic (red) and anodic current (blue) peak currents of the $Ru^{II/III}$ redox couple of *cis*-[Ru^{II}(bpy)$_2$(H_2O)$_2$]$^{2+}$.

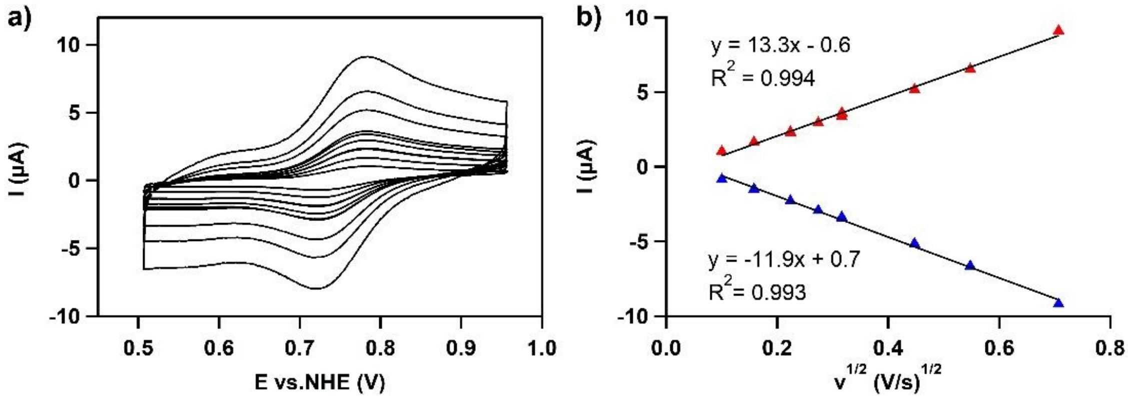

Figure S8. CVs of the $Ru^{II/III}$ redox couple of a 0.5 mM *cis*-[Ru^{II}(4,4'-Me_2-bpy)$_2$(H_2O)$_2$]$^{2+}$ in 100 mM pH 2.5 phosphate buffer at varying scan rate. GC, Au and RHE were used as WE, CE and RE, respectively. Potentials were converted to NHE. b) Linear correlation between the cathodic (red) and anodic current (blue) peak currents of the $Ru^{II/III}$ redox couple of *cis*-[Ru^{II}(4,4'-Me_2-bpy)$_2$(H_2O)$_2$]$^{2+}$.

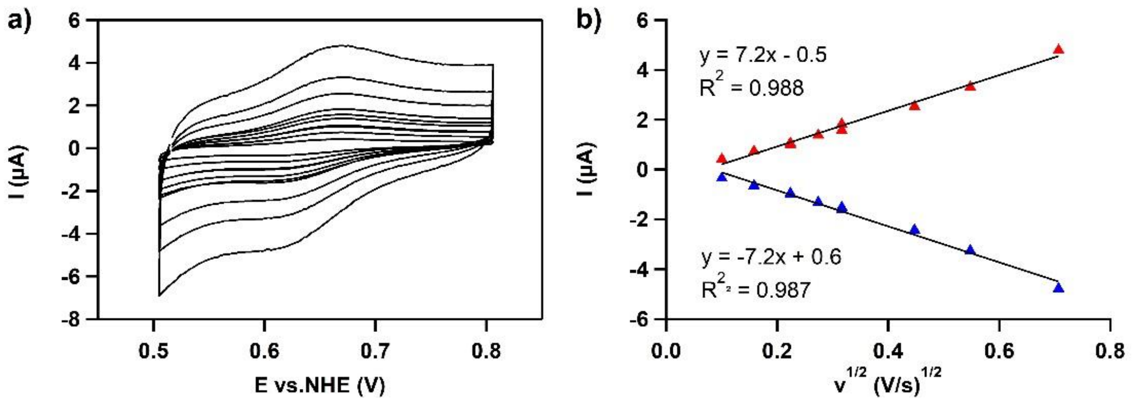

Figure S*9*. CVs of the $Ru^{II/III}$ redox couple of a 0.5 mM *cis*-[Ru^{II}(4,4’-$(OMe)_2$-bpy$)_2(H_2O)_2]^{2+}$ in 100 mM pH 2.5 phosphate buffer at varying scan rate. GC, Au and RHE were used as WE, CE and RE, respectively. Potentials were converted to NHE. b) Linear correlation between the cathodic (red) and anodic current (blue) peak currents of the $Ru^{II/III}$ redox couple of *cis*-[Ru^{II}(4,4’-$(OMe)_2$-bpy$)_2(H_2O)_2]^{2+}$.

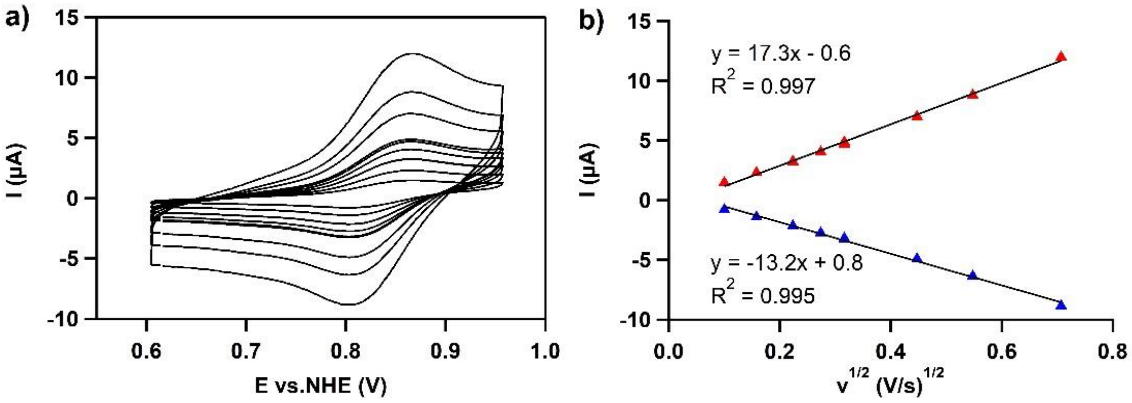

Figure S*10*. CVs of the $Ru^{II/III}$ redox couple of a 0.5 mM *cis*-[Ru^{II}(4,4’-Cl_2-bpy$)_2(H_2O)_2]^{2+}$ in 100 mM pH 2.5 phosphate buffer at varying scan rate. GC, Au and RHE were used as WE, CE and RE, respectively. Potentials were converted to NHE. b) Linear correlation between the cathodic (red) and anodic current (blue) peak currents of the $Ru^{II/III}$ redox couple of *cis*-[Ru^{II}(4,4’-Cl_2-bpy$)_2(H_2O)_2]^{2+}$.

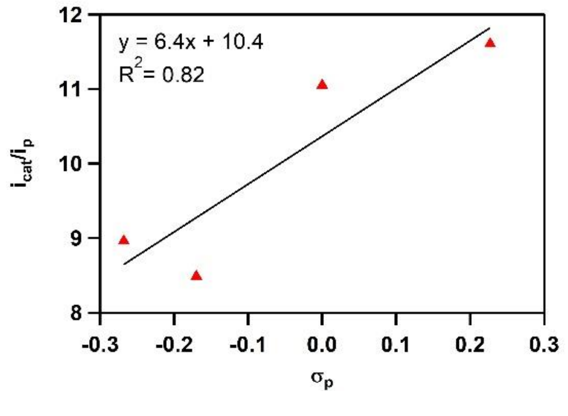

Figure S11. WO activity dependence of i_{cat}/i_p on the Hammett parameter at pH 2.5, where $Ru^{VI}(=O)_2$ is formed via PCET. CVs were recorded in a pH 2.5 phosphate buffer with a 0.5 mM catalyst concentration at a GC WE.

V Stability

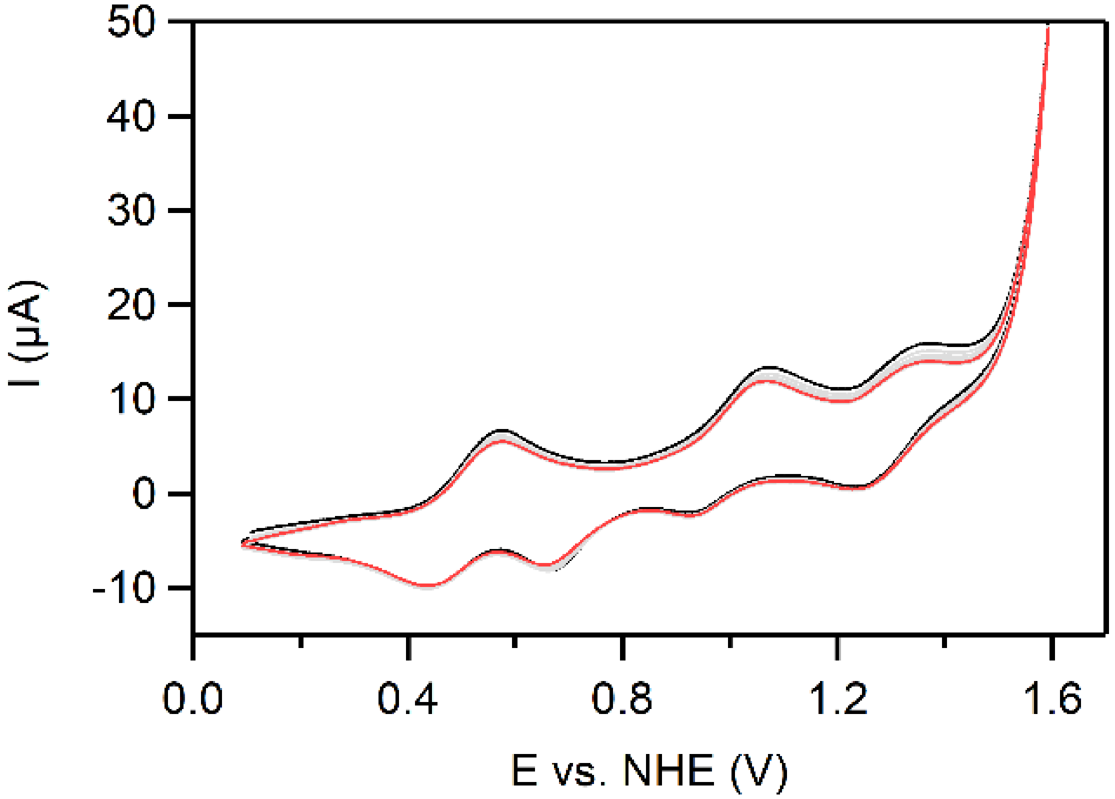

Figure S12. CV of 0.5 mM *cis*-$[Ru^{II}(bpy)_2(H_2O)_2]^{2+}$ in 100 mM pH 7 phosphate buffer at a scan rate of 100 mV/s. BDD, Au and RHE were used as WE, CE and RE, respectively. Potentials were converted to normal hydrogen electrode (NHE). Black = scan 2, red = scan 10

VI. NMR spectra

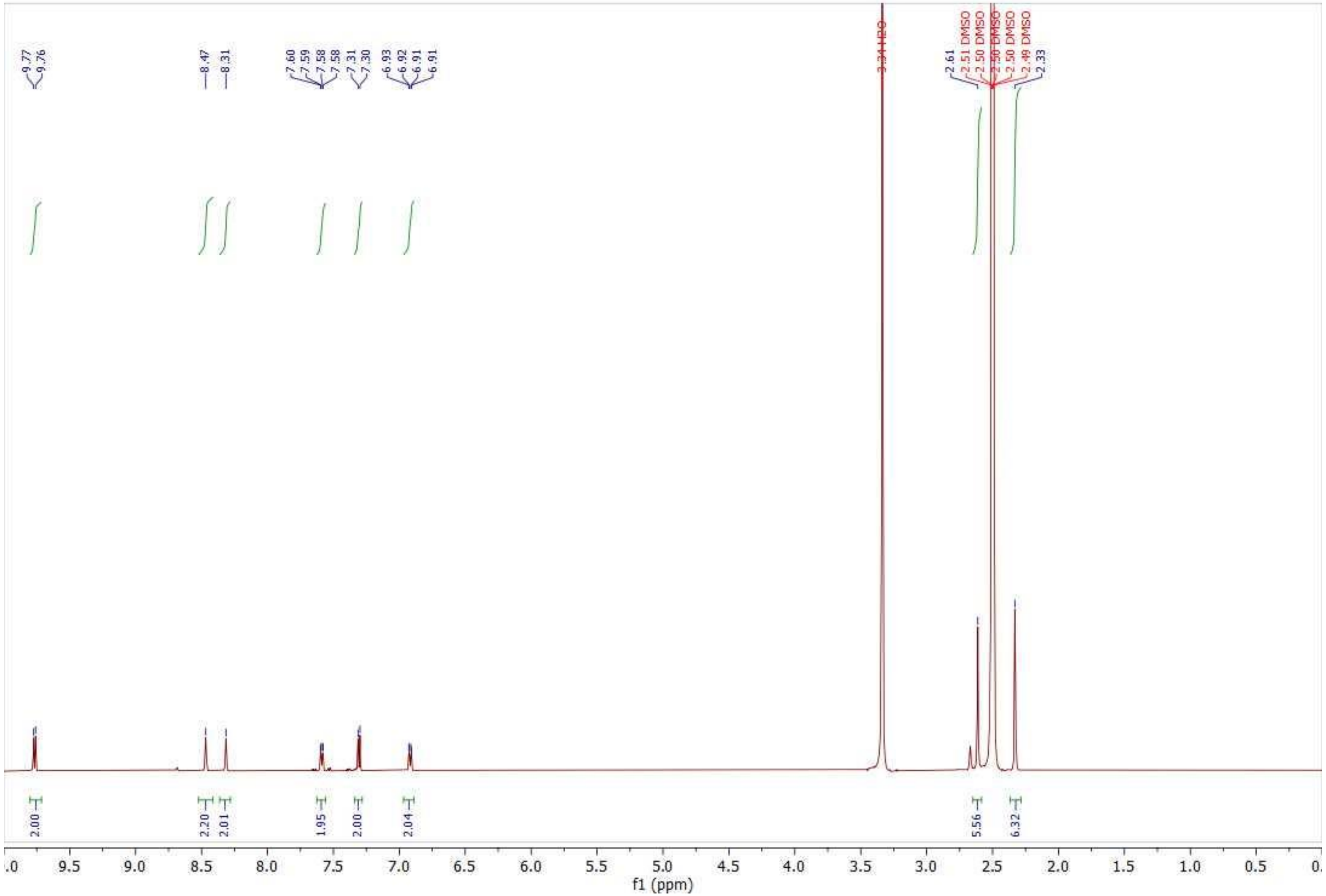

Figure S13. ^{1}H NMR – *cis*-dichlorido-bis(4,4'-dimethyl-2,2'-bipyridine)ruthenium(II) – DMSO-d_6 400 MHz

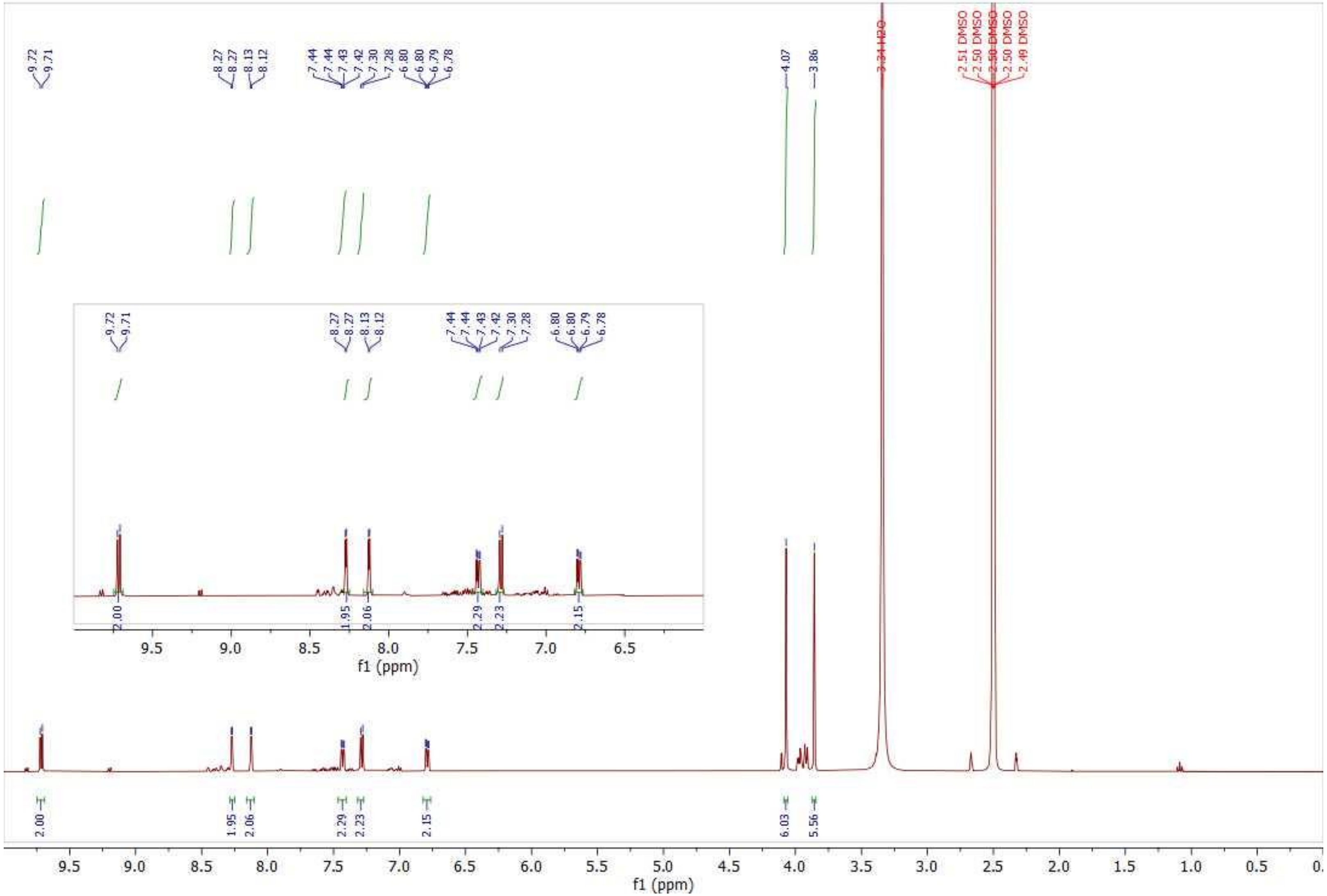

Figure S14. ^{1}H NMR – *cis*-dichlorido-bis(4,4'-dimethoxy-2,2'-bipyridine)ruthenium(II) – DMSO-d_6 400 MHz

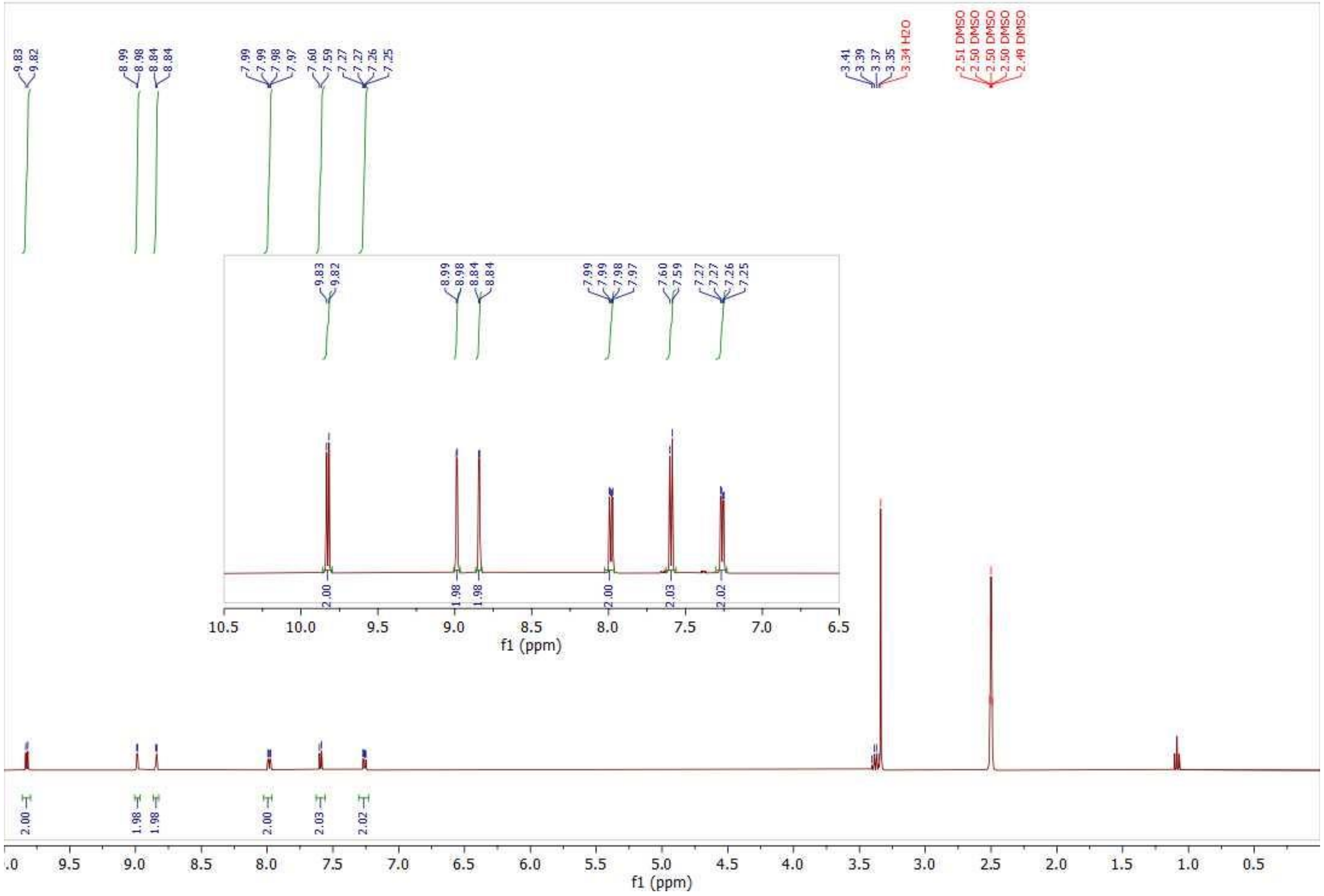

Figure S15: ^{1}H NMR – *cis*-dichlorido-bis(4,4’-dichloro-2,2’-bipyridine)ruthenium(II) – DMSO-d_6 400 MHz

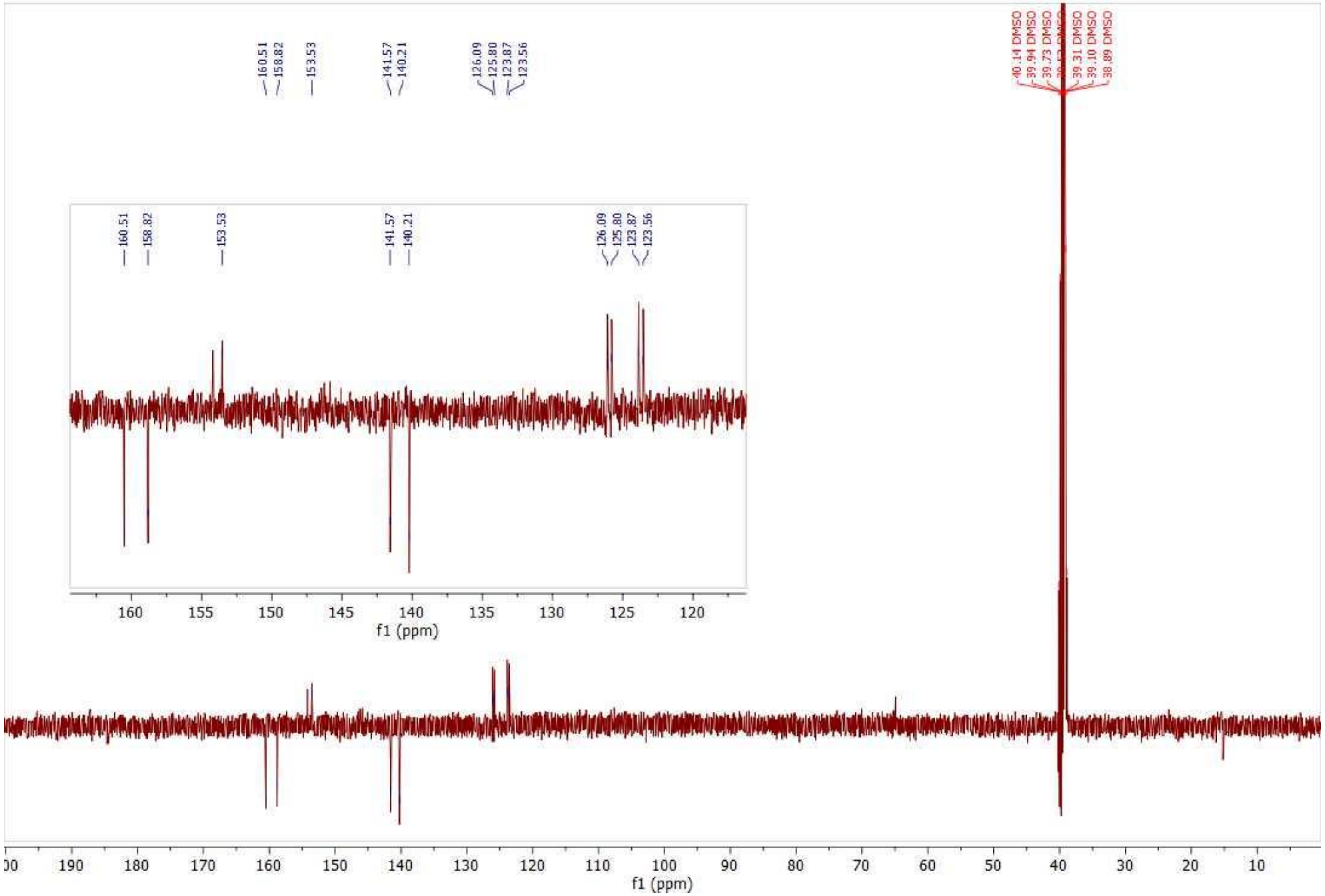

Figure S16: ^{13}C APT NMR – *cis*-dichlorido-bis(4,4'-dichloro-2,2'-bipyridine)ruthenium(II) – DMSO-d_6 101 MHz

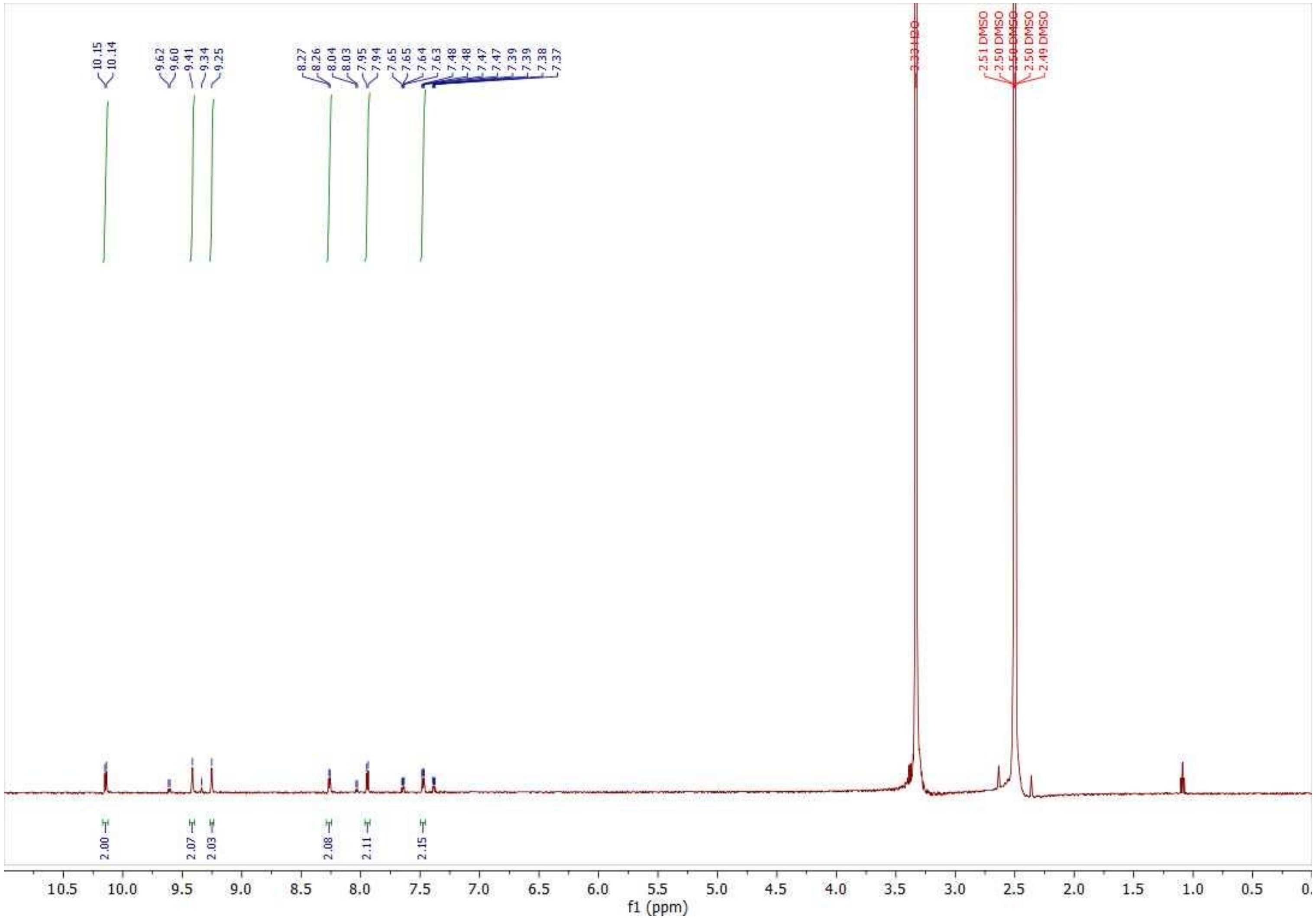

Figure S17: ^{1}H NMR – *cis*-dichlorido-bis(4,4'-bis(trifluormethyl)-2,2'-bipyridine)ruthenium(II) – DMSO-d$_6$ 500 MHz

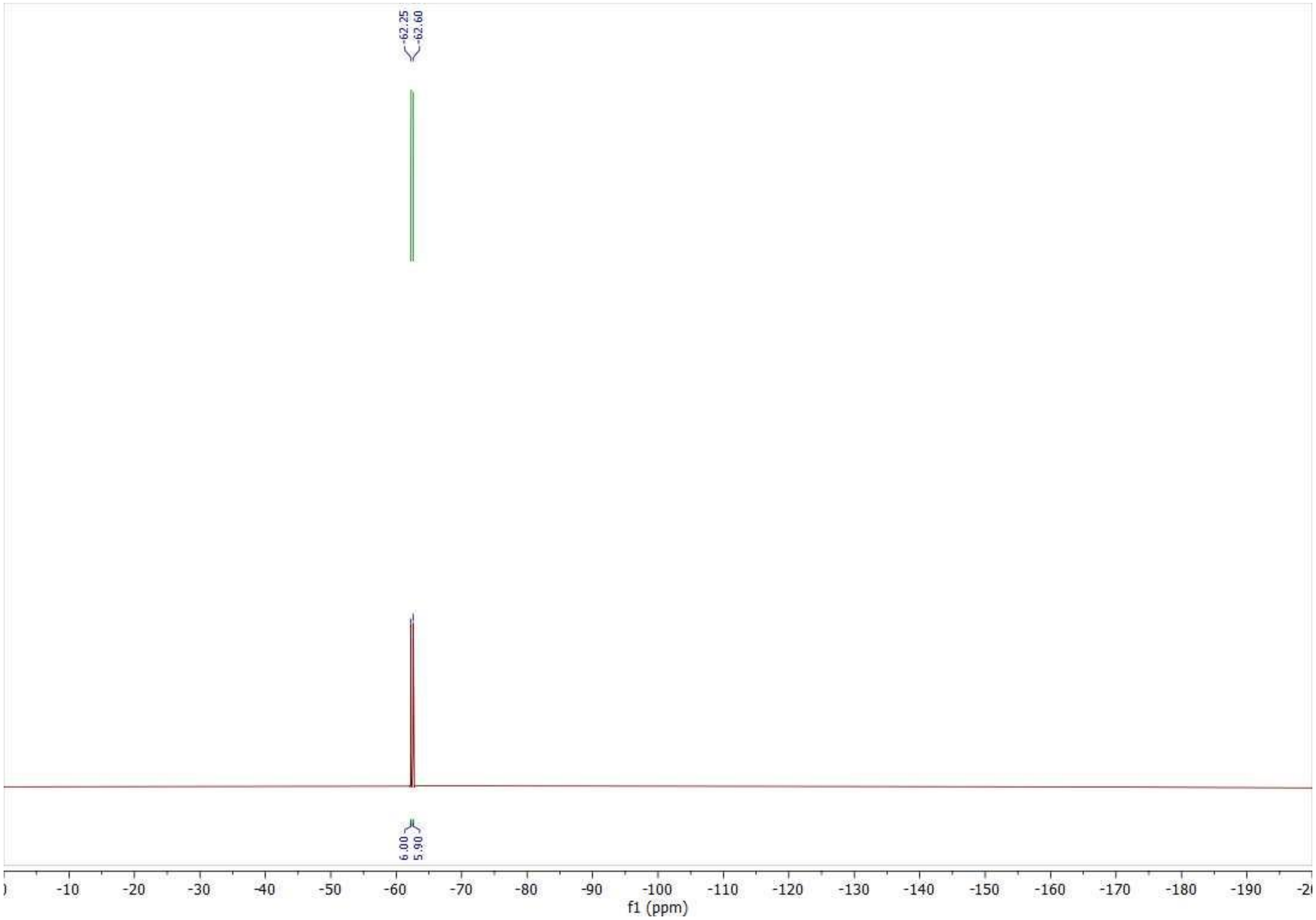

Figure S18: ^{19}F NMR – *cis*-dichlorido-bis(4,4'-bis(trifluormethyl)-2,2'-bipyridine)ruthenium(II) – DMSO-d_6 471 MHz

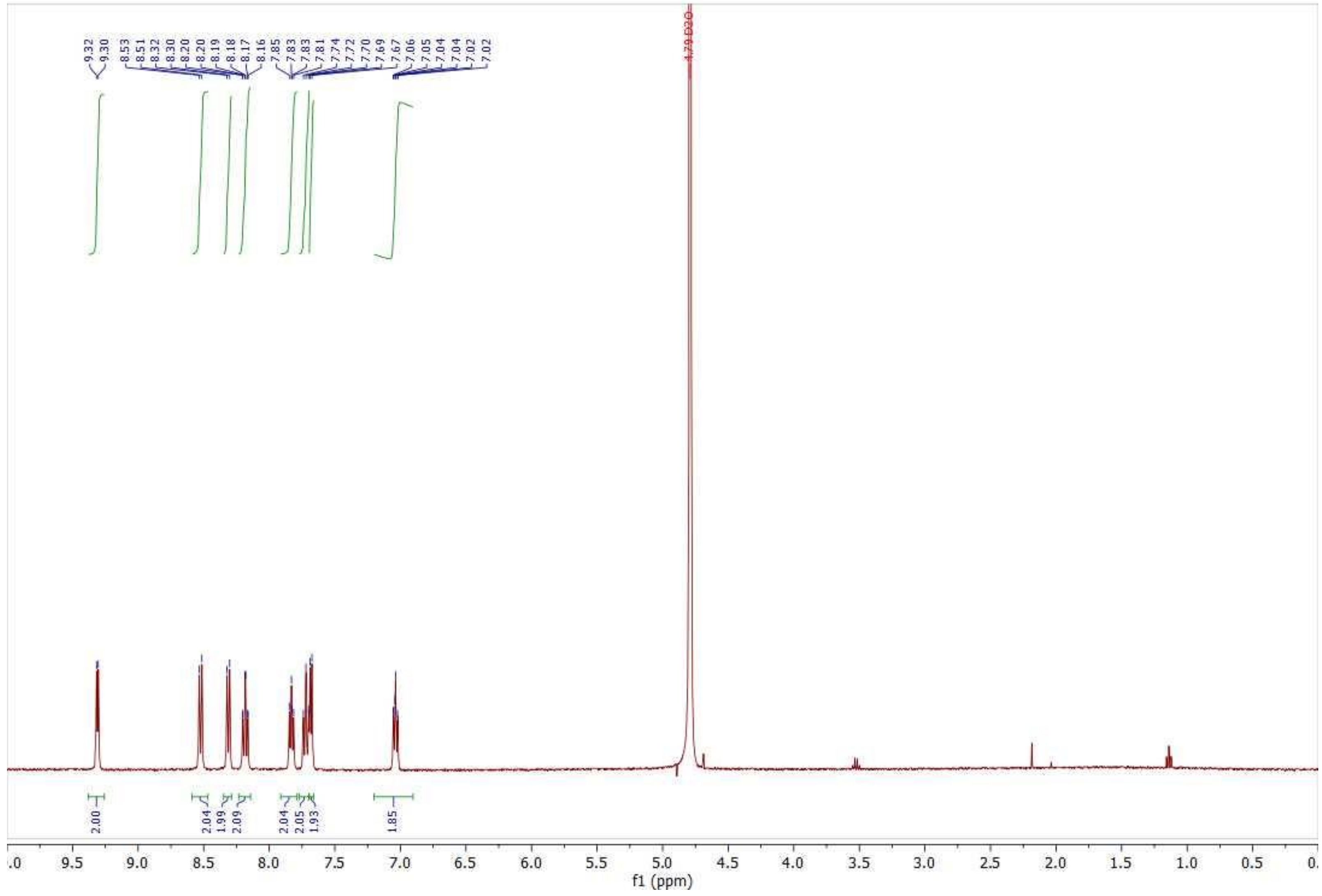

Figure S19: ^{1}H NMR – *cis*-bis(2,2'-bipyridine)bis(trifluoromethanesulfonato)ruthenium(II) – D_2O 400 MHz

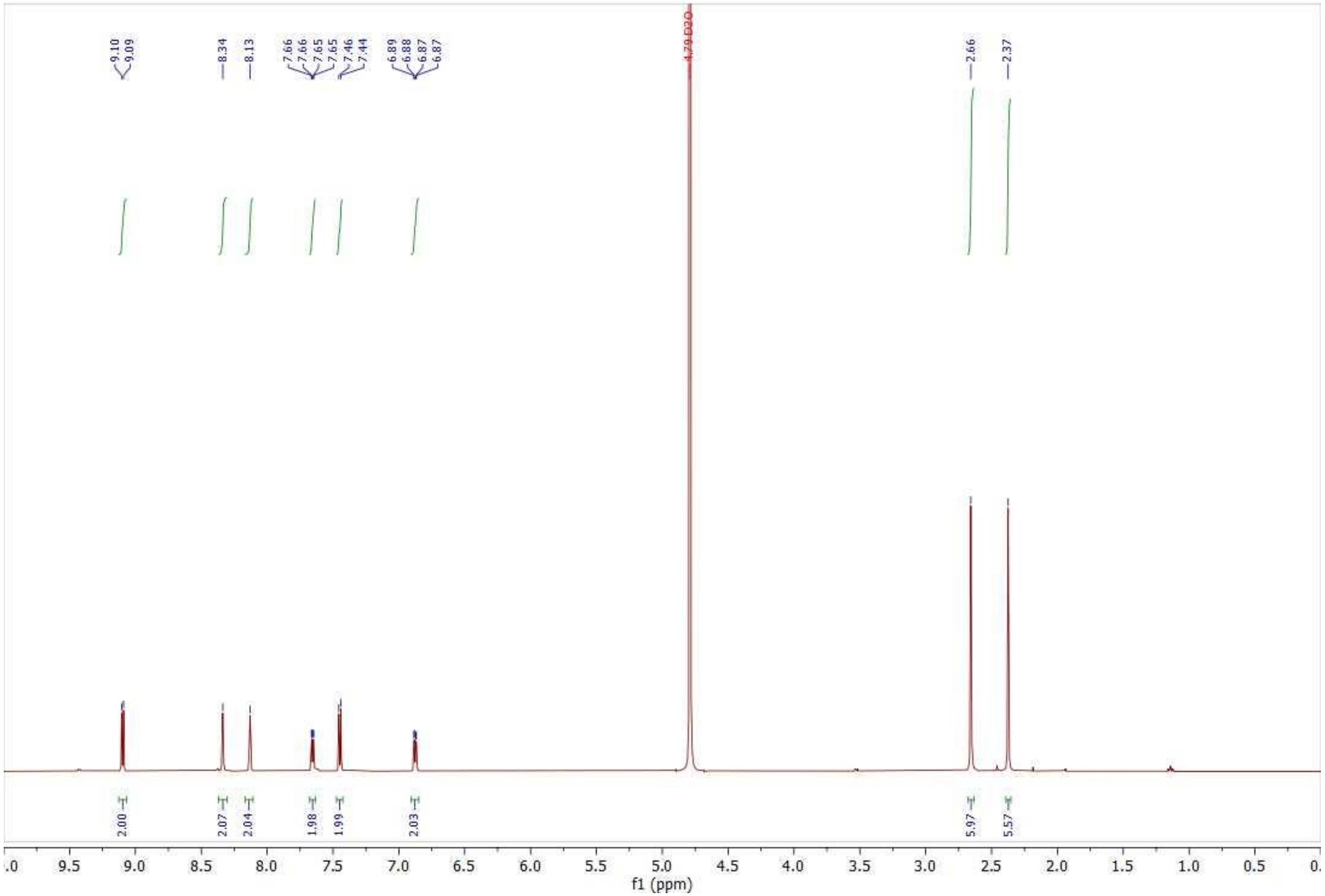

Figure S20: ^{1}H NMR – *cis*-bis(4,4’-dimethyl-2,2’-bipyridine)bis(trifluoromethanesulfonato)ruthenium(II) – D_2O 400 MHz

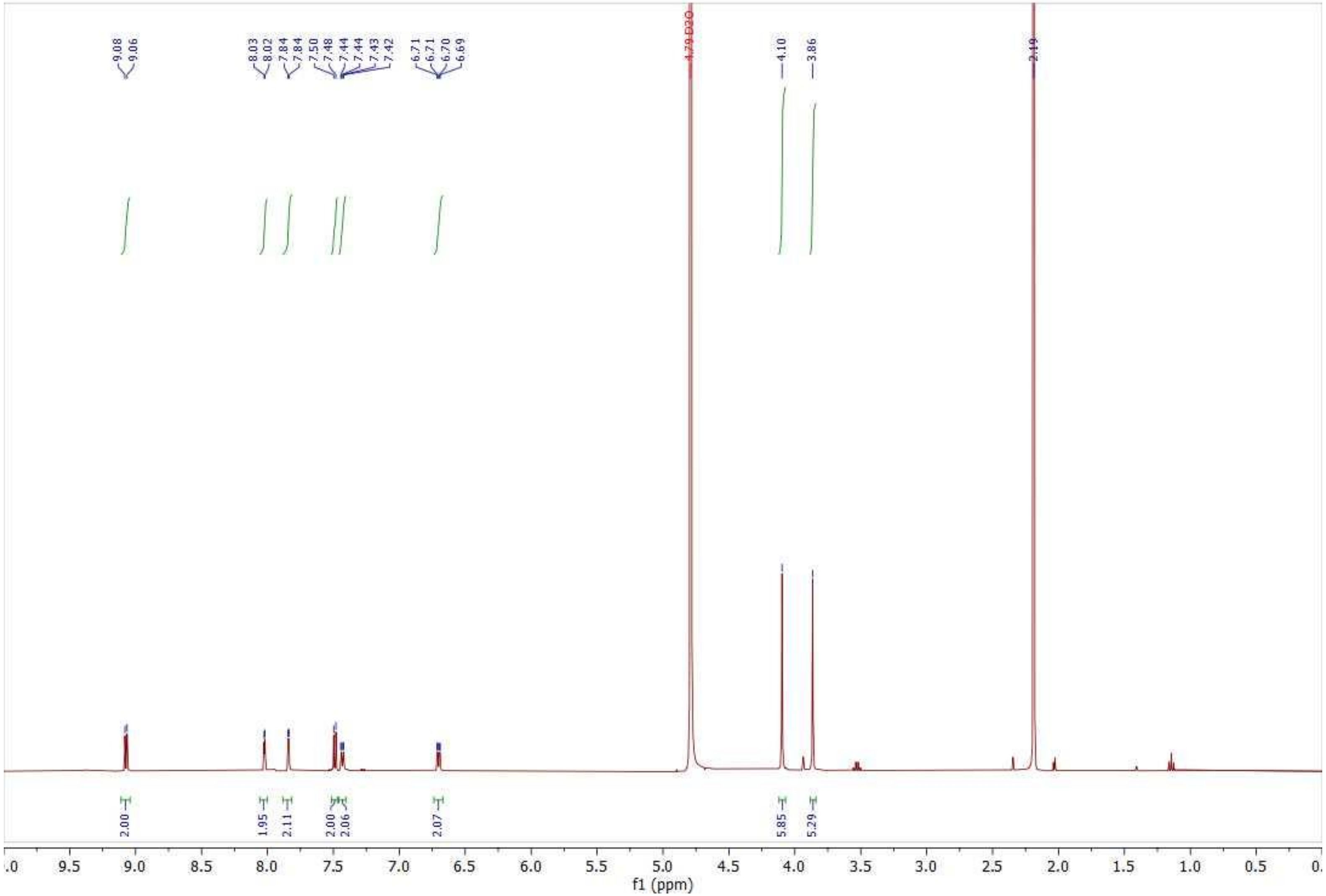

Figure S21: ^{1}H NMR – *Cis*-bis(4,4'-dimethoxy-2,2'-bipyridine)bis(trifluoromethanesulfonato)ruthenium(II) – D_2O 400 MHz

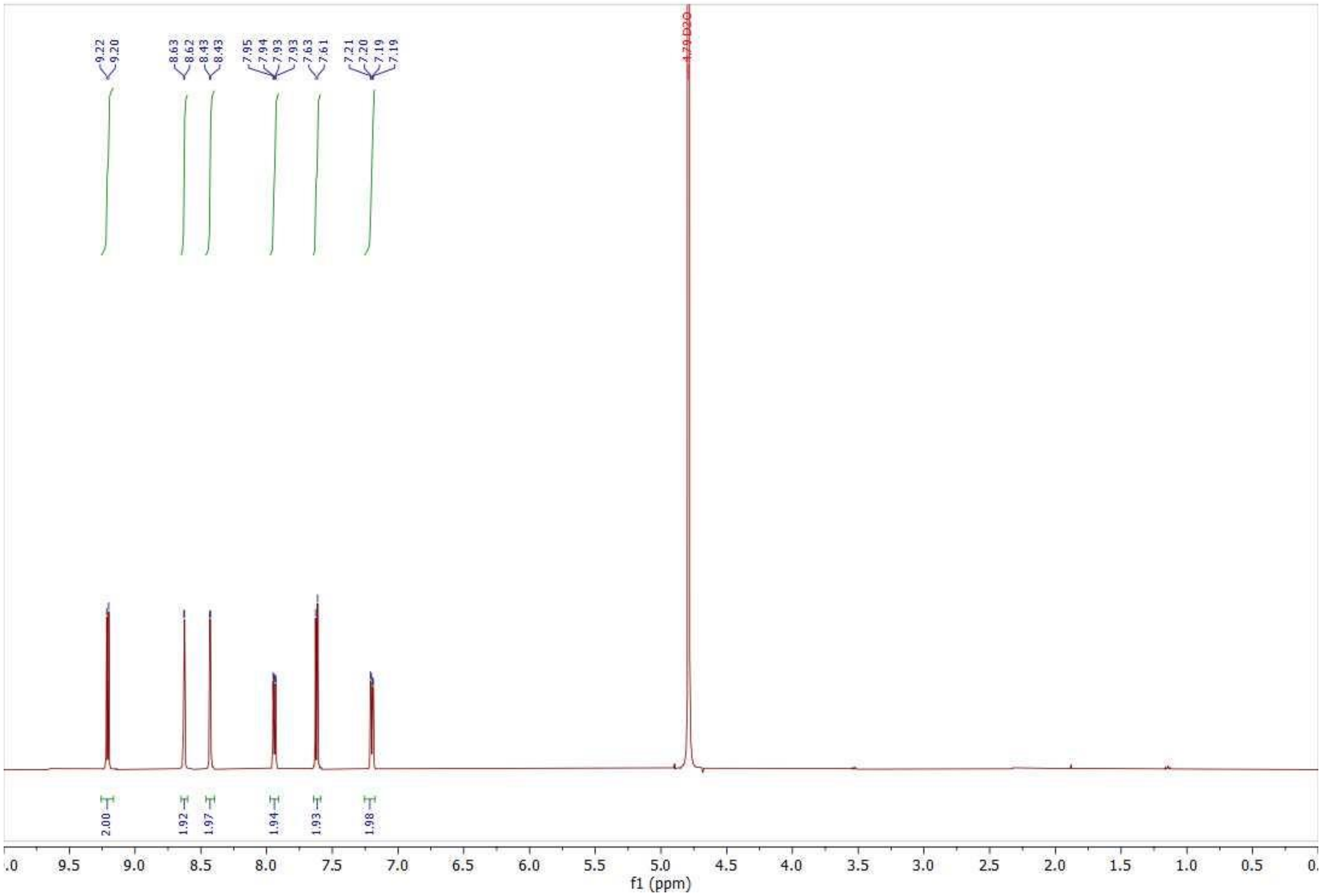

Figure S22: ^{1}H NMR – *cis*-bis(4,4’-dichloro-2,2’-bipyridine)bis(trifluoromethanesulfonato)ruthenium(II) – D_2O 400 MHz

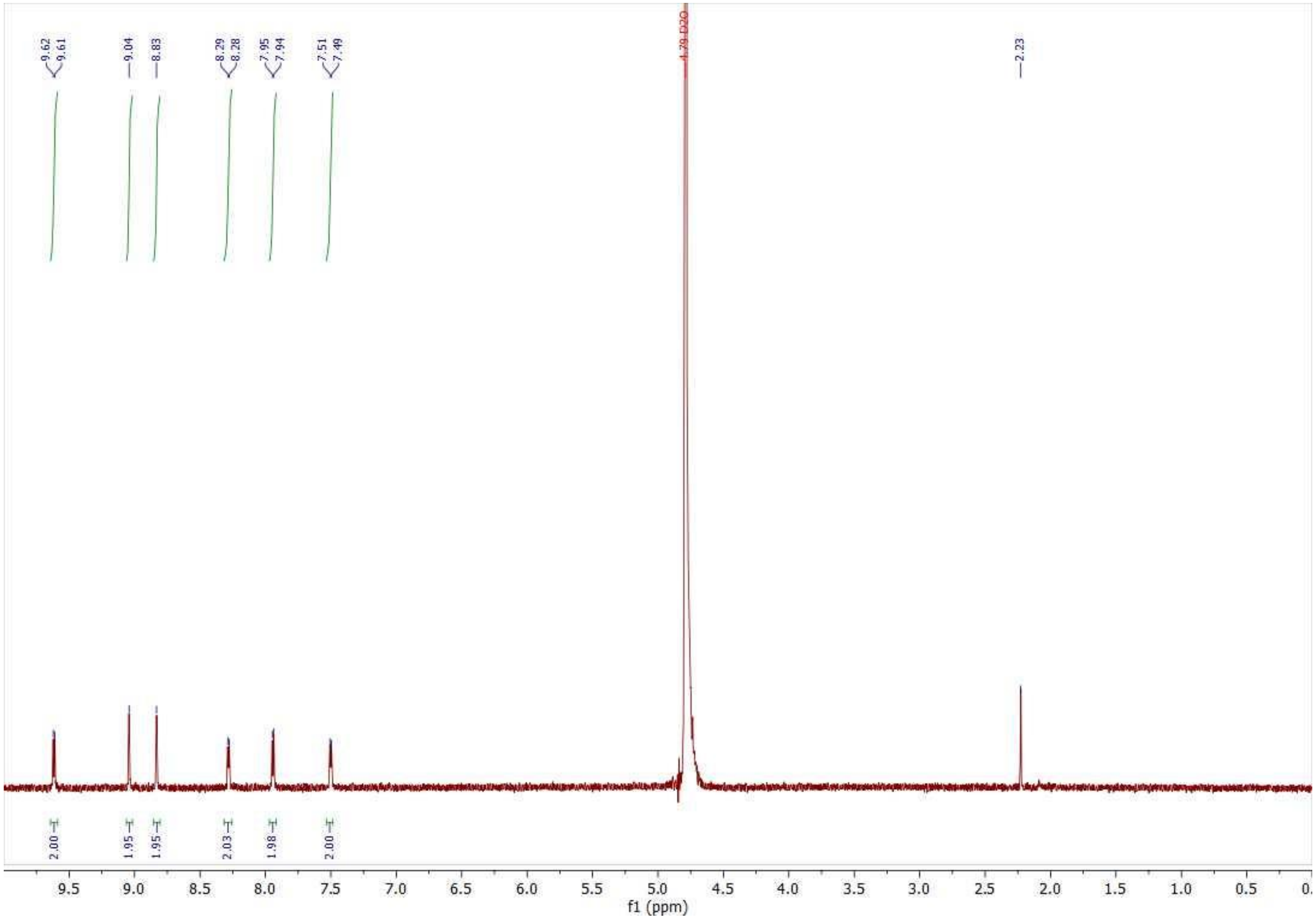

Figure 23: ^{1}H NMR – *cis*-bis(4,4'-bis(trifluormethyl)-2,2'-bipyridine)bis(trifluoromethanesulfonato)ruthenium(II) – D_2O 500 MHz

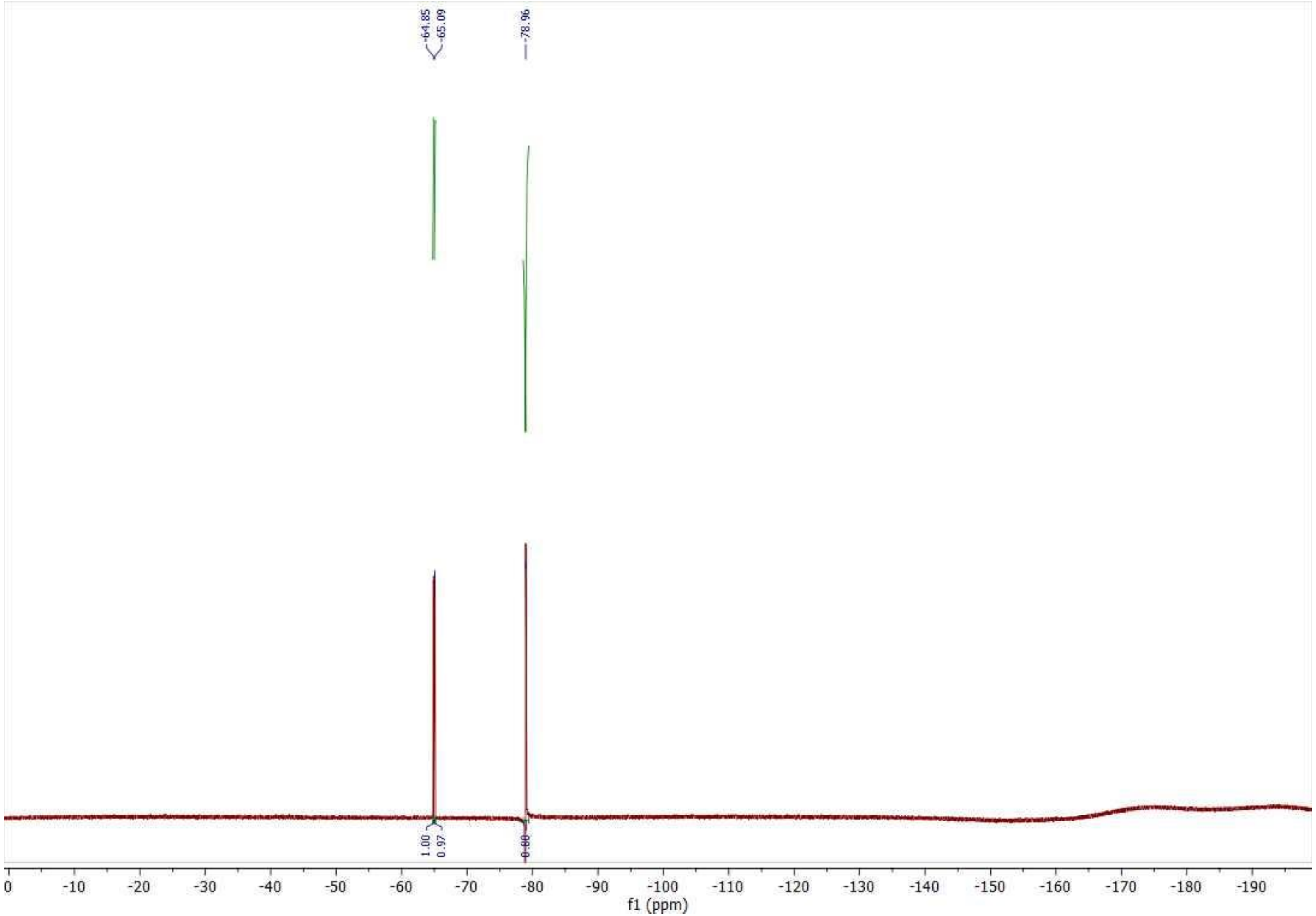

Figure S24: ^{19}F NMR – *cis*-bis(4,4'-bis(trifluormethyl)-2,2'-bipyridine)bis(trifluoromethanesulfonato)ruthenium(II) – D_2O 471 MHz

VI. DPV data used to construct Pourbaix Diagrams

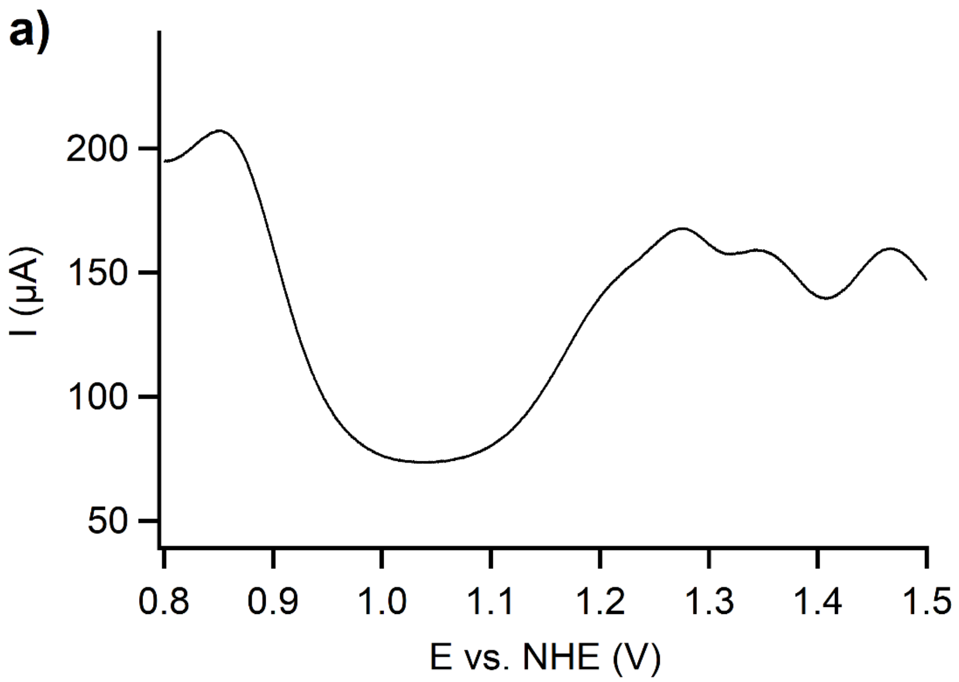

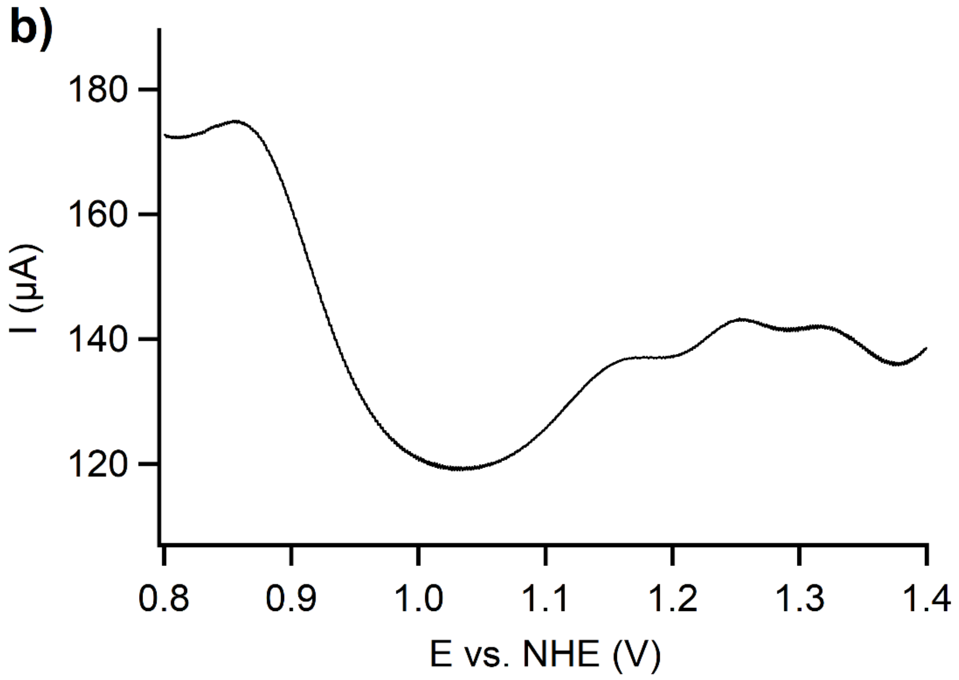

c)

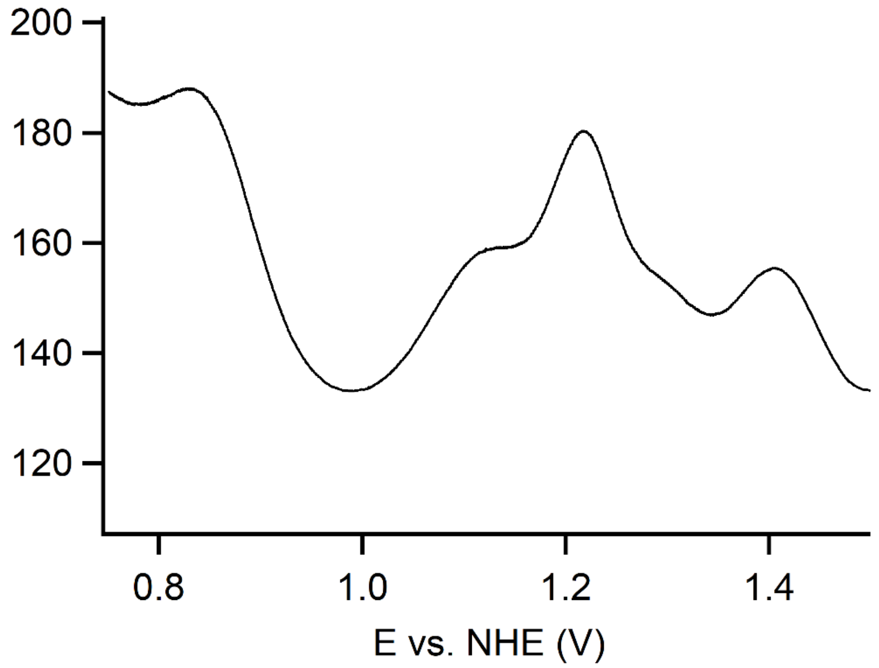
200
180
160
140
120
I (μA)
0.8
1.0
1.2
1.4
E vs. NHE (V)

d)

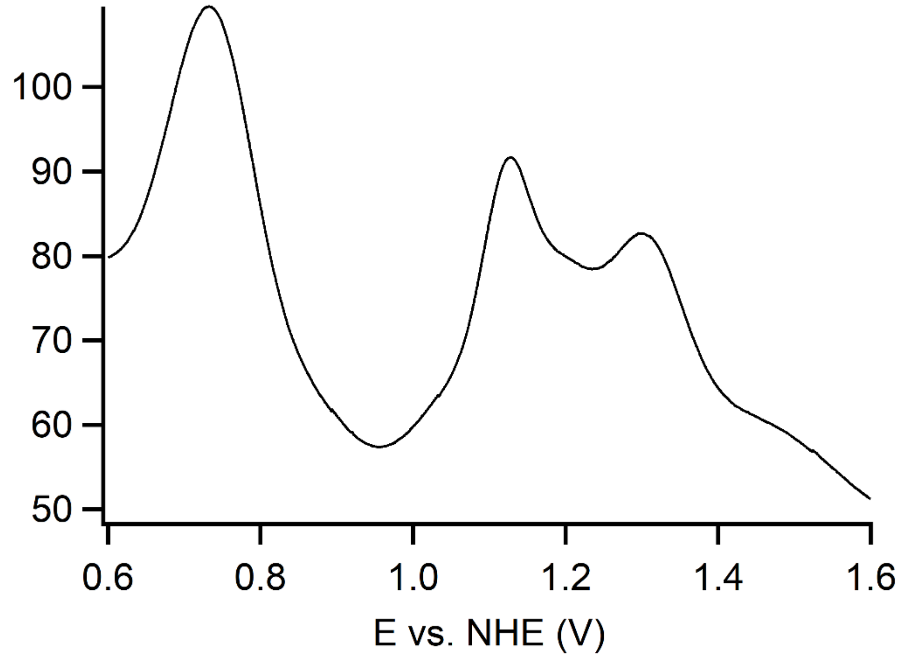
100
90
80
70
60
50
I (μA)
0.6
0.8
1.0
1.2
1.4
1.6
E vs. NHE (V)

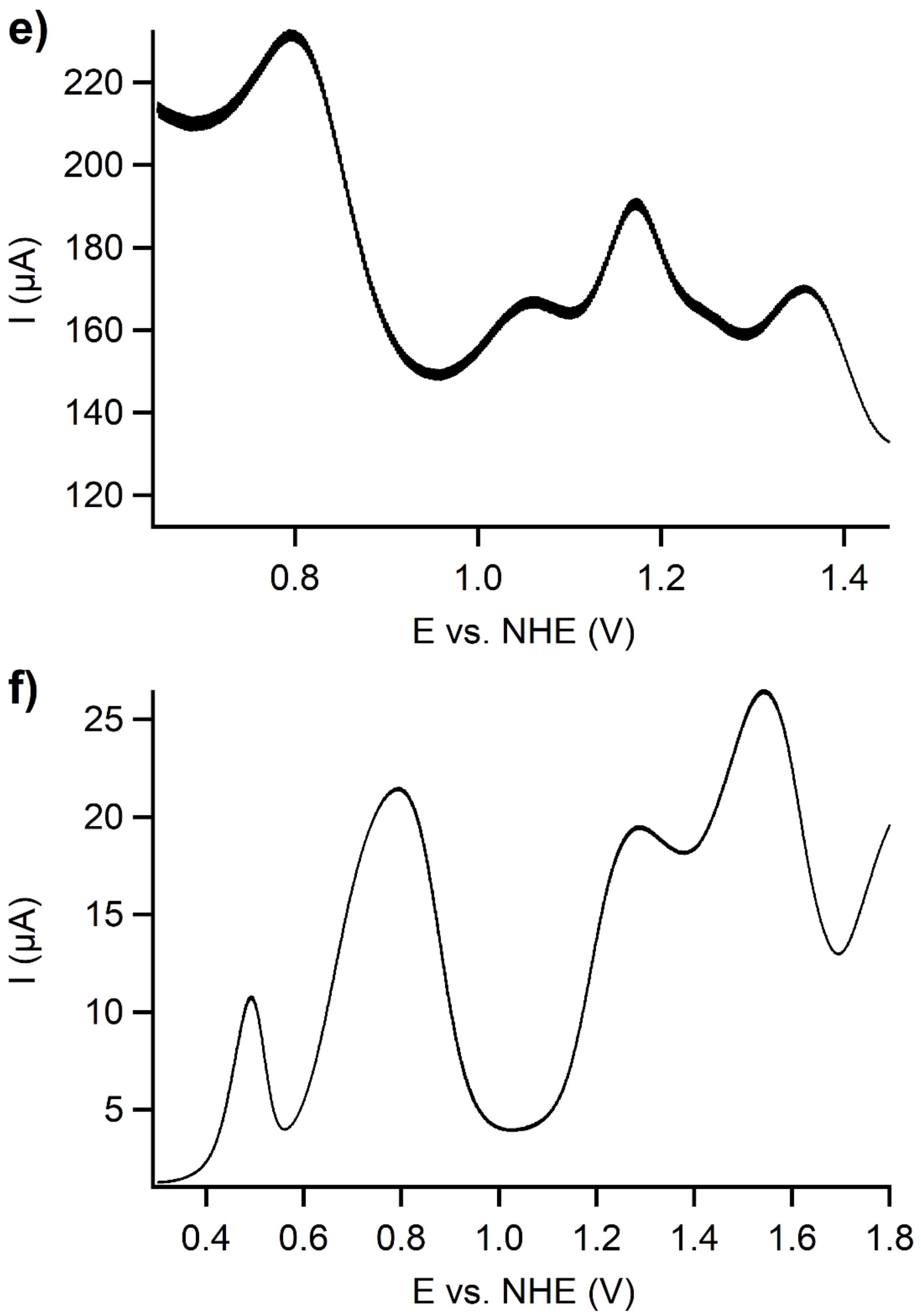
e)
220
200
180
160
140
120
I (µA)
0.8
1.0
1.2
1.4
E vs. NHE (V)
f)
25
20
15
10
5
I (µA)
0.4
0.6
0.8
1.0
1.2
1.4
1.6
1.8
E vs. NHE (V)

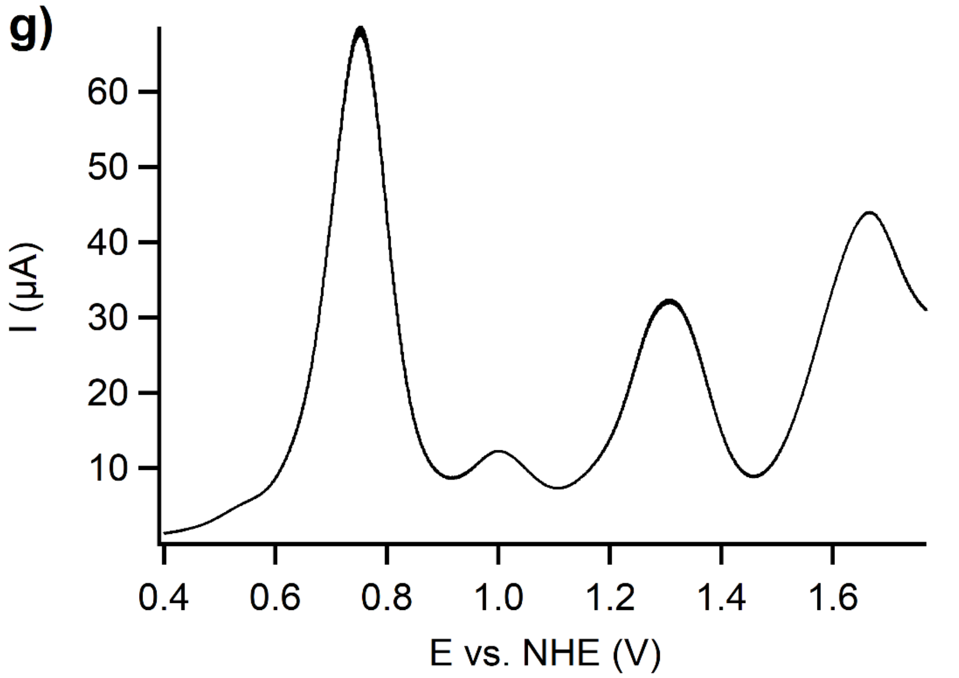
g)
60
50
40
30
20
10
I (μA)
0.4
0.6
0.8
1.0
1.2
1.4
1.6
E vs. NHE (V)

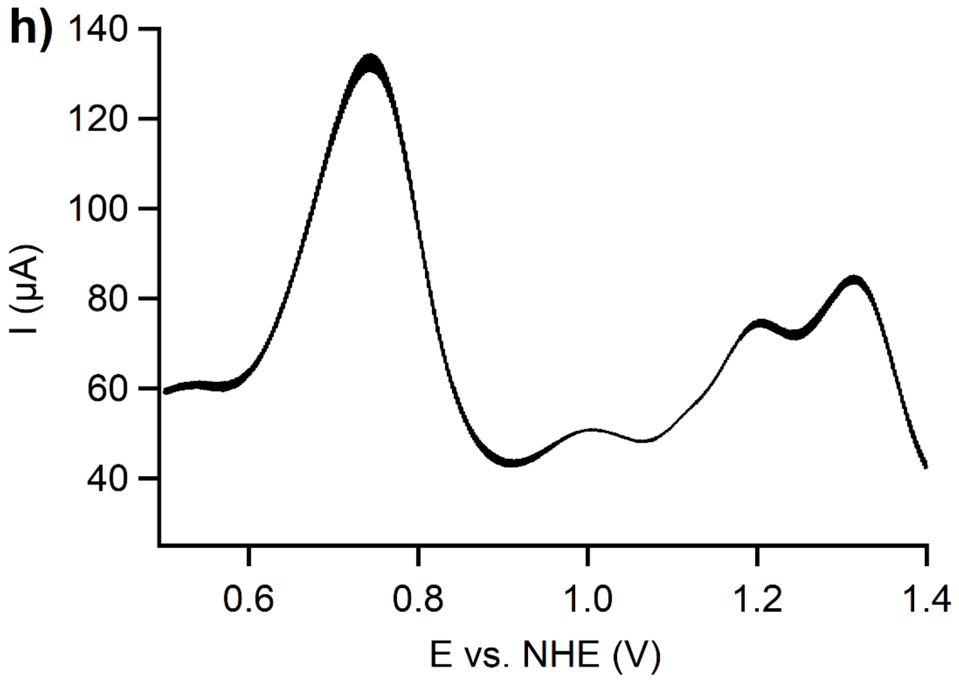
h)
140
120
100
80
60
40
I (μA)
0.6
0.8
1.0
1.2
1.4
E vs. NHE (V)

i)

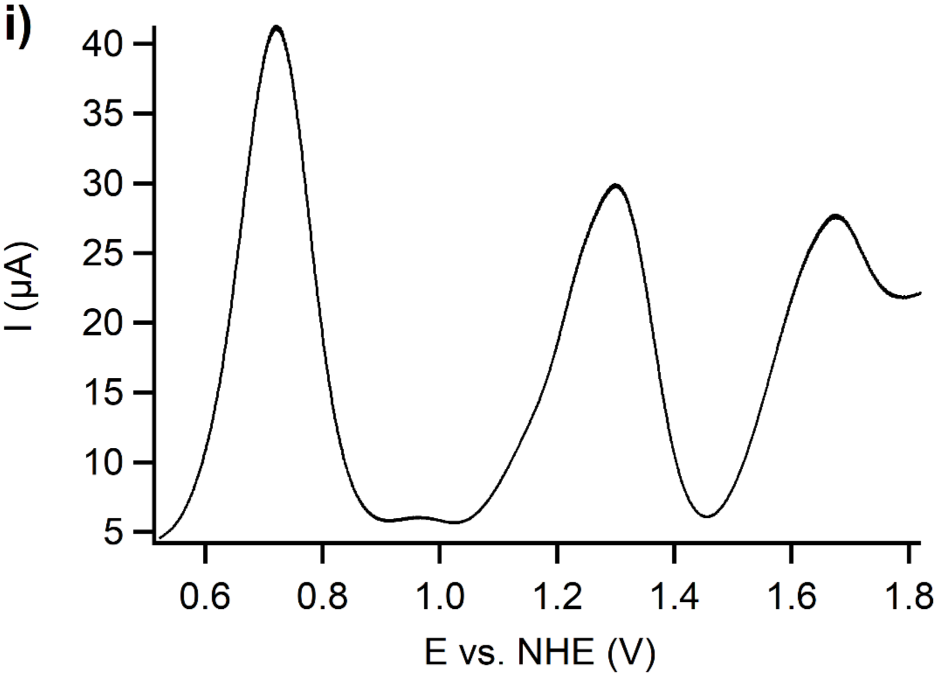

I (μA)
40
35
30
25
20
15
10
5
0.6
0.8
1.0
1.2
1.4
1.6
1.8
E vs. NHE (V)

j)

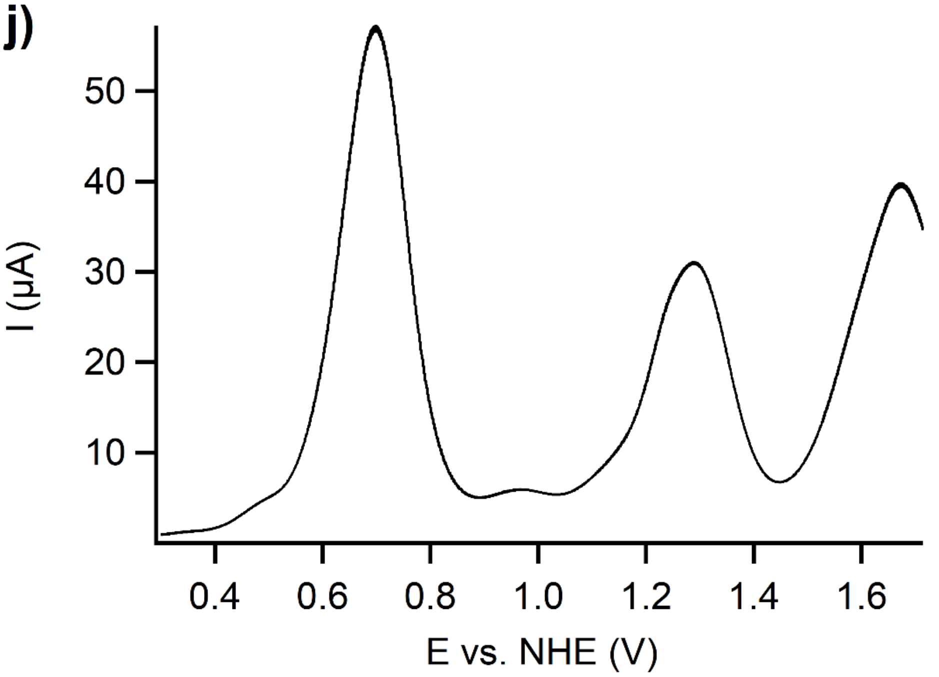

I (μA)
50
40
30
20
10
0.4
0.6
0.8
1.0
1.2
1.4
1.6
E vs. NHE (V)

k)

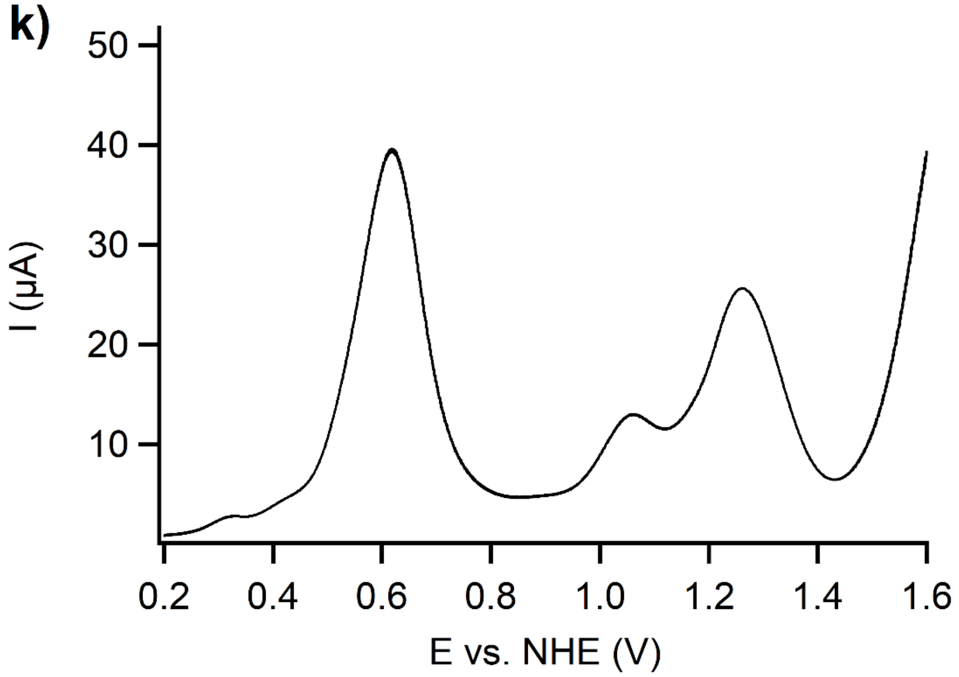

I (µA)
50
40
30
20
10
0.2
0.4
0.6
0.8
1.0
1.2
1.4
1.6
E vs. NHE (V)

l)

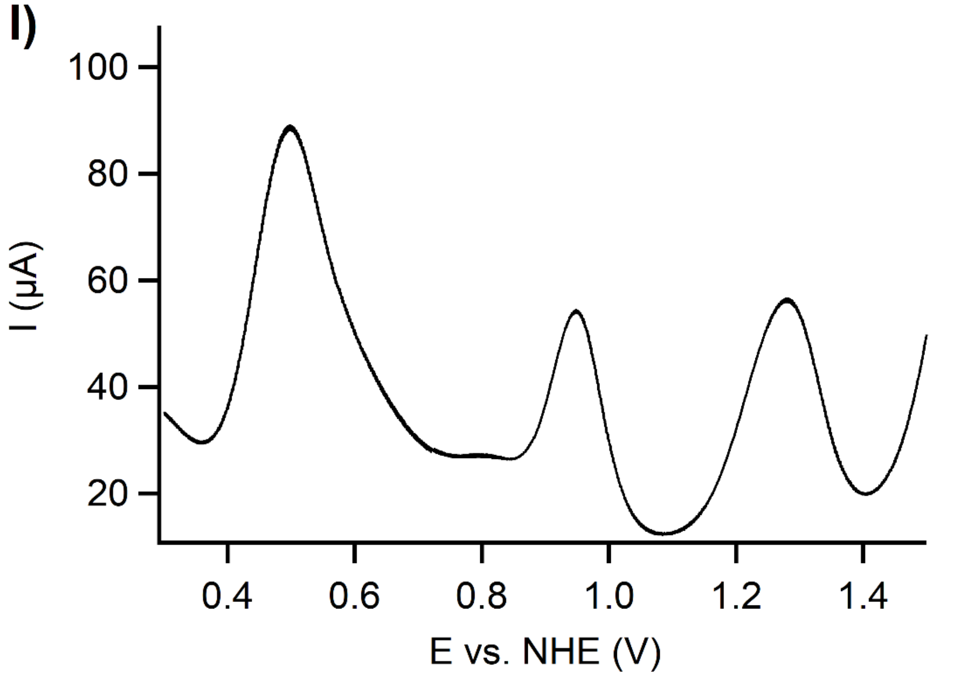

I (µA)
100
80
60
40
20
0.4
0.6
0.8
1.0
1.2
1.4
E vs. NHE (V)

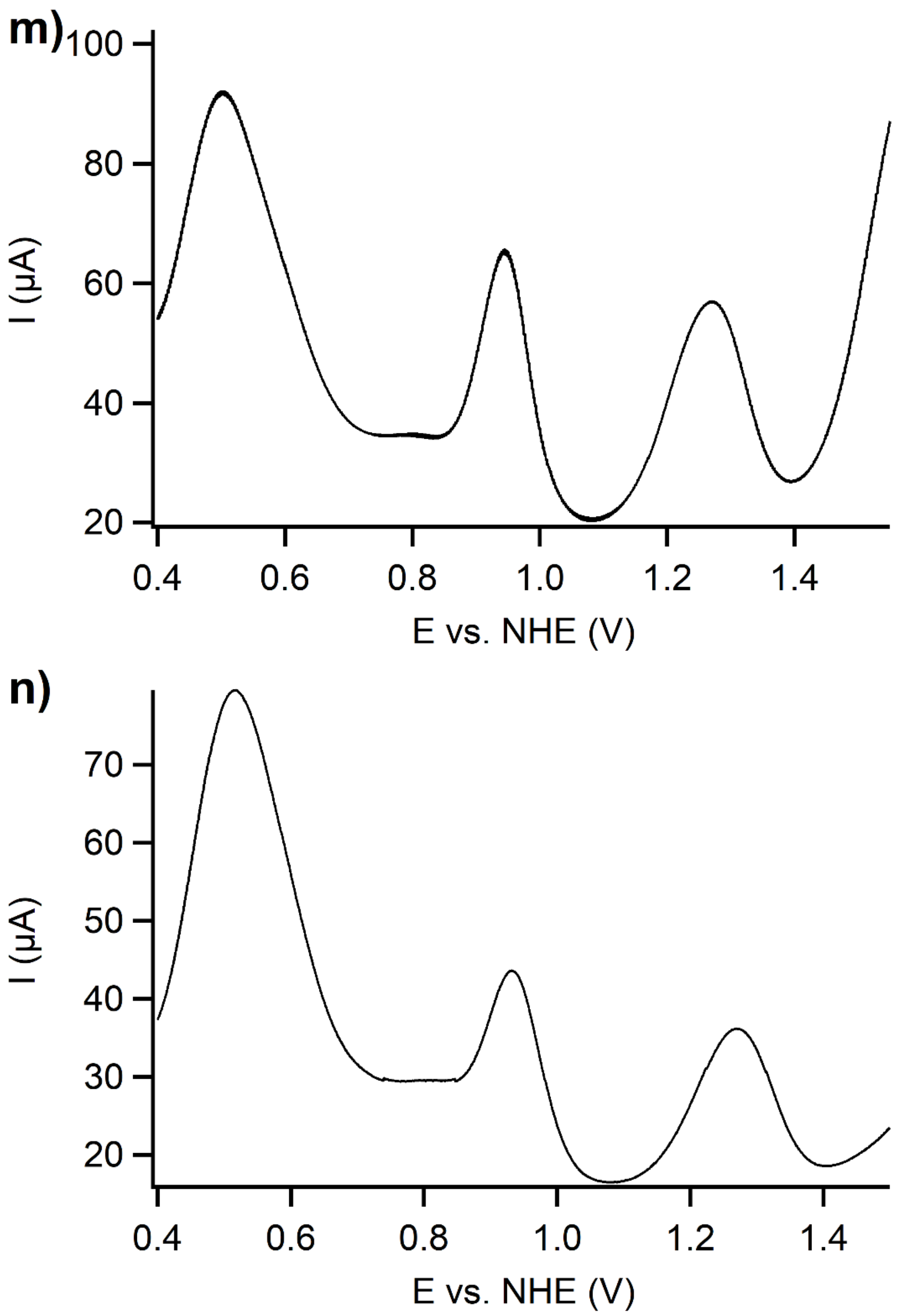
m)
100
80
60
40
20
I (µA)
0.4
0.6
0.8
1.0
1.2
1.4
E vs. NHE (V)
n)
70
60
50
40
30
20
I (µA)
0.4
0.6
0.8
1.0
1.2
1.4
E vs. NHE (V)

o)

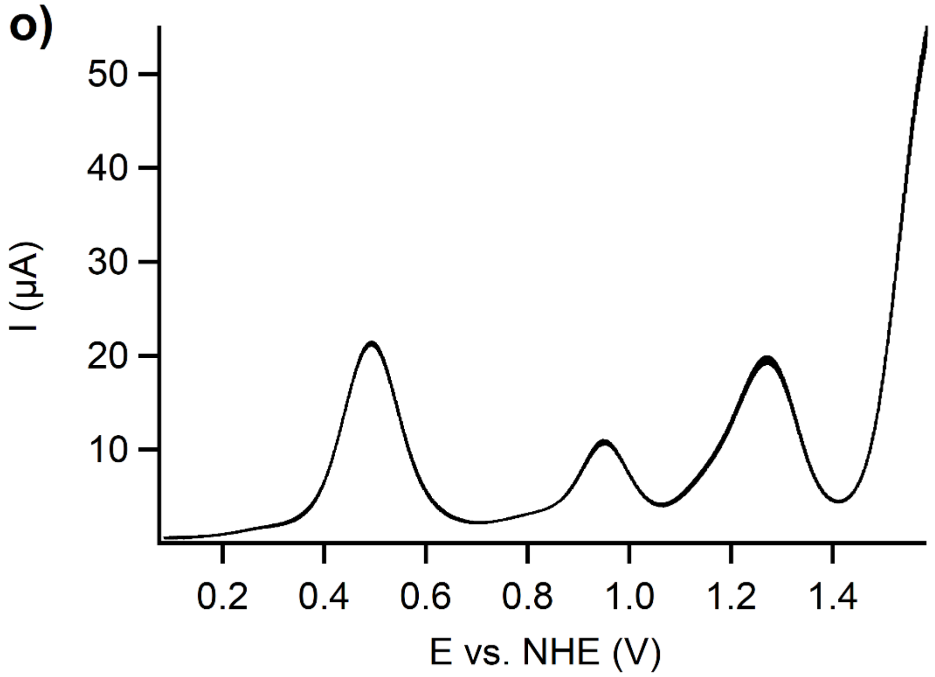
50
40
30
20
10
I (µA)
0.2
0.4
0.6
0.8
1.0
1.2
1.4
E vs. NHE (V)

p)

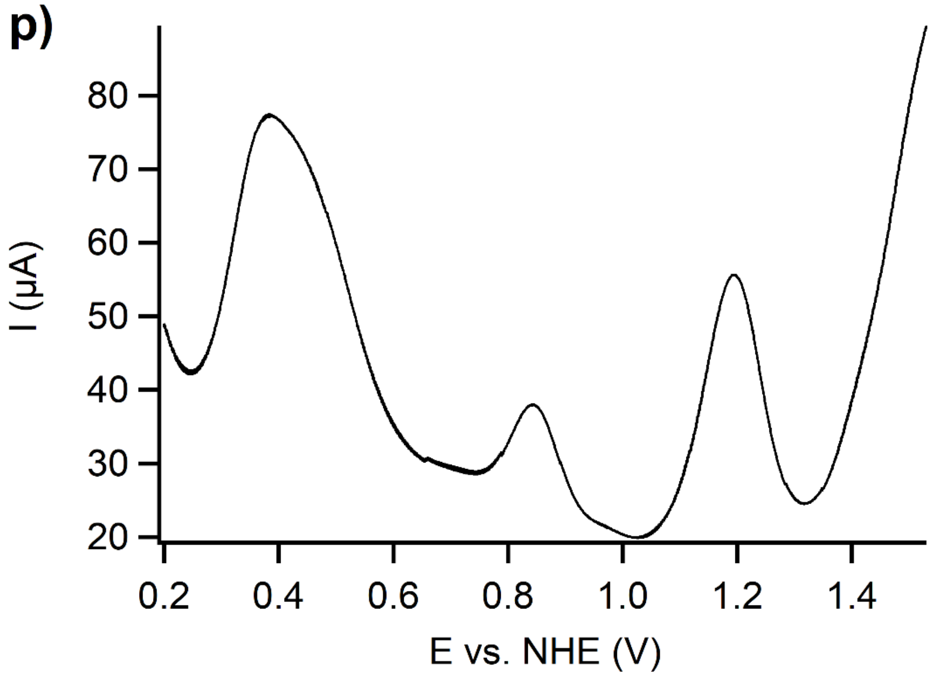
80
70
60
50
40
30
20
I (µA)
0.2
0.4
0.6
0.8
1.0
1.2
1.4
E vs. NHE (V)

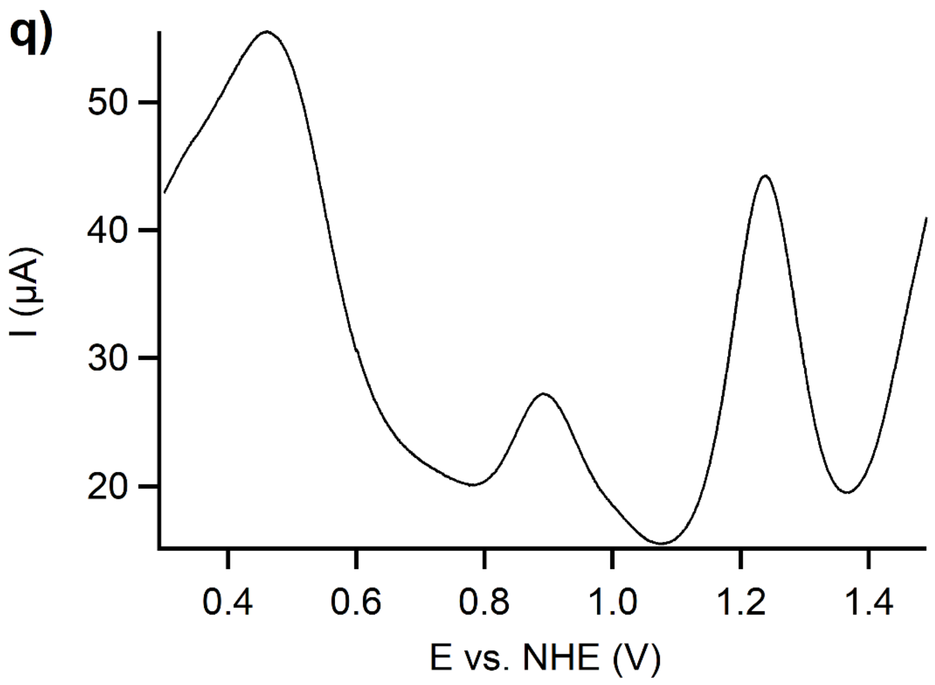

Figure S25. DPV data of *cis*-[Ru^{II}(4,4'-R_2-bpy)$_2$(H_2O)$_2$](OTf)$_2$ (**R=H**) recorded in a 100 mM phosphate solution with the following pH values and electrodes: a) 0.972 GC b) 1.613 GC c) 2.179 GC d) 2.436 GC e) 3.000 GC f) 3.371 BDD g) 3.921 BDD h) 4.726 BDD i) 4.829 BDD j) 4.829 BDD k) 5.849 BDD l) 6.955 GC m) 6.977 GC n) 6.999 GC o) 7.025 BDD p) 7.942 GC q) 8.623 GC. Figures d) and o) have been measured with a 0.5 mM catalyst concentration, all others at a 1.0 mM catalyst concentration.

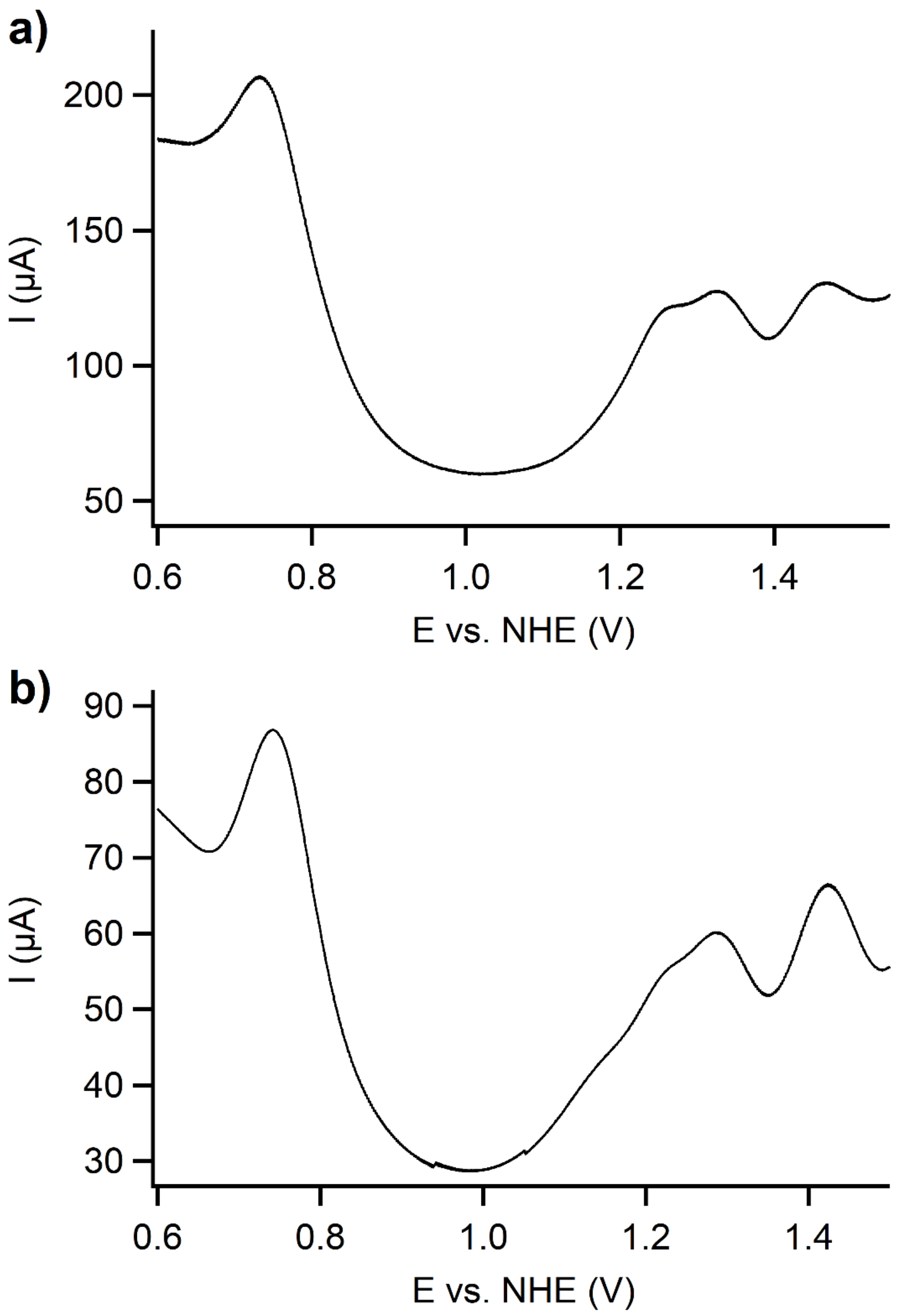
a)
200
150
100
50
I (μA)
0.6
0.8
1.0
1.2
1.4
E vs. NHE (V)
b)
90
80
70
60
50
40
30
I (μA)
0.6
0.8
1.0
1.2
1.4
E vs. NHE (V)

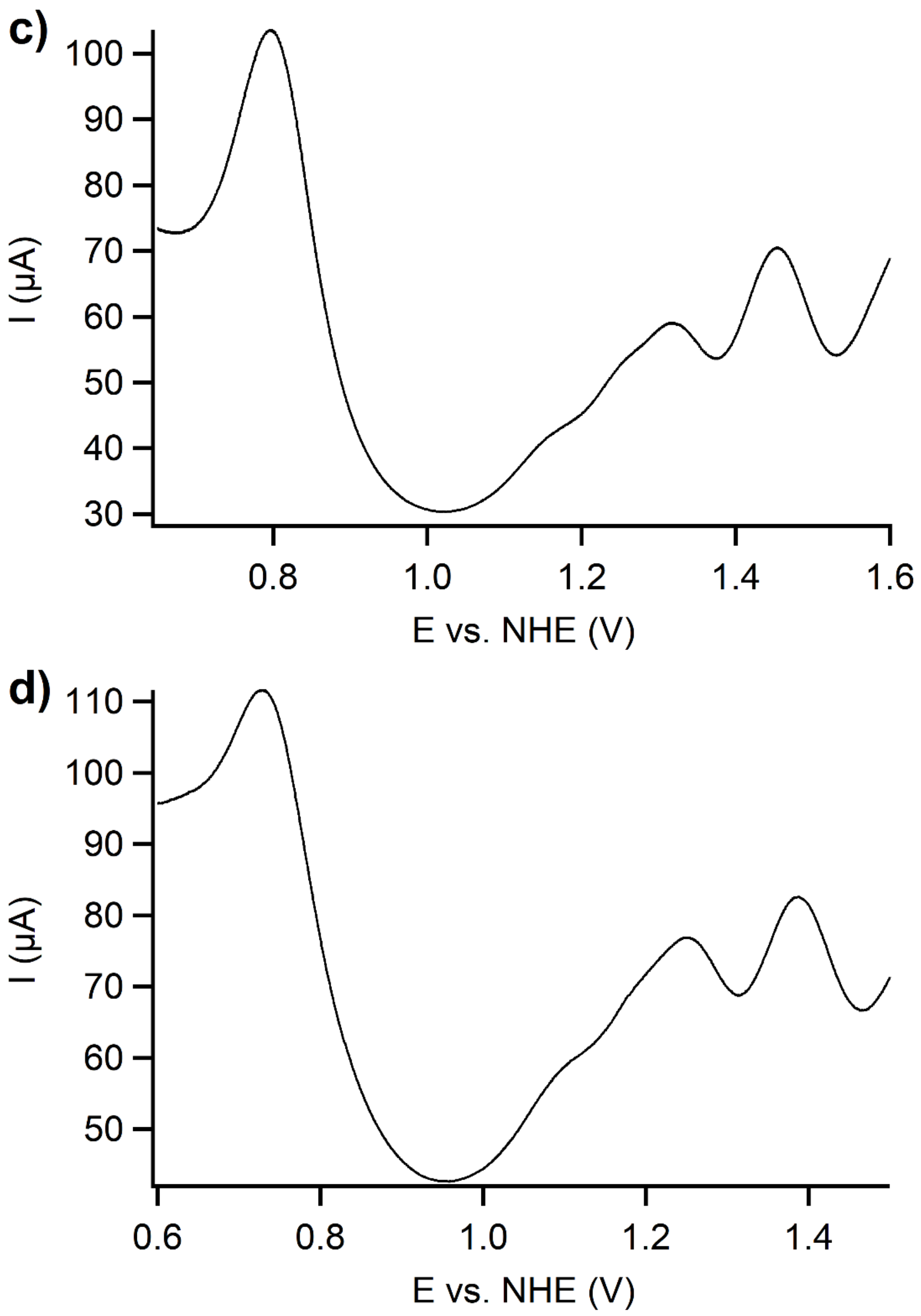
c)
100
90
80
70
60
50
40
30
I (µA)
0.8
1.0
1.2
1.4
1.6
E vs. NHE (V)
d)
110
100
90
80
70
60
50
I (µA)
0.6
0.8
1.0
1.2
1.4
E vs. NHE (V)

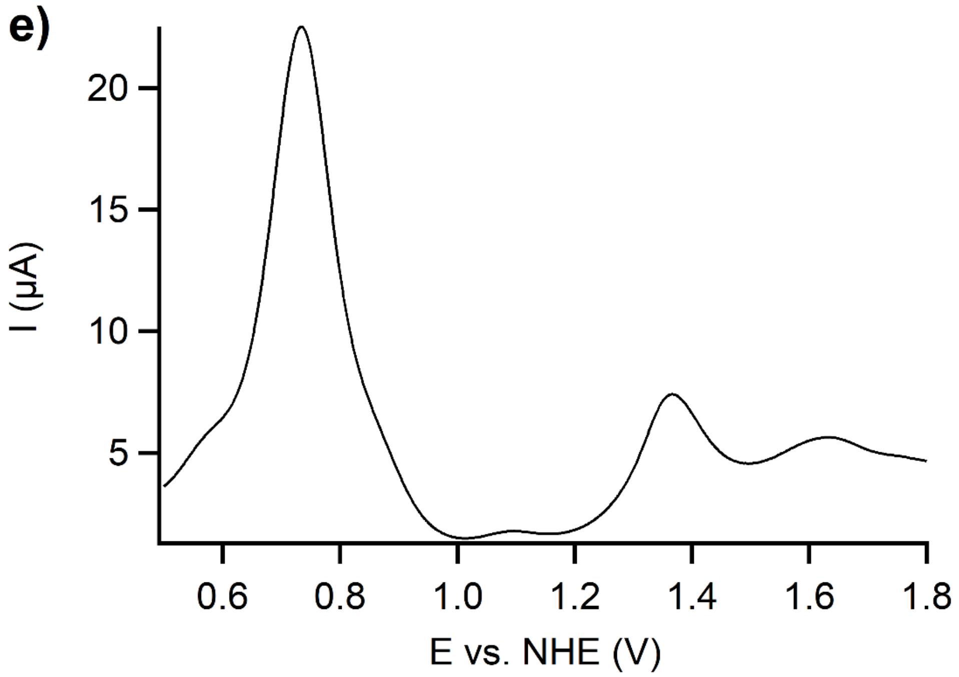
e)
20
15
10
5
I (μA)
0.6
0.8
1.0
1.2
1.4
1.6
1.8
E vs. NHE (V)

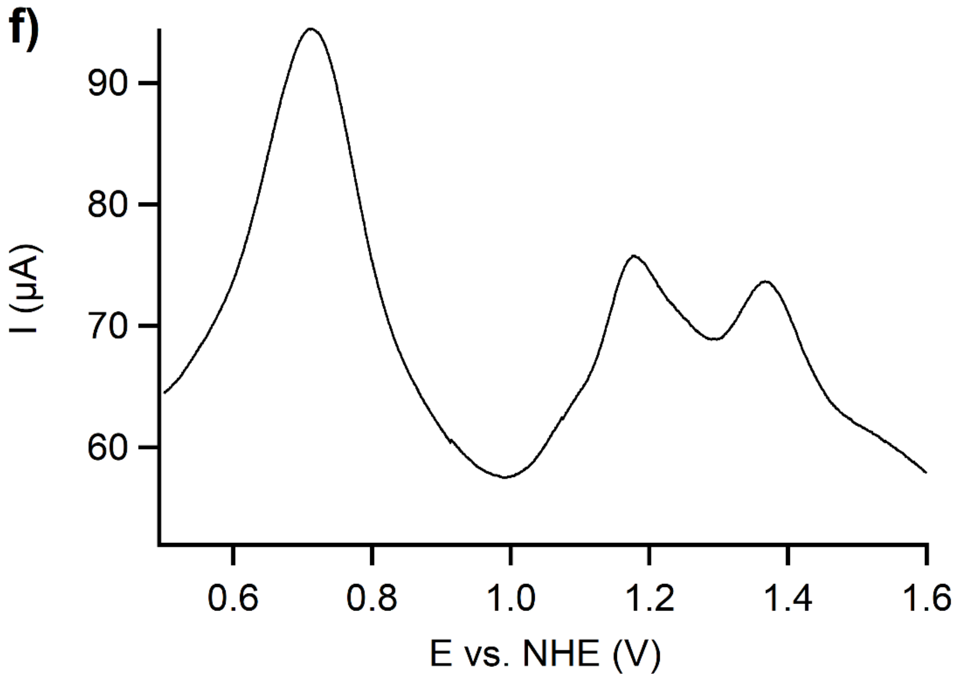
f)
90
80
70
60
I (μA)
0.6
0.8
1.0
1.2
1.4
1.6
E vs. NHE (V)

g)

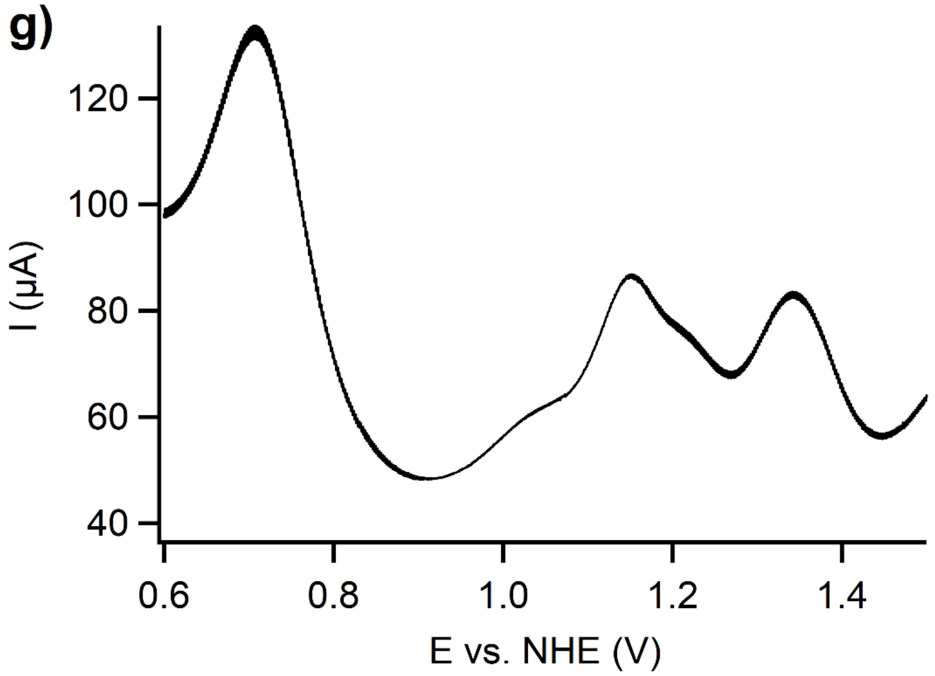

120
100
80
60
40
I (μA)
0.6
0.8
1.0
1.2
1.4
E vs. NHE (V)

h)

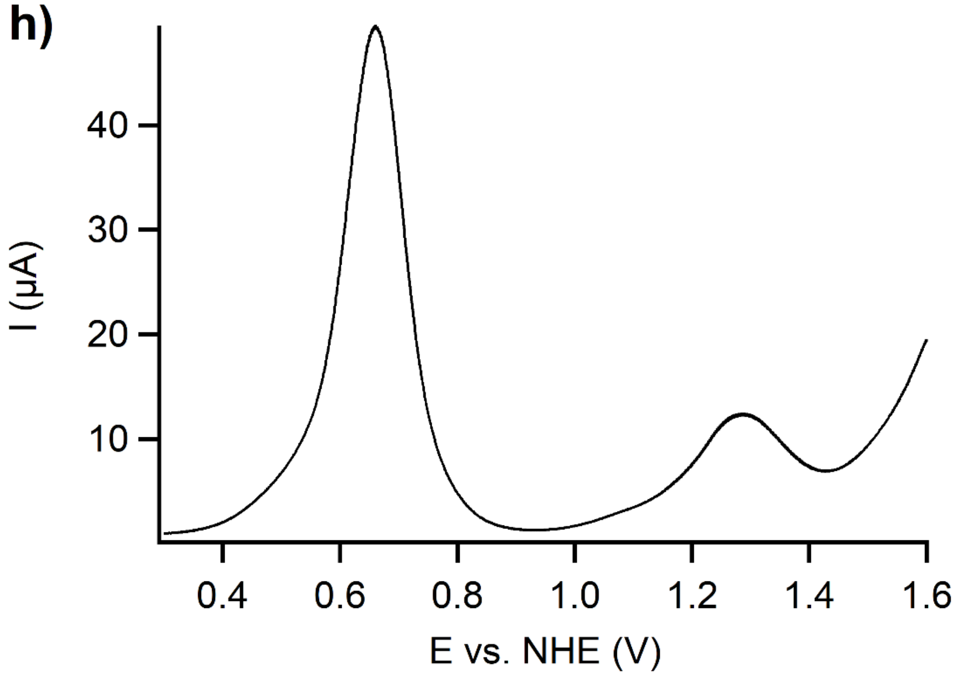

40
30
20
10
I (μA)
0.4
0.6
0.8
1.0
1.2
1.4
1.6
E vs. NHE (V)

i)

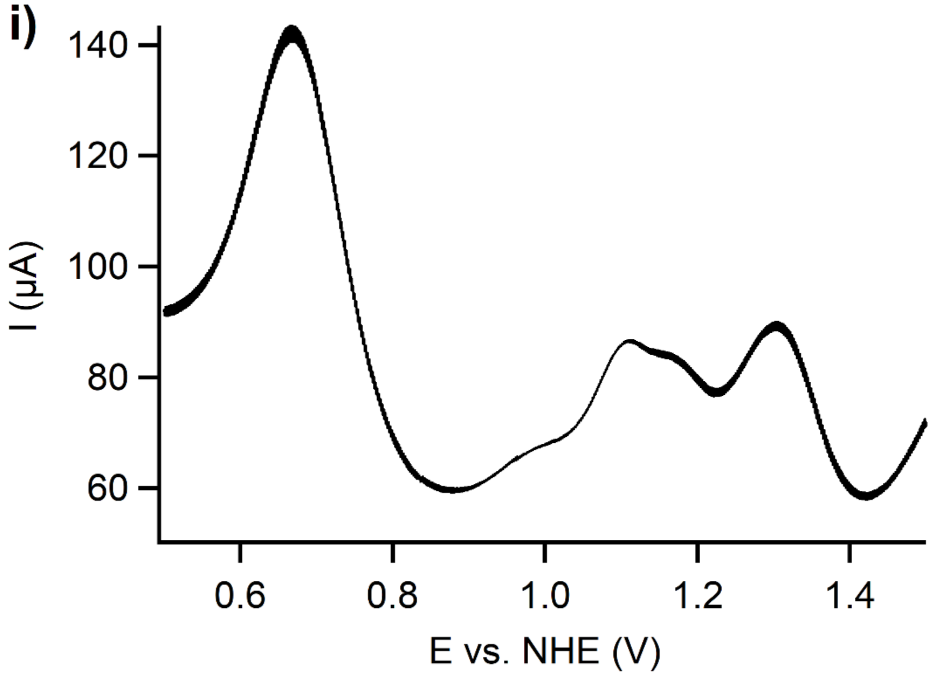
140
120
100
80
60
I (μA)
0.6
0.8
1.0
1.2
1.4
E vs. NHE (V)

j)

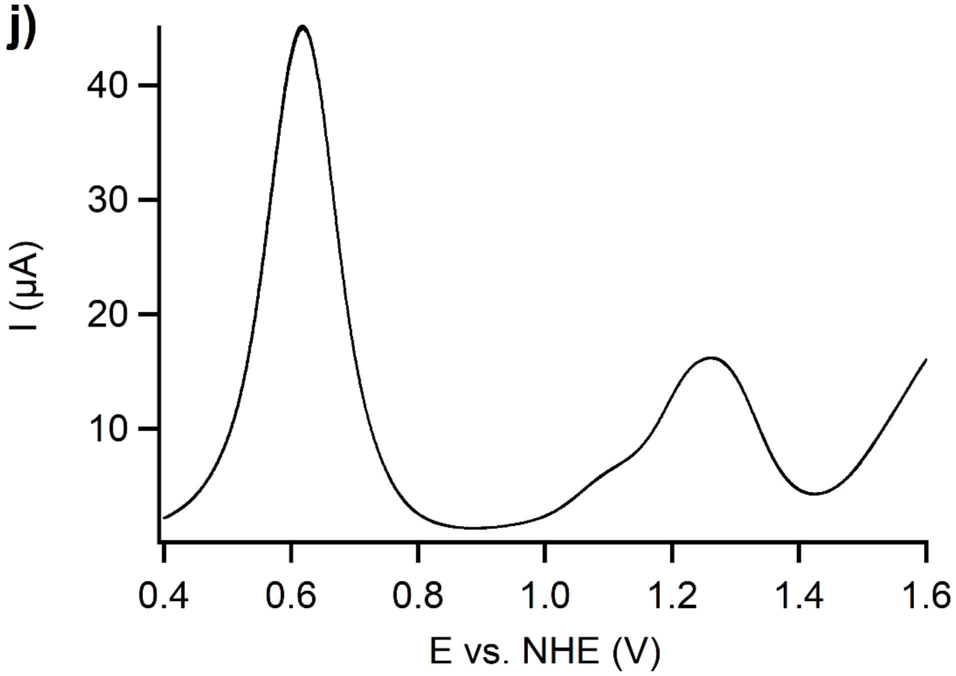
40
30
20
10
I (μA)
0.4
0.6
0.8
1.0
1.2
1.4
1.6
E vs. NHE (V)

k)

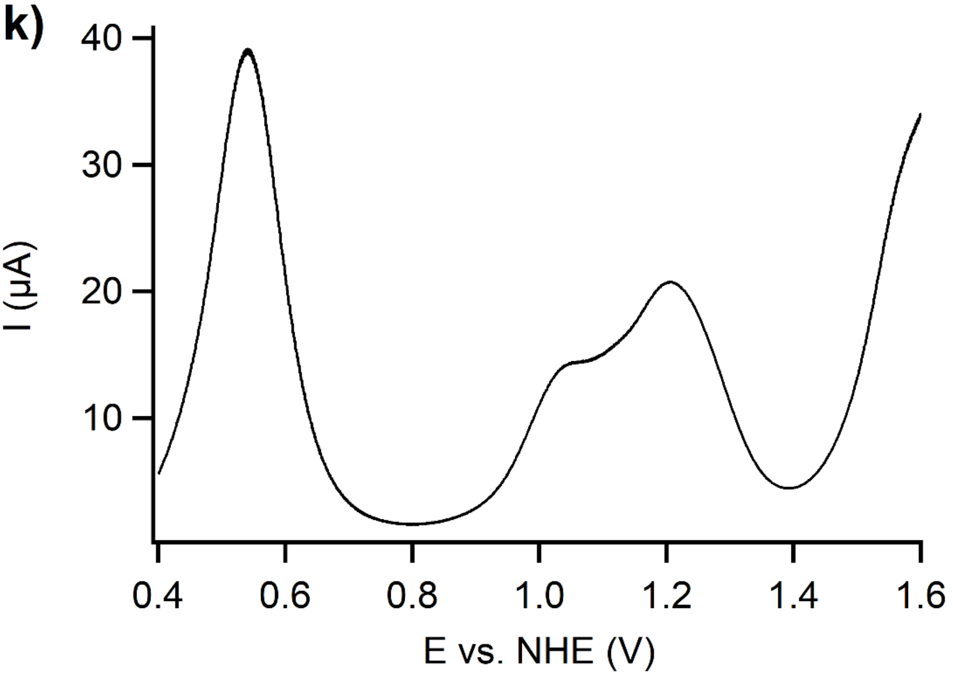
40
30
20
10
I (μA)
0.4
0.6
0.8
1.0
1.2
1.4
1.6
E vs. NHE (V)

l)

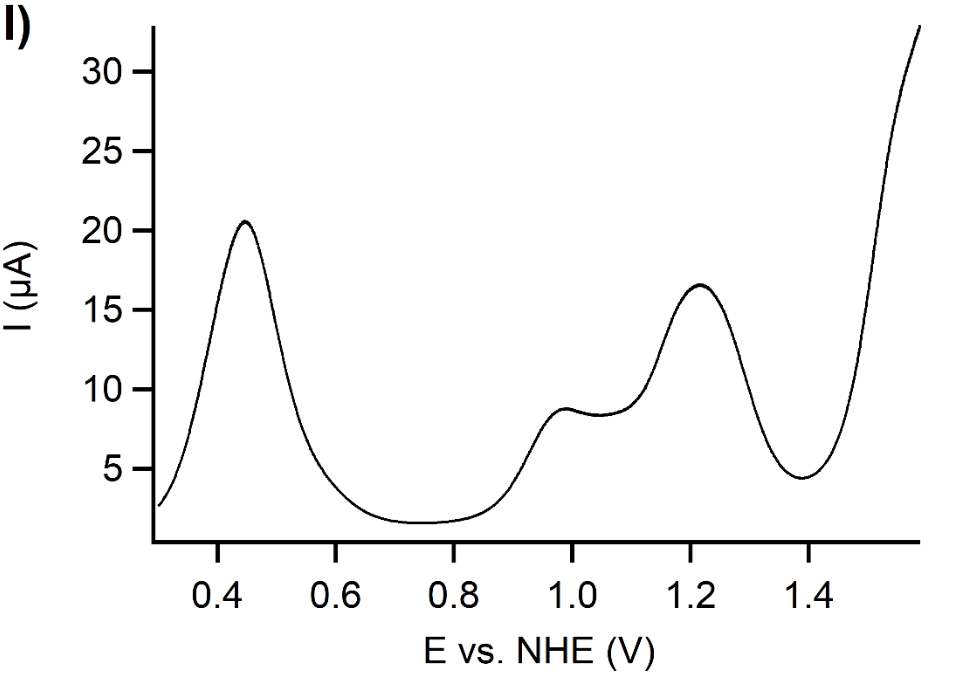
30
25
20
15
10
5
I (μA)
0.4
0.6
0.8
1.0
1.2
1.4
E vs. NHE (V)

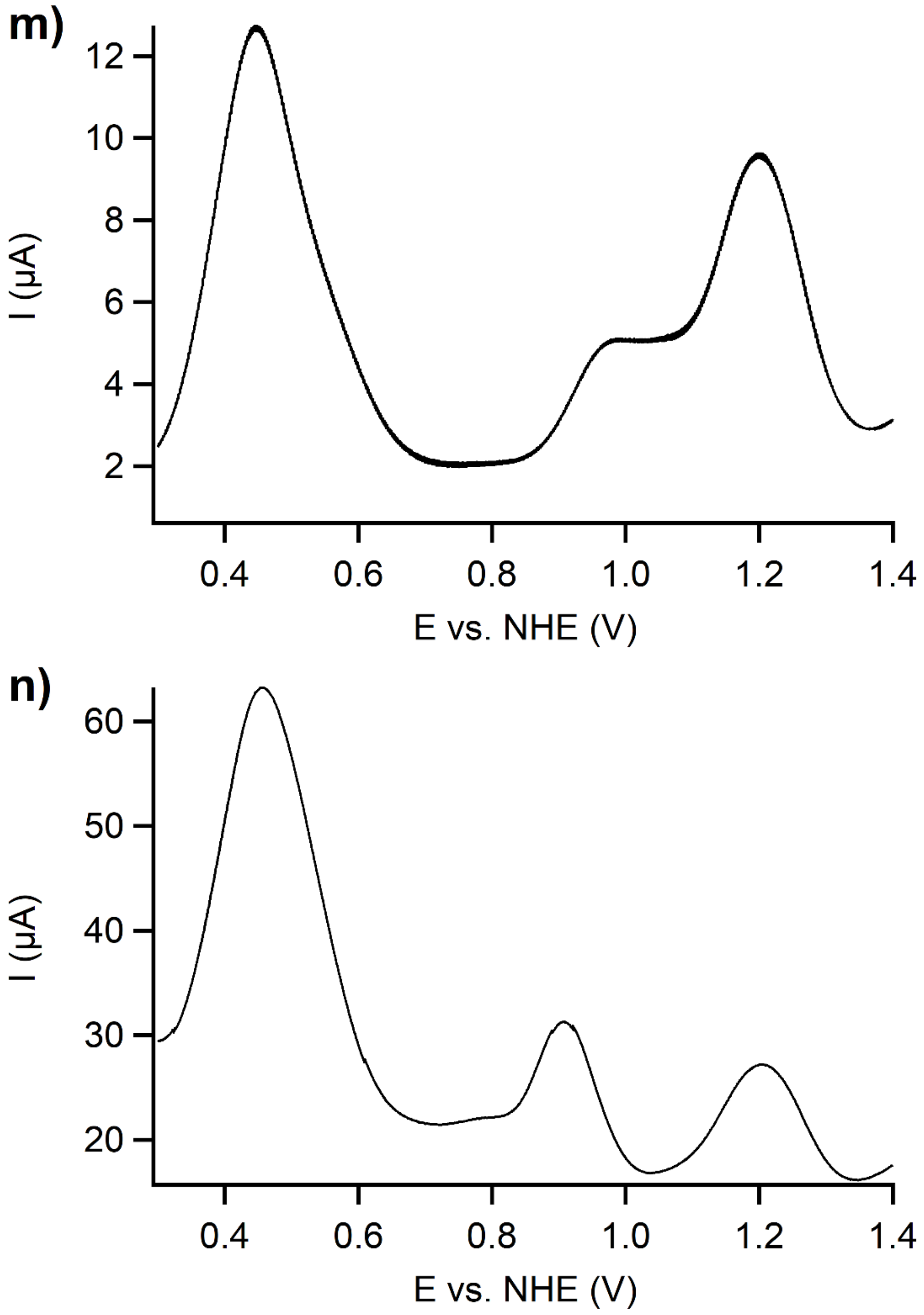
m)
I (μA)
12
10
8
6
4
2
0.4
0.6
0.8
1.0
1.2
1.4
E vs. NHE (V)
n)
I (μA)
60
50
40
30
20
0.4
0.6
0.8
1.0
1.2
1.4
E vs. NHE (V)

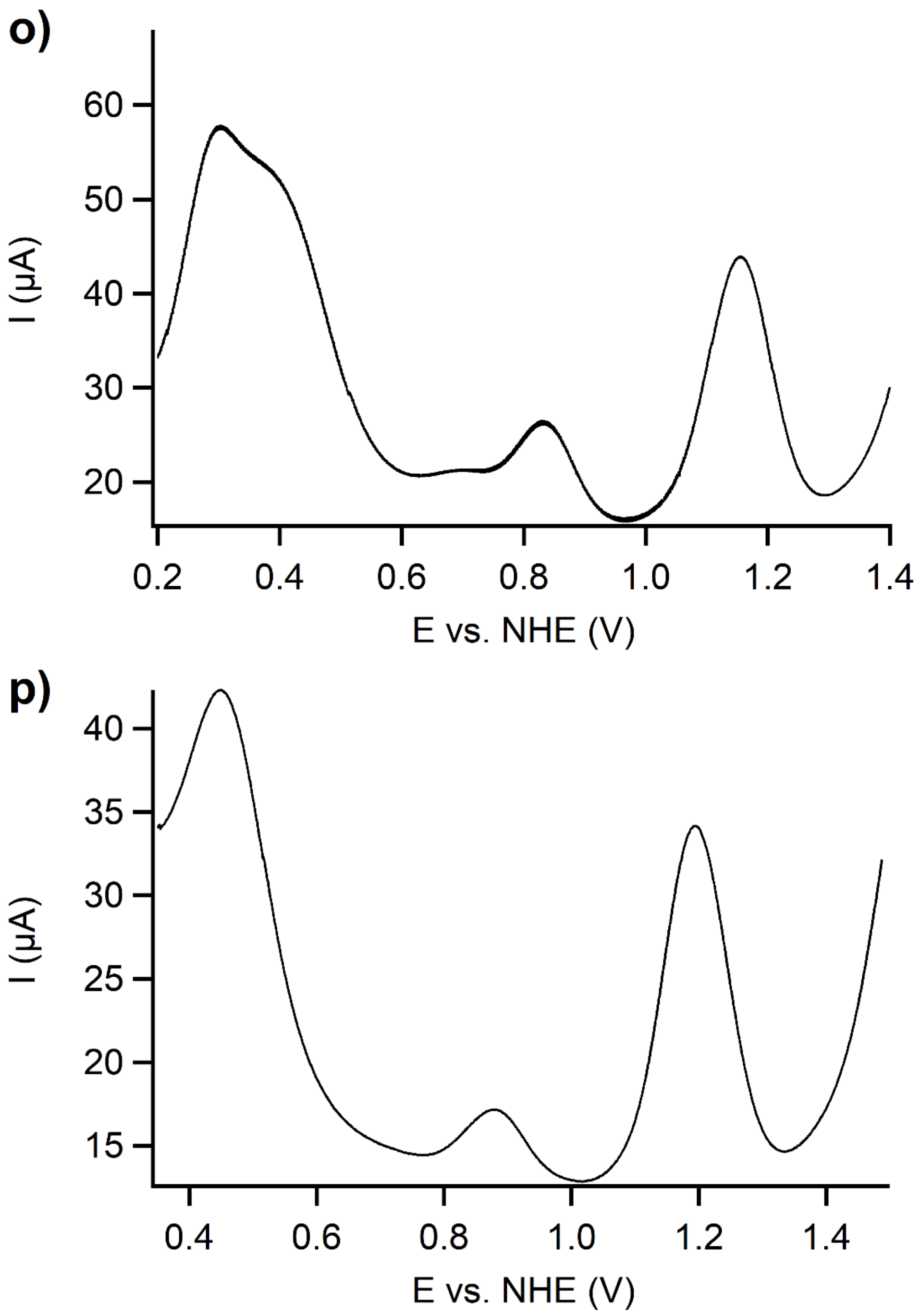

Figure S26. DPV data of *cis*-[Ru^{II}(4,4'-R_2-bpy)$_2$(H_2O)$_2$](OTf)$_2$ (**R=Me**) recorded in a 100 mM phosphate solution with the following pH values and electrodes: a) 1.055 GC b) 1.685 GC c) 2.153 GC d) 2.287 GC e) 2.435 BDD f) 2.435 GC g) 2.975 GC h) 3.941 BDD i) 3.972 GC j) 4.765 BDD k) 5.789 BDD l) 6.956 BDD m) 7.029 BDD n) 7.029 GC o) 7.954 GC p) 8.679 GC. Figures d) and o) have been measured with a 0.5 mM catalyst concentration, all others at a 1.0 mM concentration.

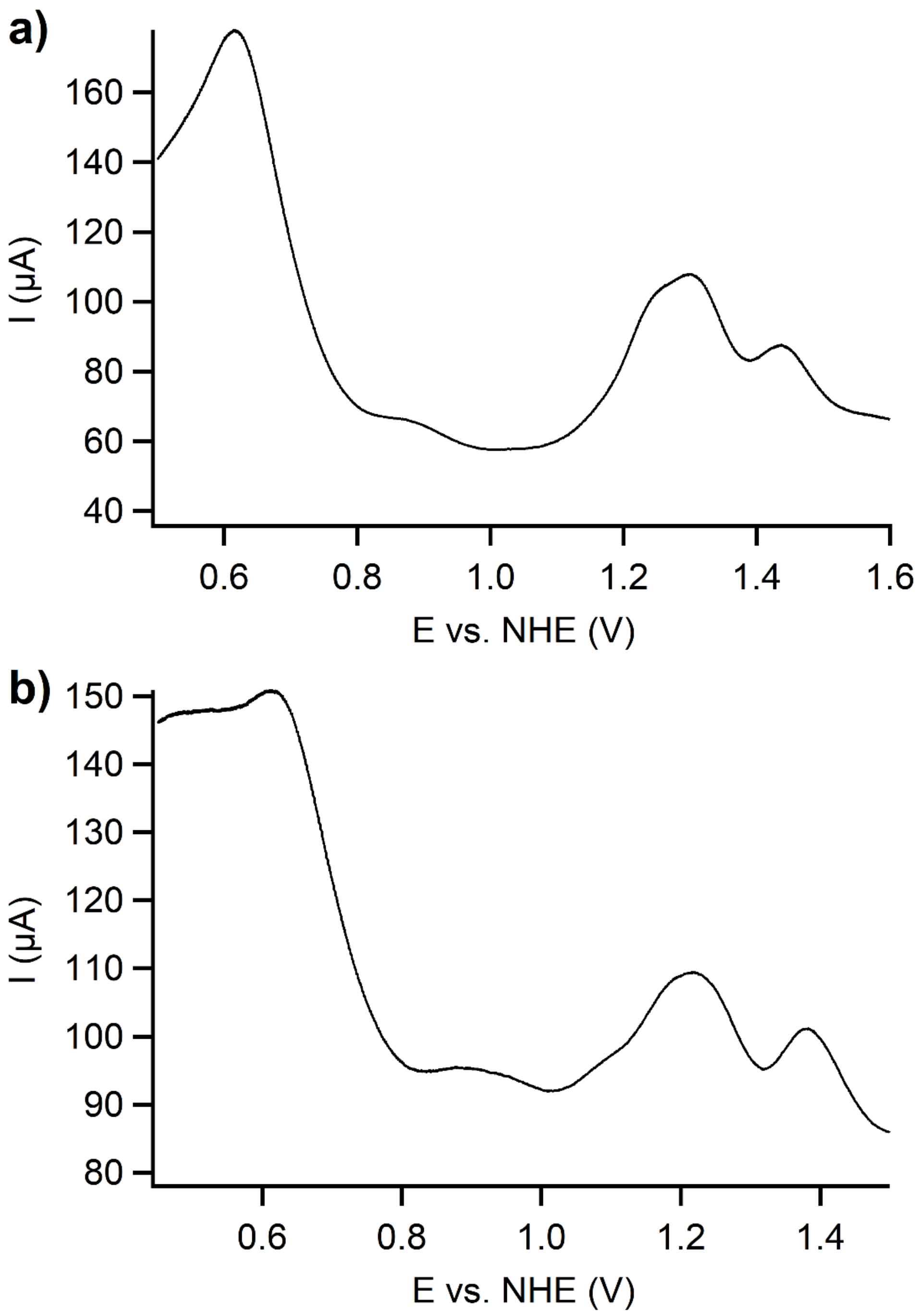
a)
160
140
120
100
80
60
40
I (µA)
0.6
0.8
1.0
1.2
1.4
1.6
E vs. NHE (V)
b)
150
140
130
120
110
100
90
80
I (µA)
0.6
0.8
1.0
1.2
1.4
E vs. NHE (V)

c)

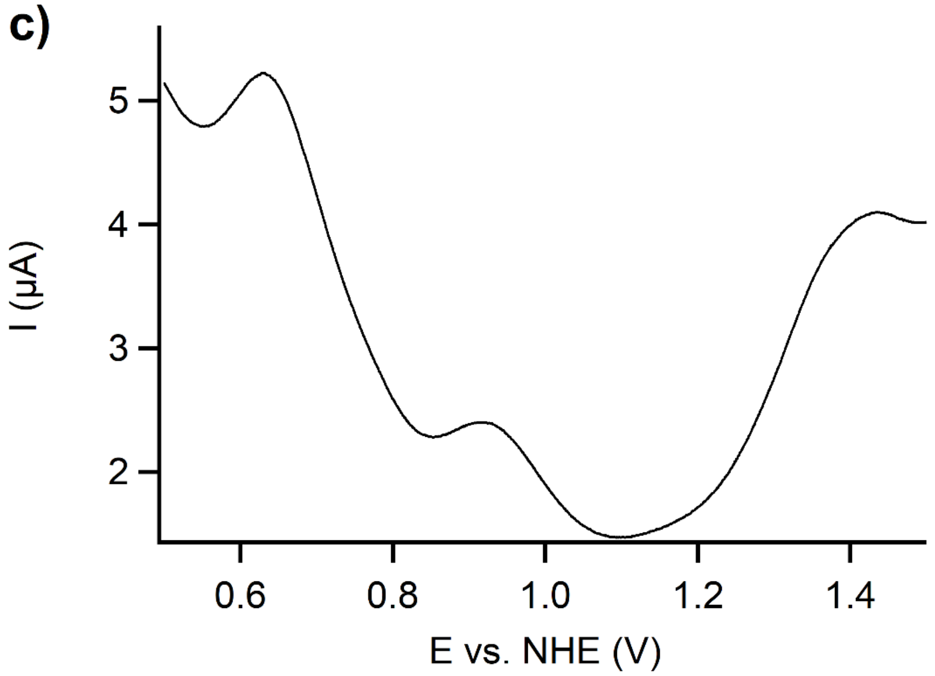

I (μA)
5
4
3
2
0.6
0.8
1.0
1.2
1.4
E vs. NHE (V)

d)

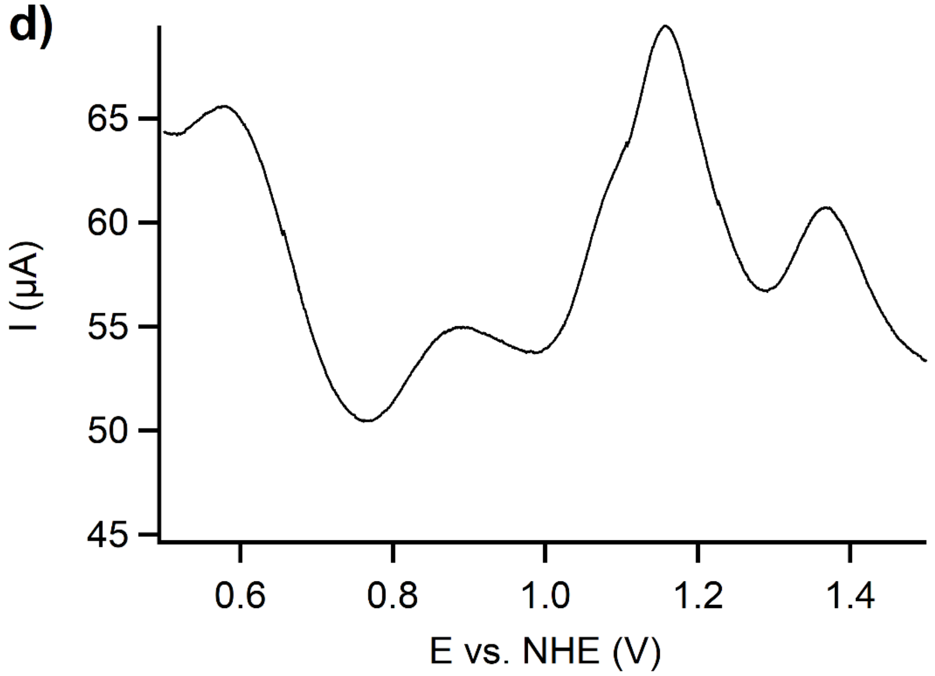

I (μA)
65
60
55
50
45
0.6
0.8
1.0
1.2
1.4
E vs. NHE (V)

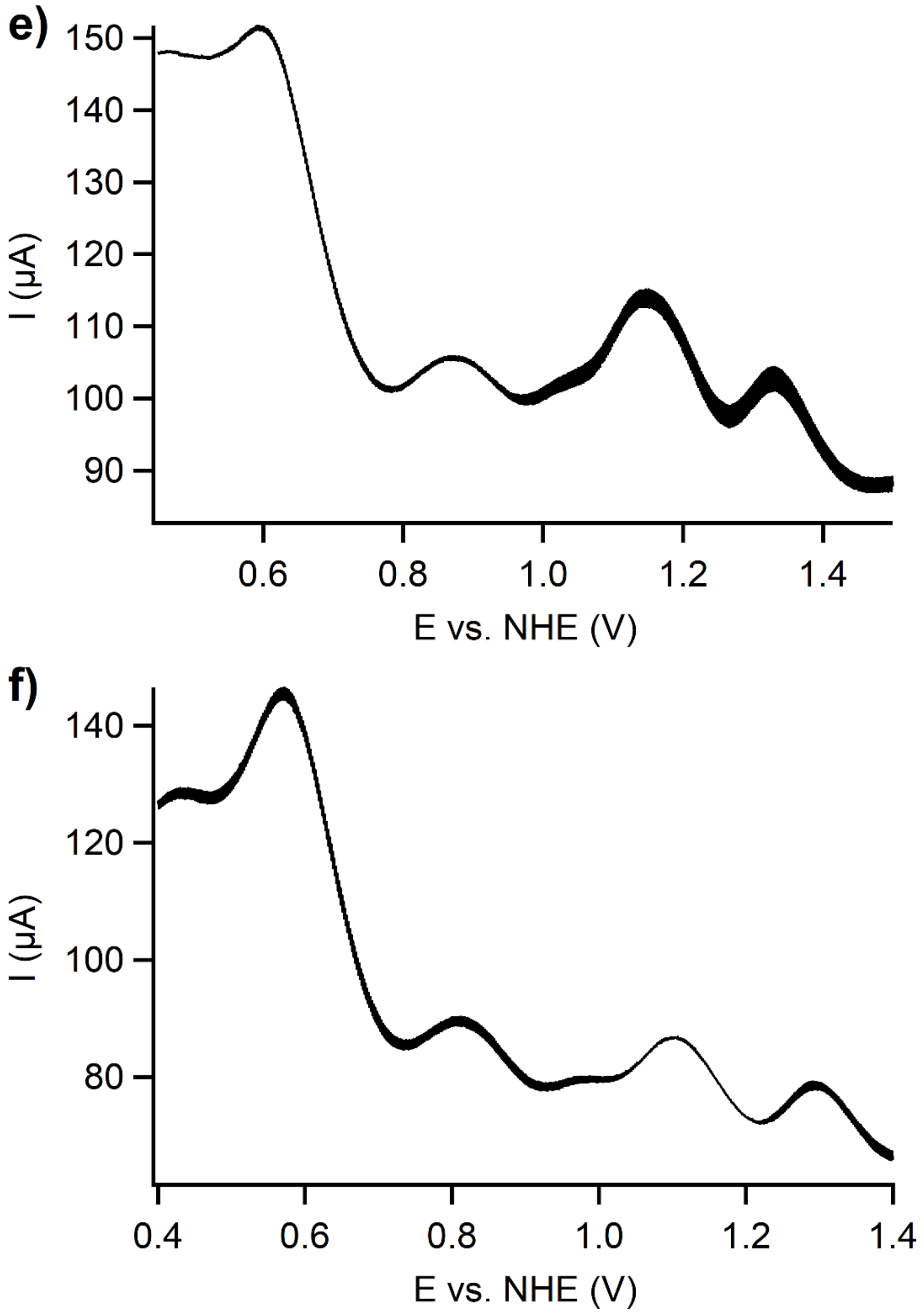
e)
150
140
130
120
110
100
90
I (µA)
0.6
0.8
1.0
1.2
1.4
E vs. NHE (V)
f)
140
120
100
80
I (µA)
0.4
0.6
0.8
1.0
1.2
1.4
E vs. NHE (V)

g)

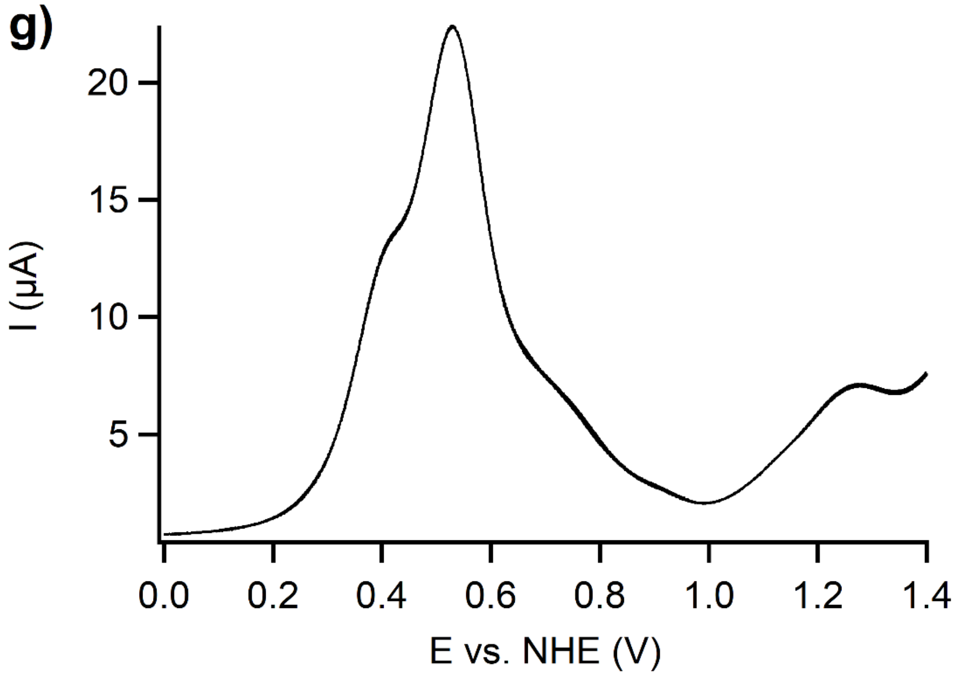
I (µA)
20
15
10
5
0.0
0.2
0.4
0.6
0.8
1.0
1.2
1.4
E vs. NHE (V)

h)

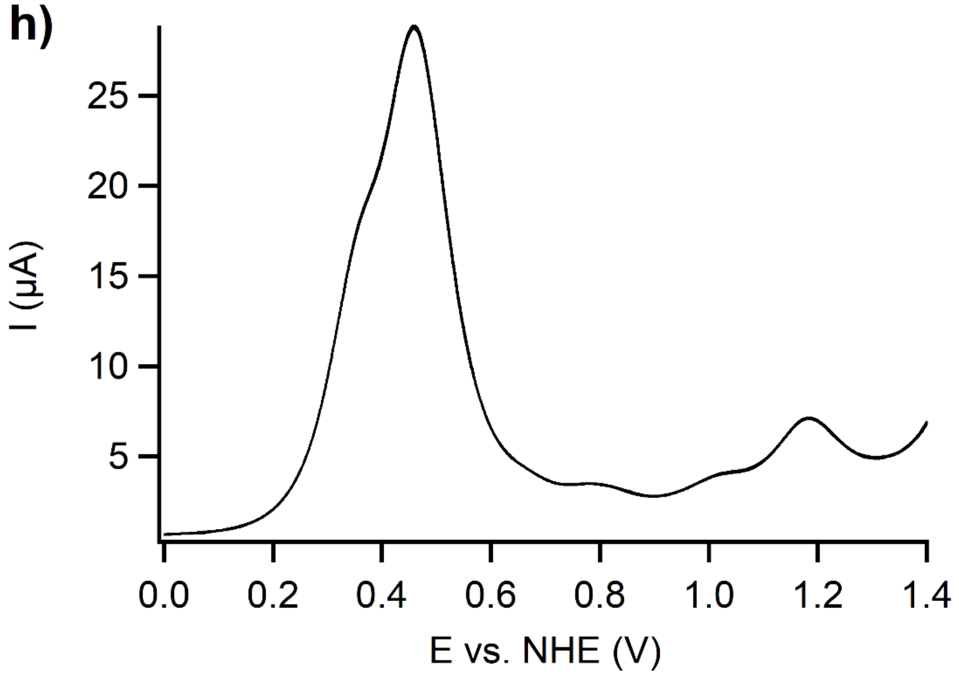
I (µA)
25
20
15
10
5
0.0
0.2
0.4
0.6
0.8
1.0
1.2
1.4
E vs. NHE (V)

i)

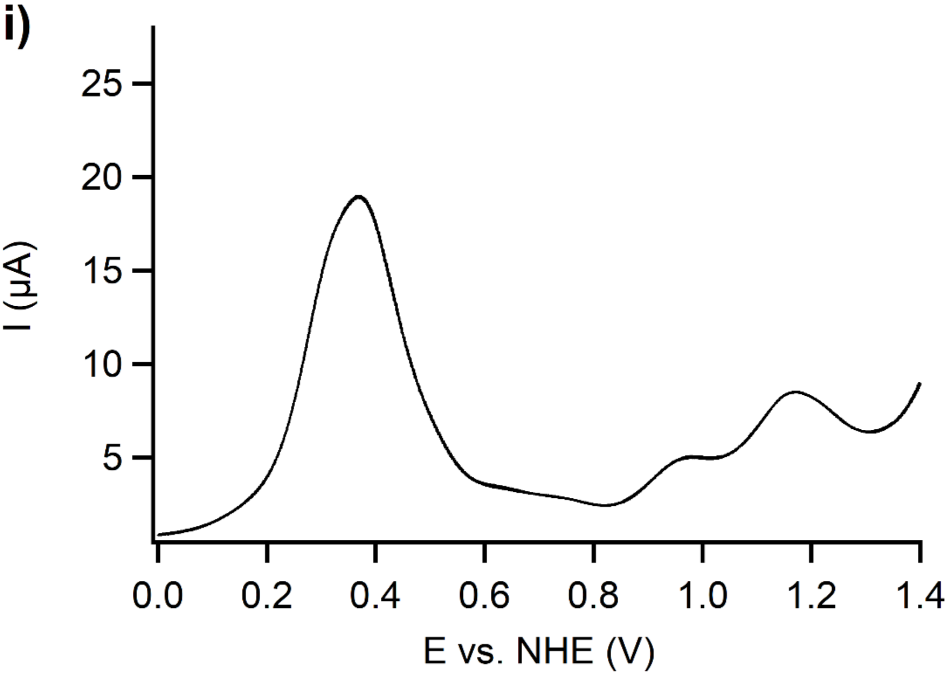

I (μA)
25
20
15
10
5
0.0
0.2
0.4
0.6
0.8
1.0
1.2
1.4
E vs. NHE (V)

j)

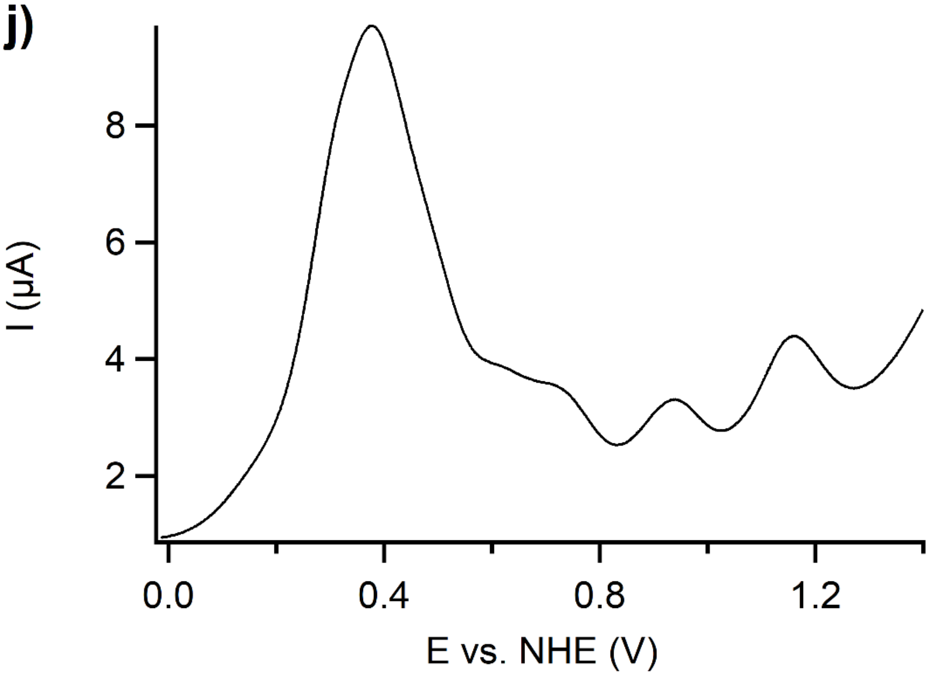

I (μA)
8
6
4
2
0.0
0.4
0.8
1.2
E vs. NHE (V)

k)

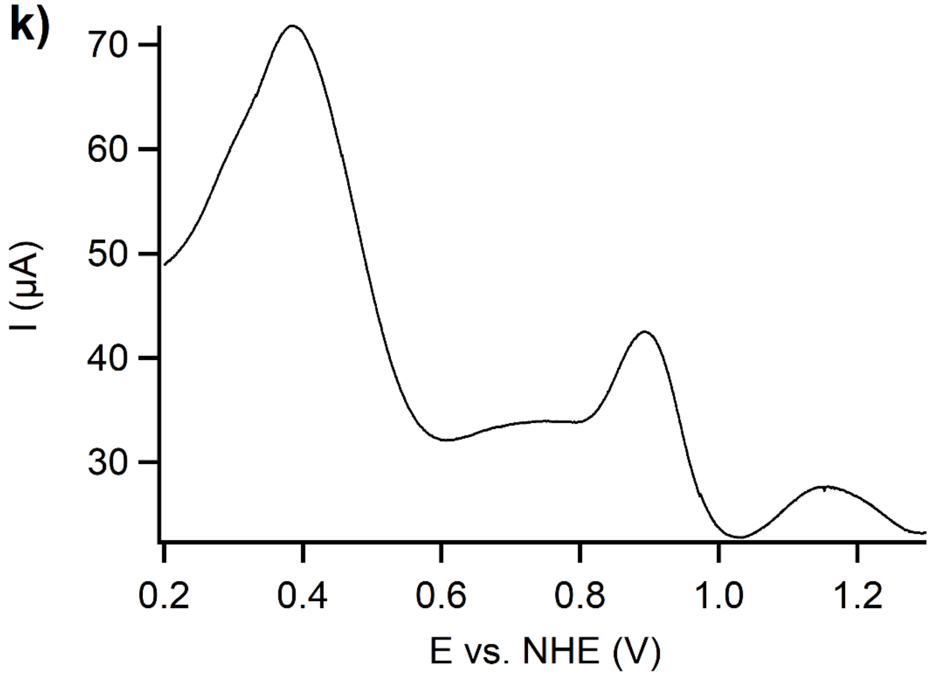
70
60
50
40
30
I (µA)
0.2
0.4
0.6
0.8
1.0
1.2
E vs. NHE (V)

l)

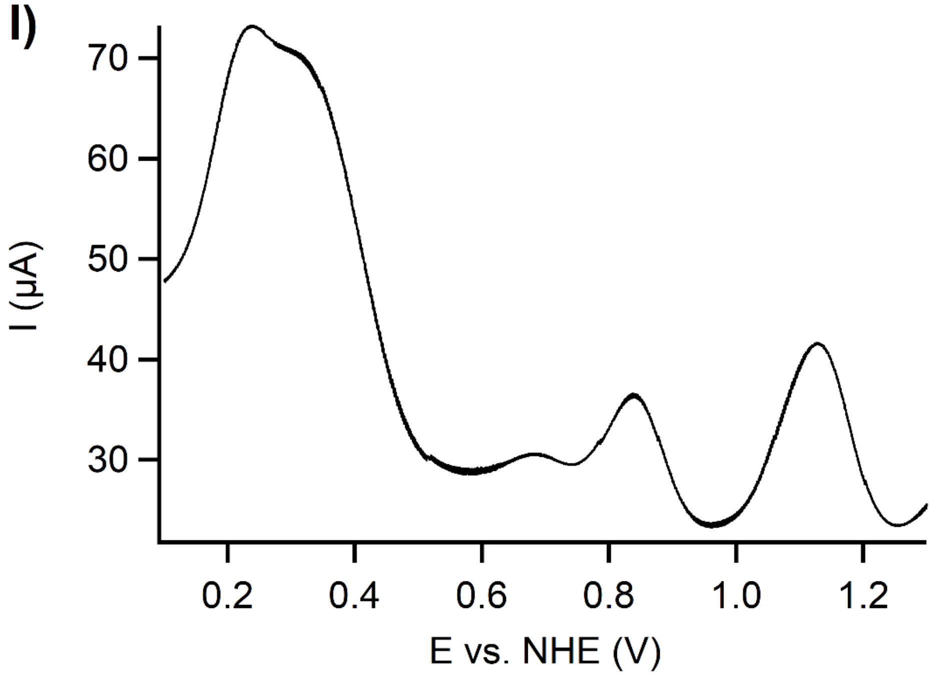
70
60
50
40
30
I (µA)
0.2
0.4
0.6
0.8
1.0
1.2
E vs. NHE (V)

m)

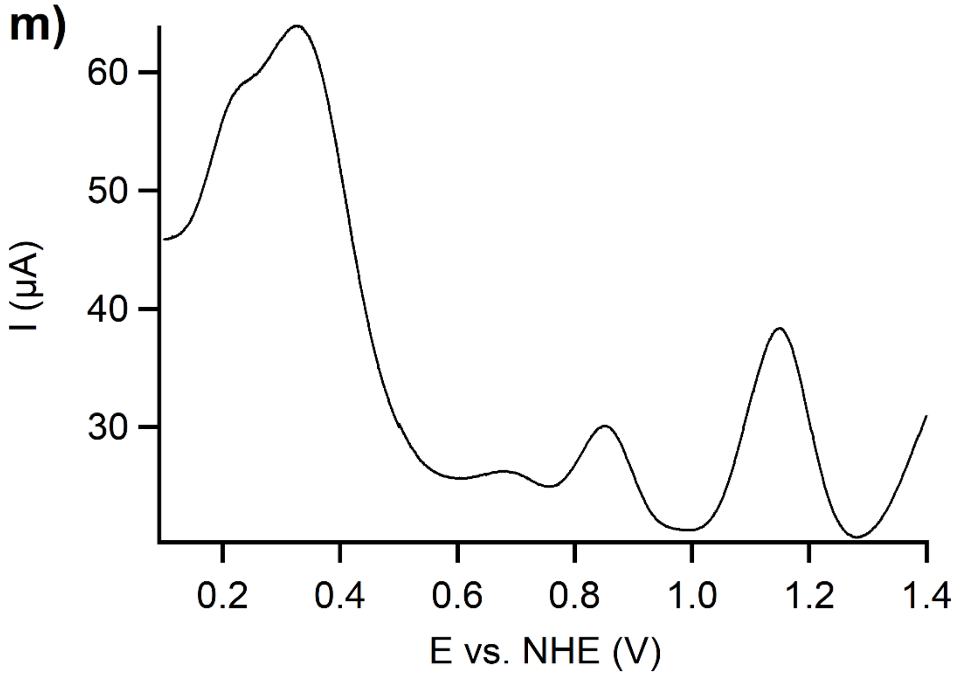

Figure S27. DPV data of *cis*-[Ru^{II}(4,4'-R_2-bpy)$_2$(H_2O)$_2$](OTf)$_2$ (R=OMe) recorded in a 100 mM phosphate solution with the following pH values and electrodes: a) 1.144 GC b) 2.123 GC c) 2.475 BDD d) 2.475 GC e) 3.020 GC f) 3.917 GC g) 4.749 BDD h) 5.845 BDD i) 6.93 BDD j) 7.087 BDD k) 7.087 GC l) 7.913 GC m) 8.485 GC. Figures c), d), j) and k) were measured at a 0.5 mM catalyst concentration, all others at a 1.0 mM concentration.

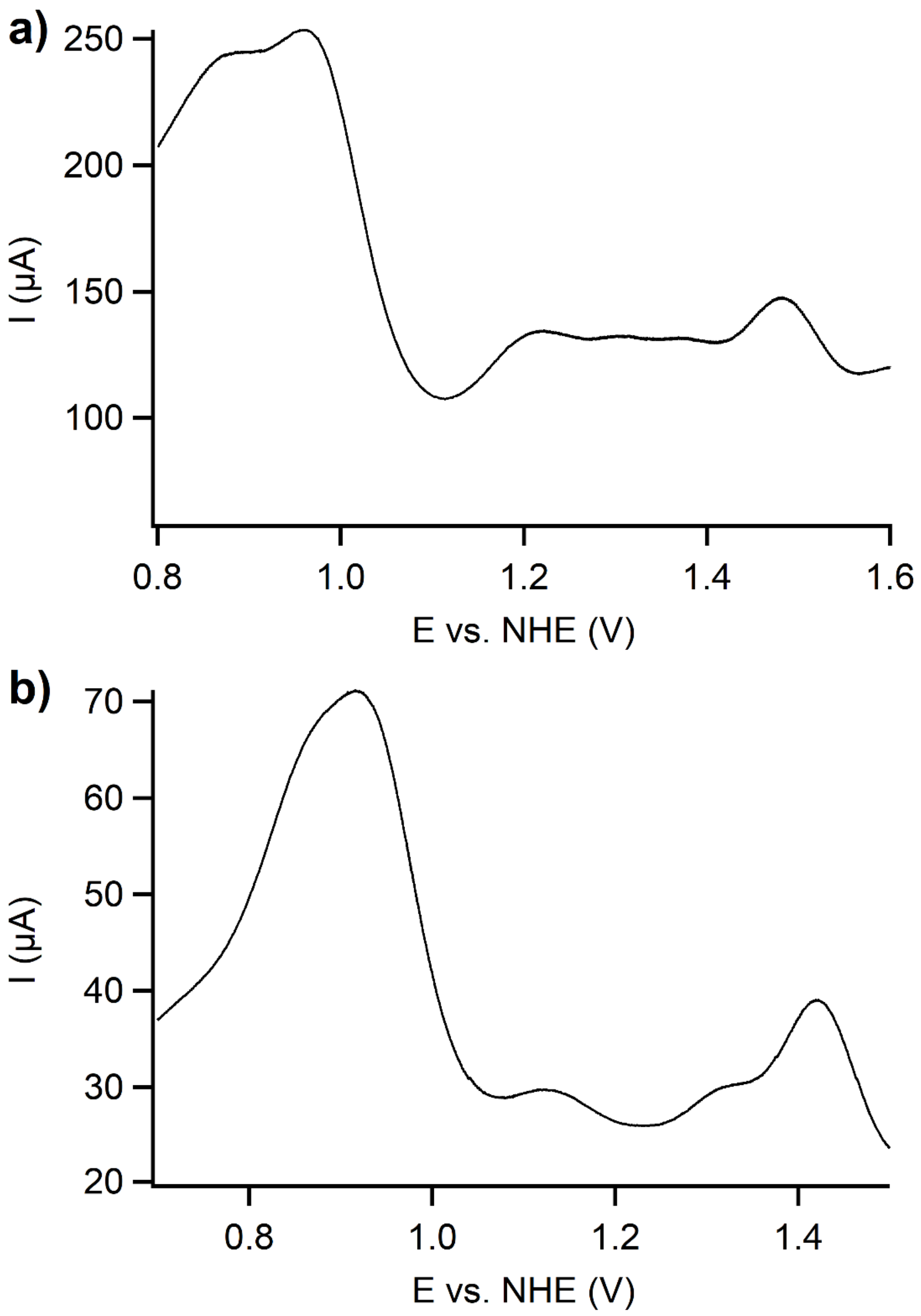
a)
250
200
150
100
I (µA)
0.8
1.0
1.2
1.4
1.6
E vs. NHE (V)
b)
70
60
50
40
30
20
I (µA)
0.8
1.0
1.2
1.4
E vs. NHE (V)

c)

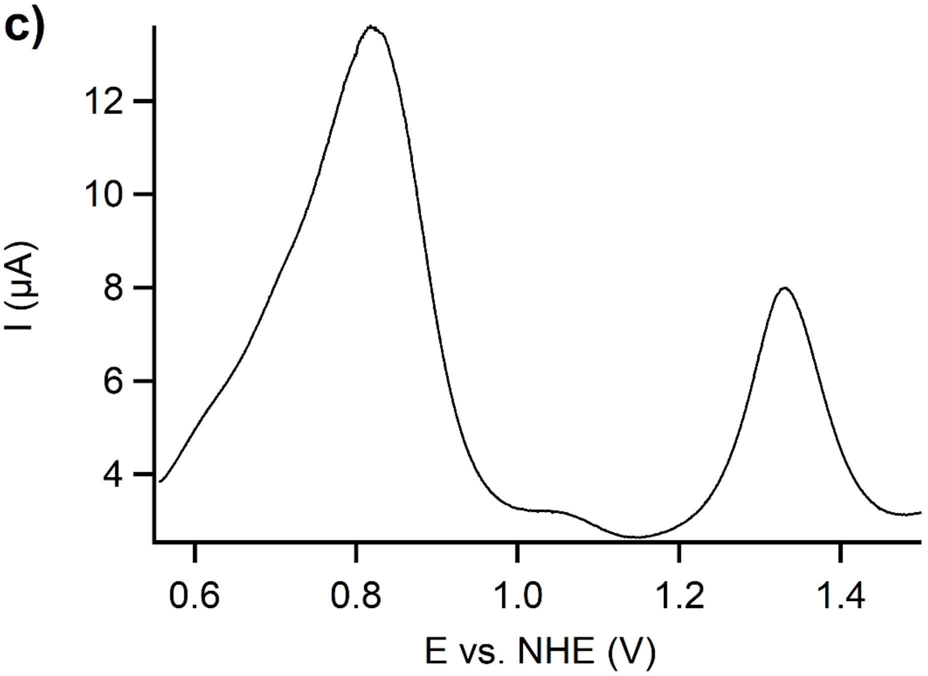
12
10
8
6
4
I (µA)
0.6
0.8
1.0
1.2
1.4
E vs. NHE (V)

d)

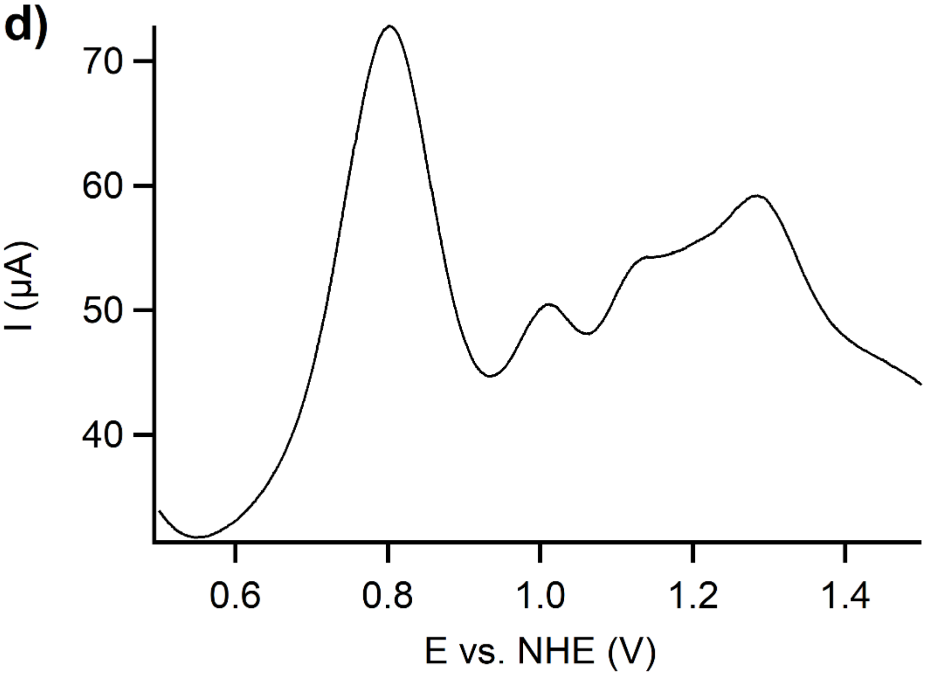
70
60
50
40
I (µA)
0.6
0.8
1.0
1.2
1.4
E vs. NHE (V)

e)

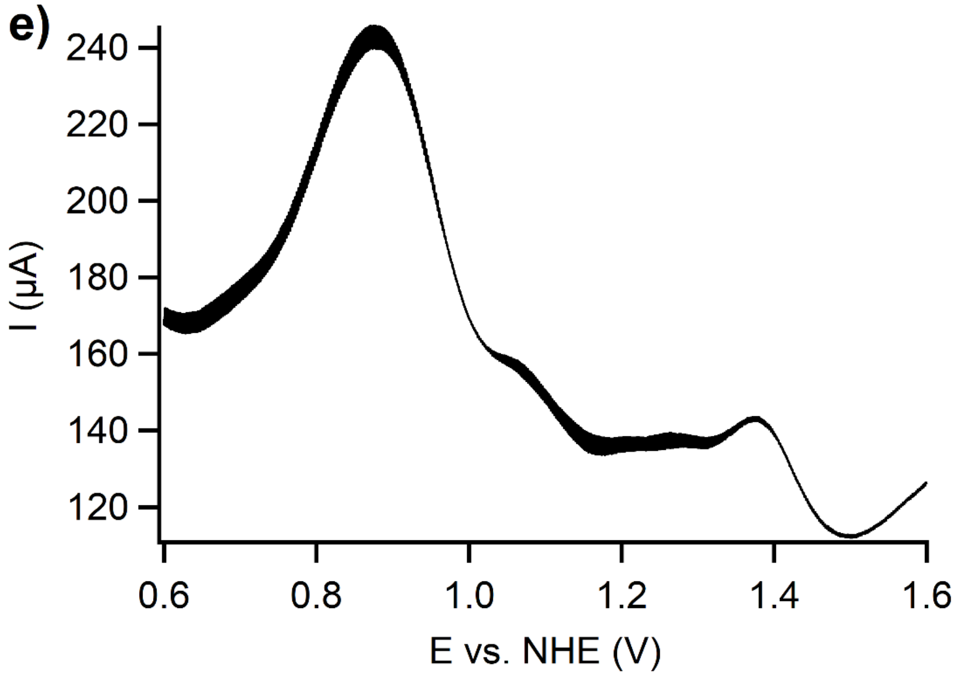
240
220
200
180
160
140
120
I (μA)
0.6
0.8
1.0
1.2
1.4
1.6
E vs. NHE (V)

f)

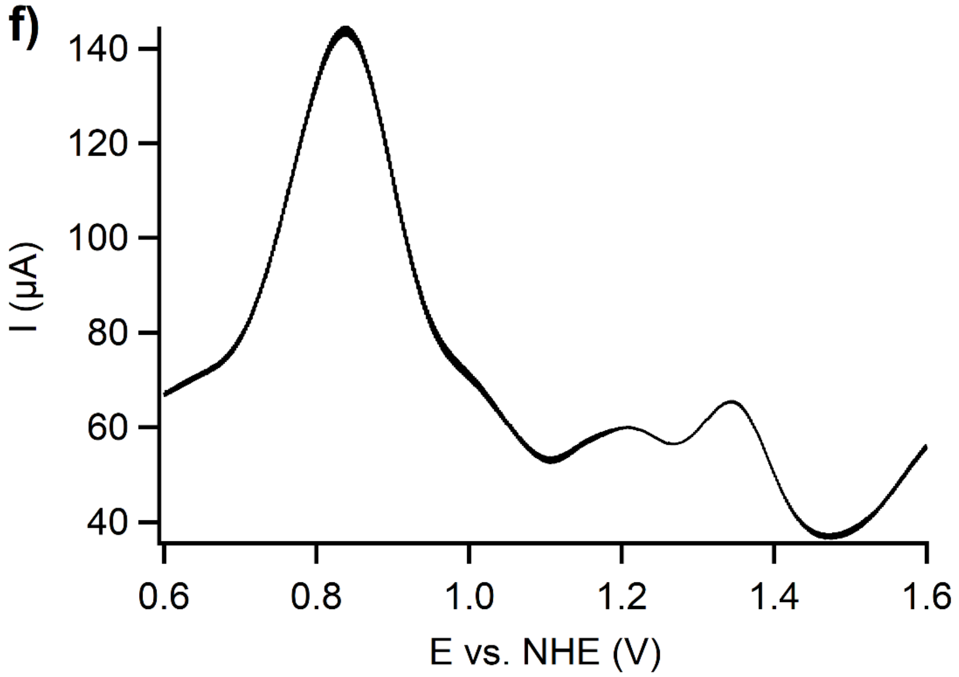
140
120
100
80
60
40
I (μA)
0.6
0.8
1.0
1.2
1.4
1.6
E vs. NHE (V)

g)

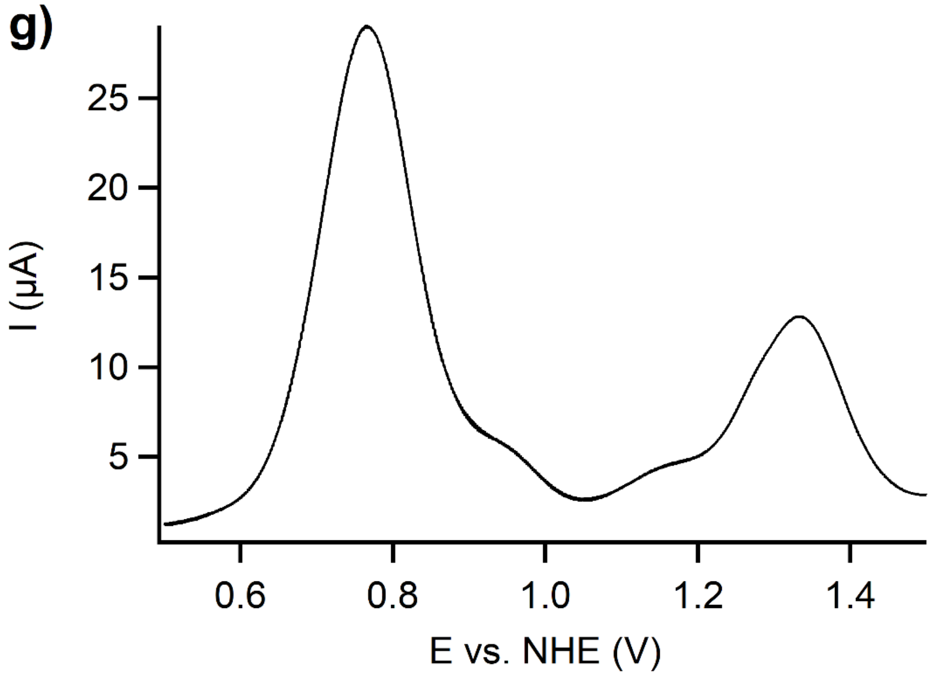

I (μA)
25
20
15
10
5
0.6
0.8
1.0
1.2
1.4
E vs. NHE (V)

h)

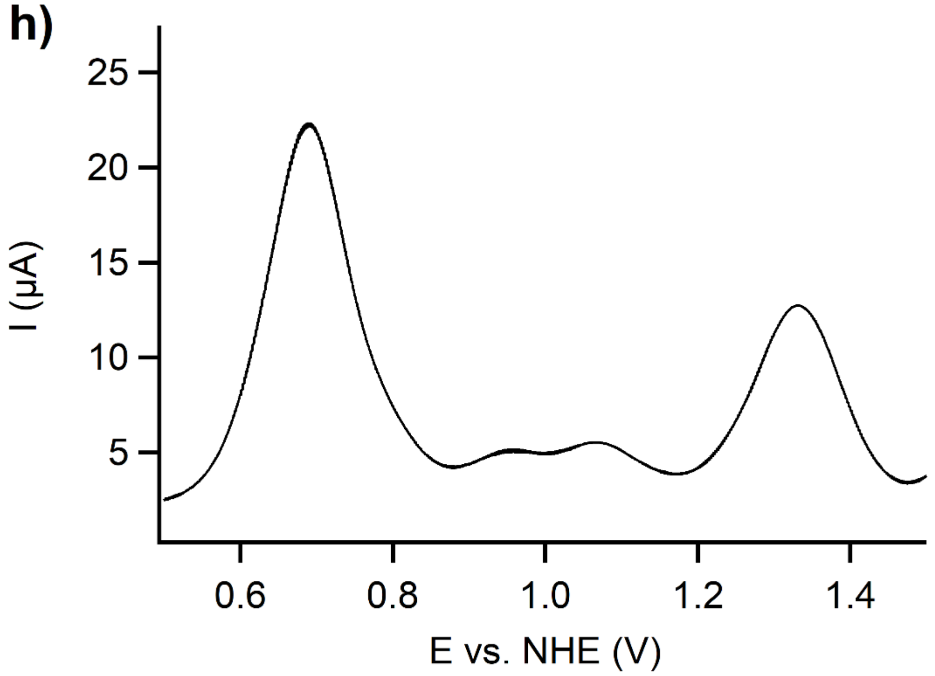

I (μA)
25
20
15
10
5
0.6
0.8
1.0
1.2
1.4
E vs. NHE (V)

i)

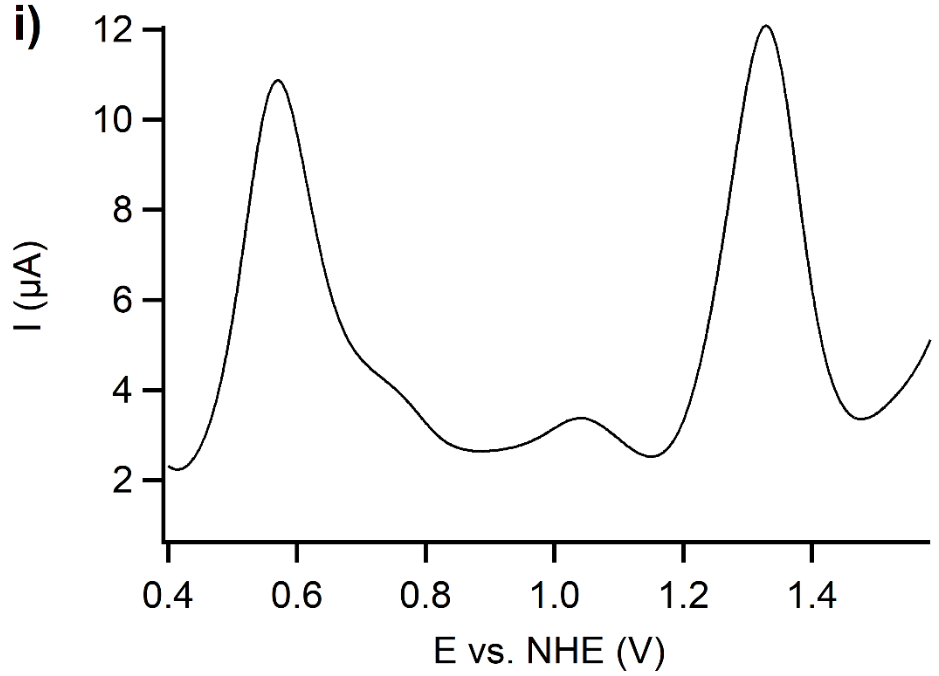
I (μA)
12
10
8
6
4
2
0.4
0.6
0.8
1.0
1.2
1.4
E vs. NHE (V)

j)

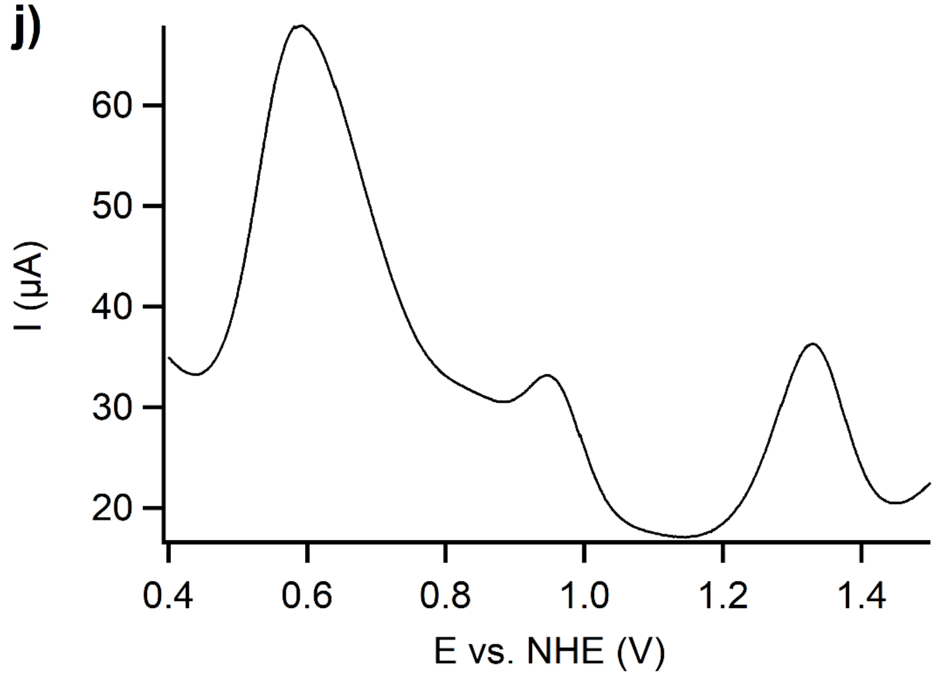
I (μA)
60
50
40
30
20
0.4
0.6
0.8
1.0
1.2
1.4
E vs. NHE (V)

k)

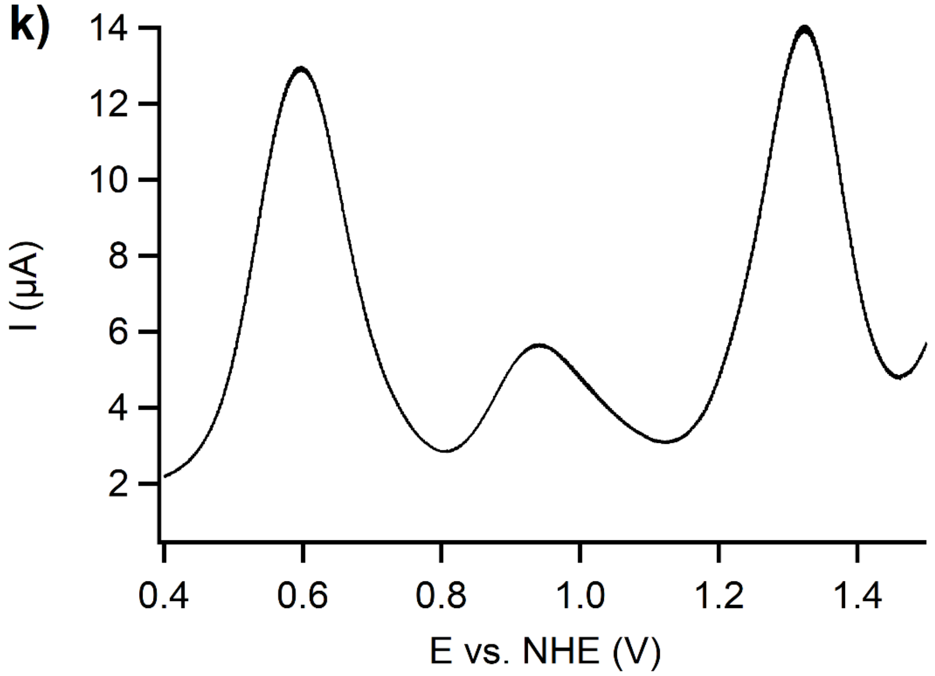

I (μA)
14
12
10
8
6
4
2
0.4
0.6
0.8
1.0
1.2
1.4
E vs. NHE (V)

l)

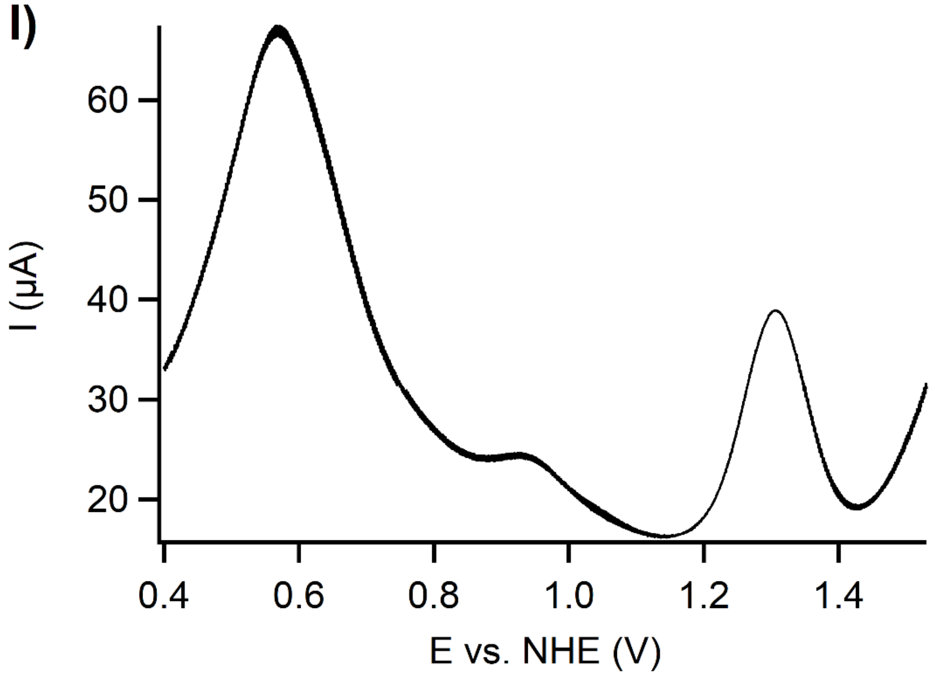

I (μA)
60
50
40
30
20
0.4
0.6
0.8
1.0
1.2
1.4
E vs. NHE (V)

m)

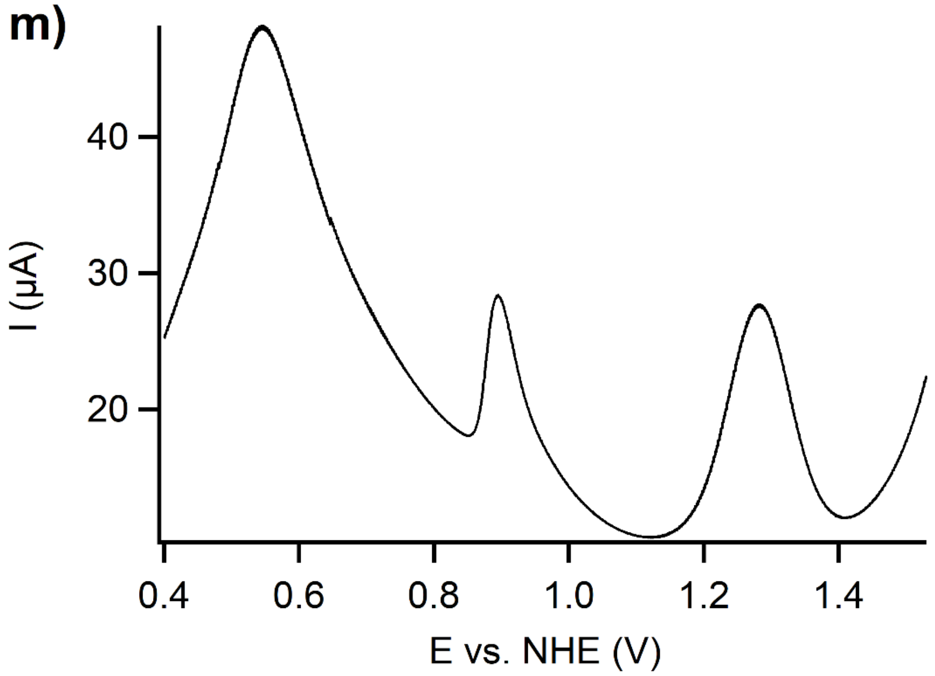
40
30
20
I (μA)
0.4
0.6
0.8
1.0
1.2
1.4
E vs. NHE (V)

n)

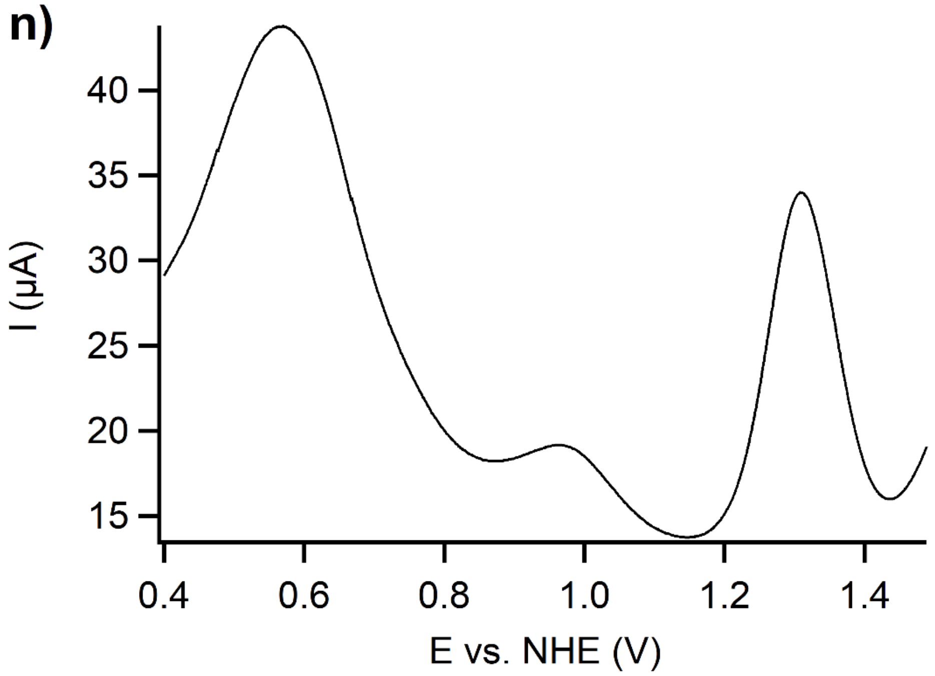
40
35
30
25
20
15
I (μA)
0.4
0.6
0.8
1.0
1.2
1.4
E vs. NHE (V)

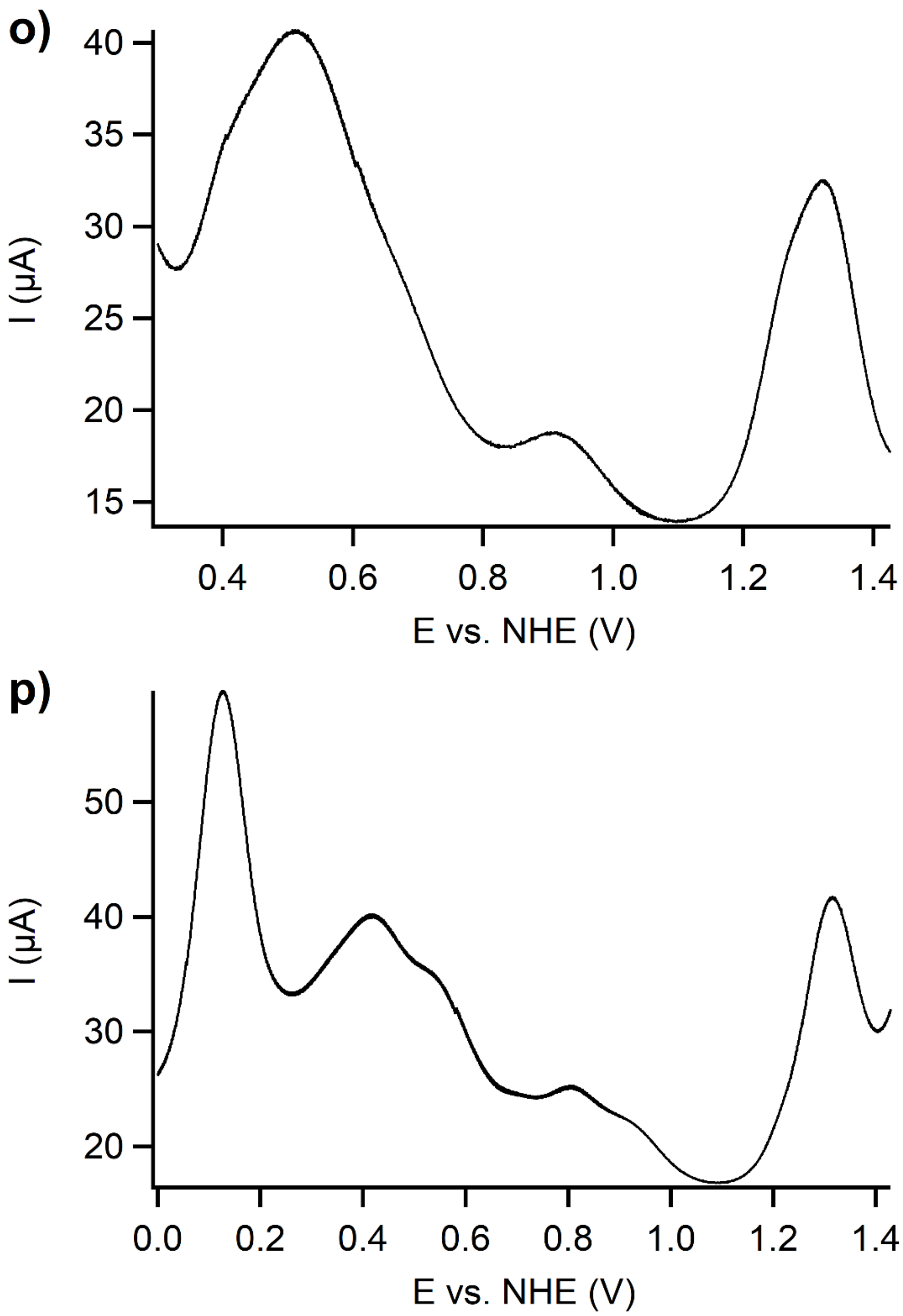

Figure S28. DPV data of *cis*-[Ru^{II}(4,4'-R_2-bpy)$_2$(H_2O)$_2$](OTf)$_2$ (**R=Cl**) recorded in a 100 mM phosphate solution with the following pH values and electrodes: a) 1.025 GC b) 2.154 GC c) 2.437 BDD d) 2.437 GC e) 3.017 f) 3.886 GC g) 4.802 BDD h) 5.850 BDD i) 7.014 j) 7.014 k) 7.091 BDD l) 7.936 m) 7.938 GC n) 8.652 GC o) 9.700 GC p) 11.353 GC. Figures c) and i) have been measured at a 0.5 mM catalyst concentration, all others at a 1.0 mM concentration.

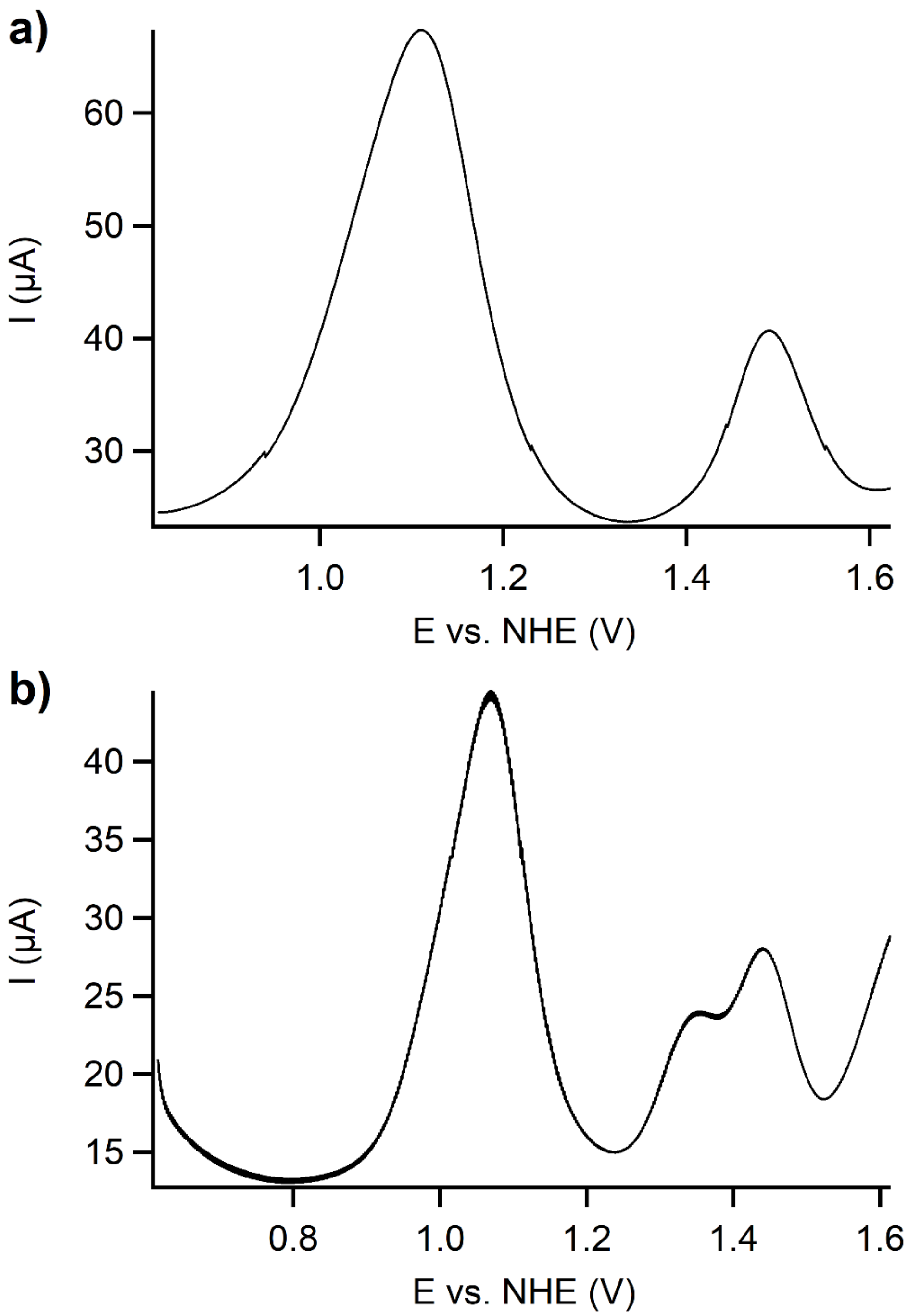
a)
60
50
40
30
I (μA)
1.0
1.2
1.4
1.6
E vs. NHE (V)
b)
40
35
30
25
20
15
I (μA)
0.8
1.0
1.2
1.4
1.6
E vs. NHE (V)

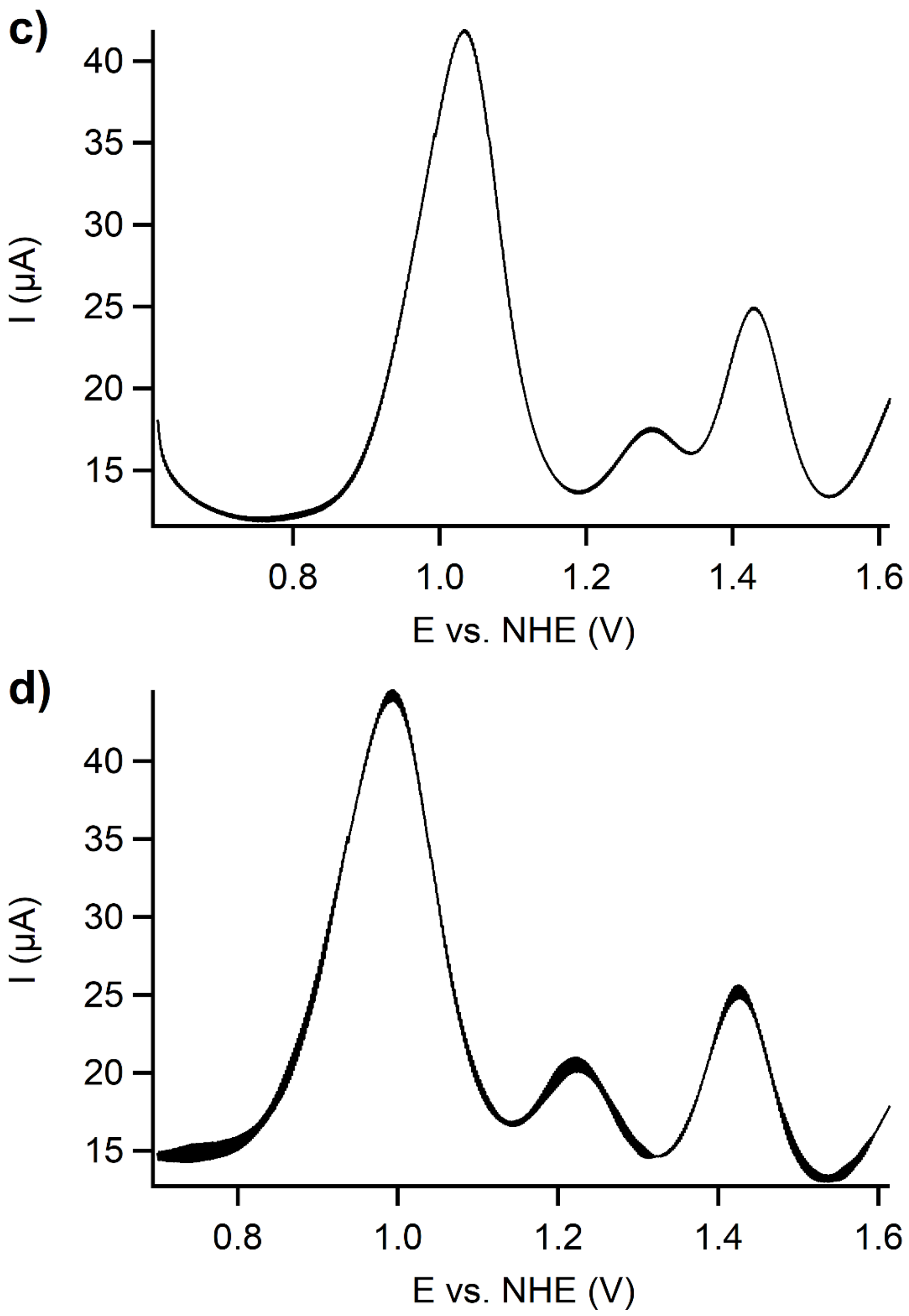

c)
I (μA)
40
35
30
25
20
15
0.8
1.0
1.2
1.4
1.6
E vs. NHE (V)
d)
I (μA)
40
35
30
25
20
15
0.8
1.0
1.2
1.4
1.6
E vs. NHE (V)

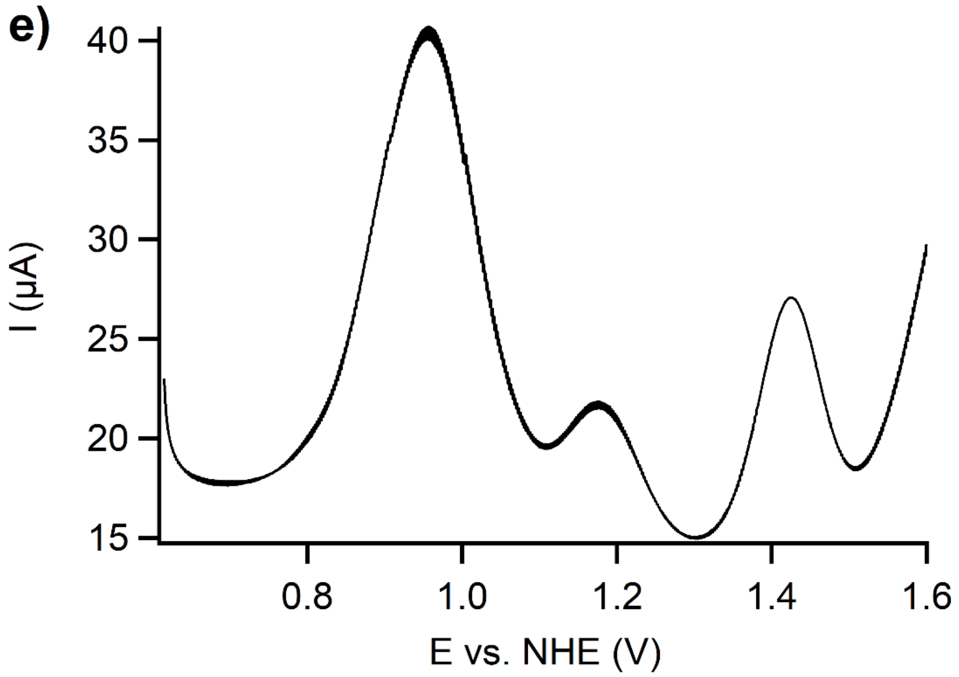

e)
40
35
30
25
20
15
I (μA)
0.8
1.0
1.2
1.4
1.6
E vs. NHE (V)

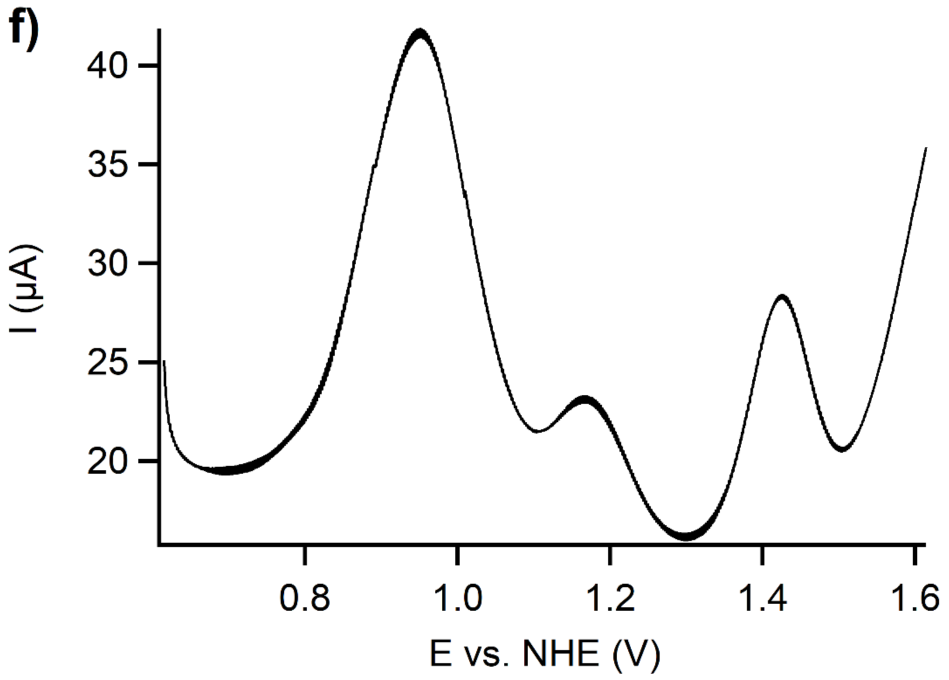

f)
40
35
30
25
20
I (μA)
0.8
1.0
1.2
1.4
1.6
E vs. NHE (V)

h)

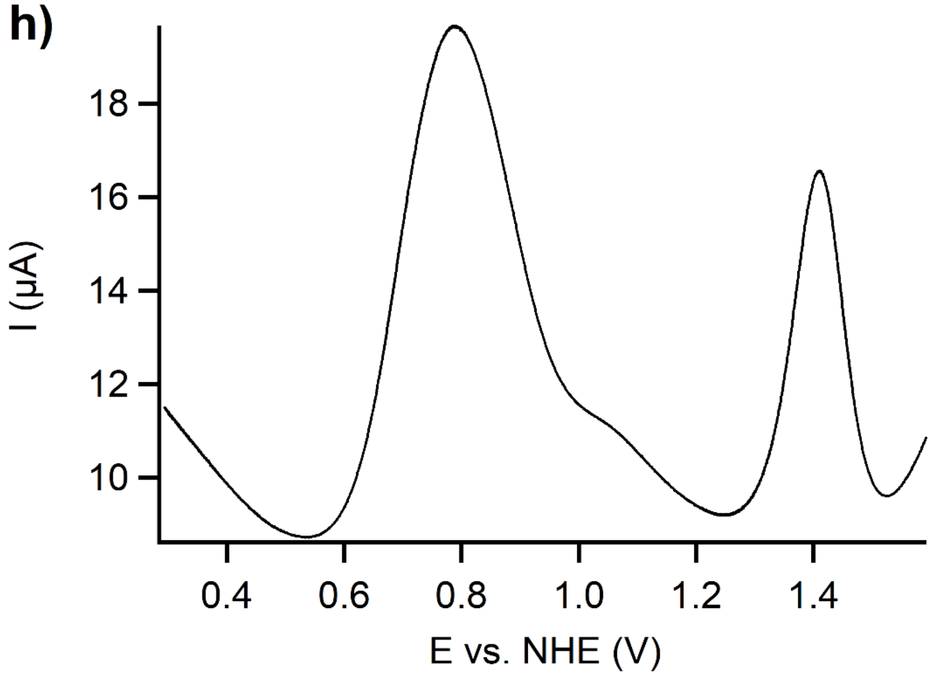
18
16
14
12
10
I (µA)
0.4
0.6
0.8
1.0
1.2
1.4
E vs. NHE (V)

g)

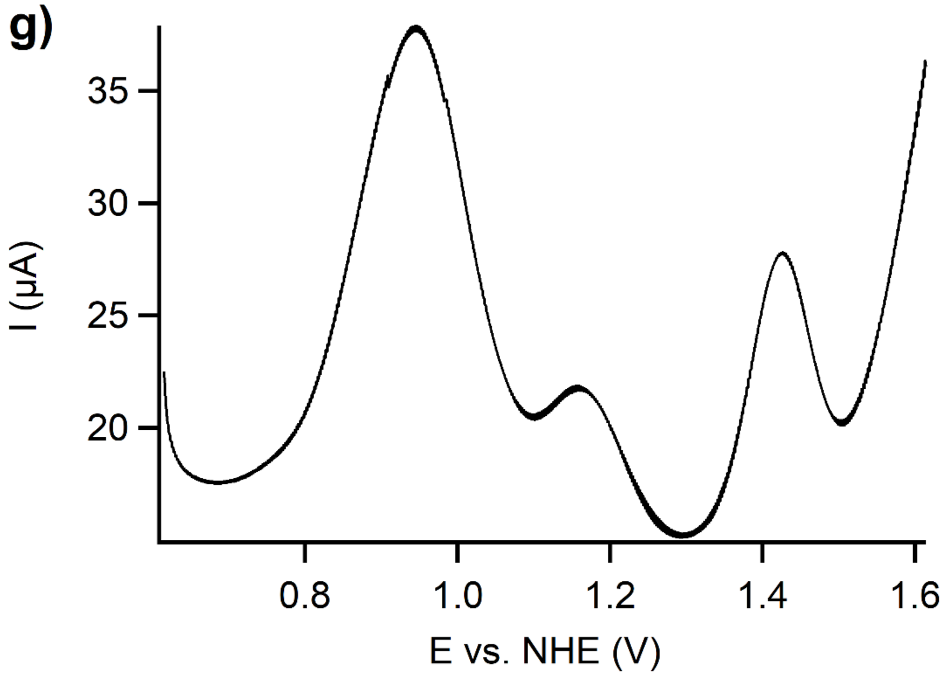
35
30
25
20
I (µA)
0.8
1.0
1.2
1.4
1.6
E vs. NHE (V)

i)

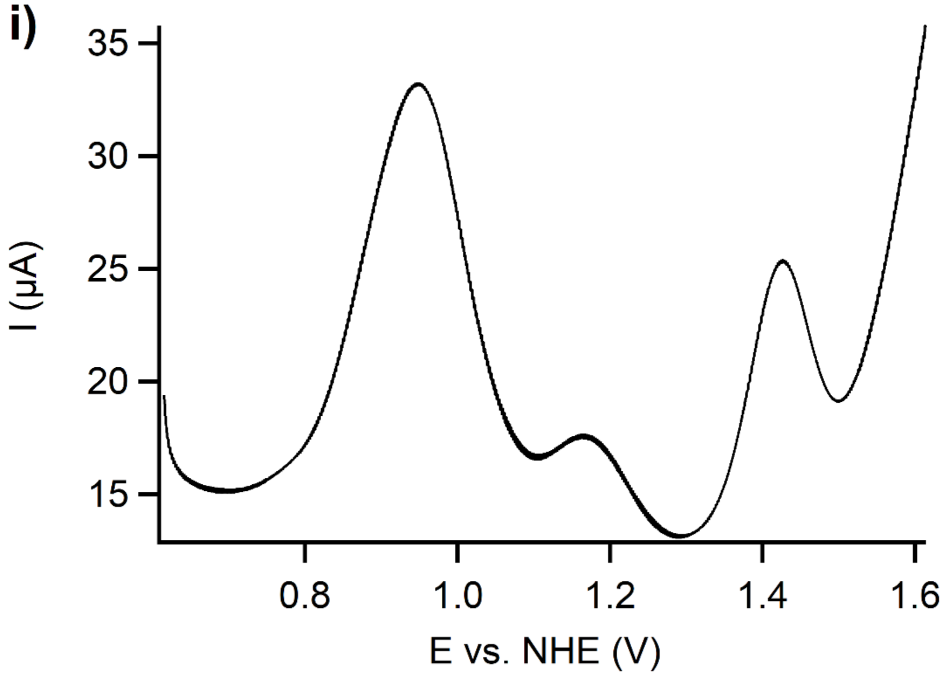
35
30
25
20
15
I (μA)
0.8
1.0
1.2
1.4
1.6
E vs. NHE (V)

j)

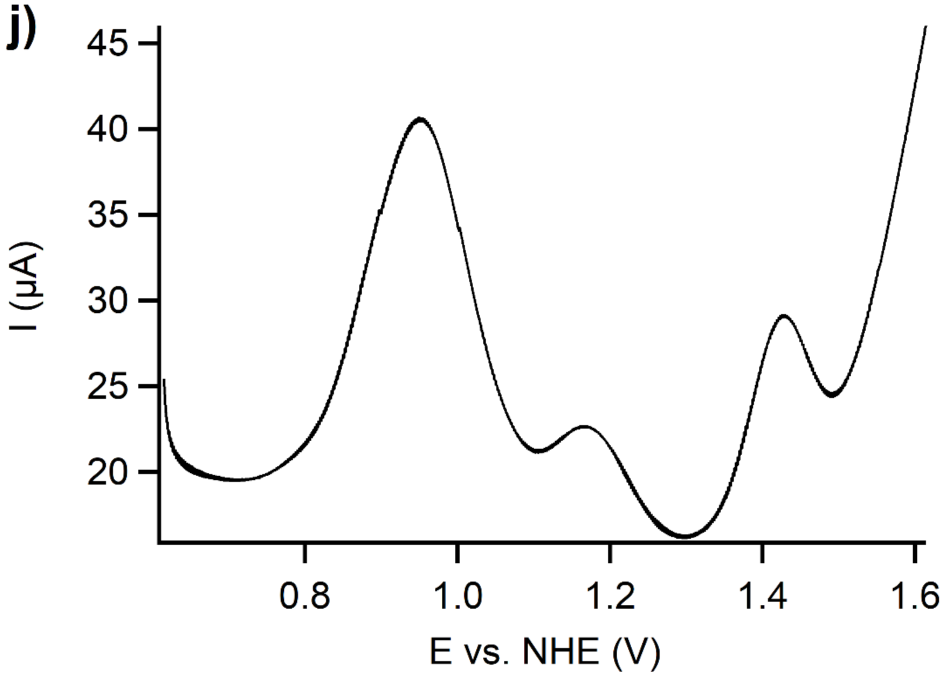
45
40
35
30
25
20
I (μA)
0.8
1.0
1.2
1.4
1.6
E vs. NHE (V)

k)

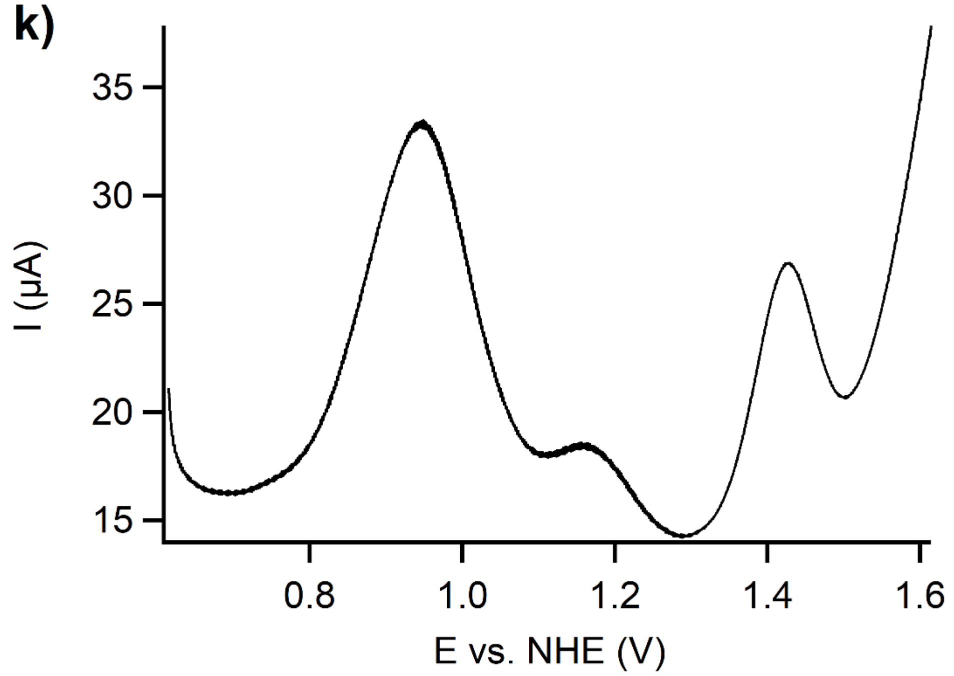
35
30
25
20
15
I (µA)
0.8
1.0
1.2
1.4
1.6
E vs. NHE (V)

l)

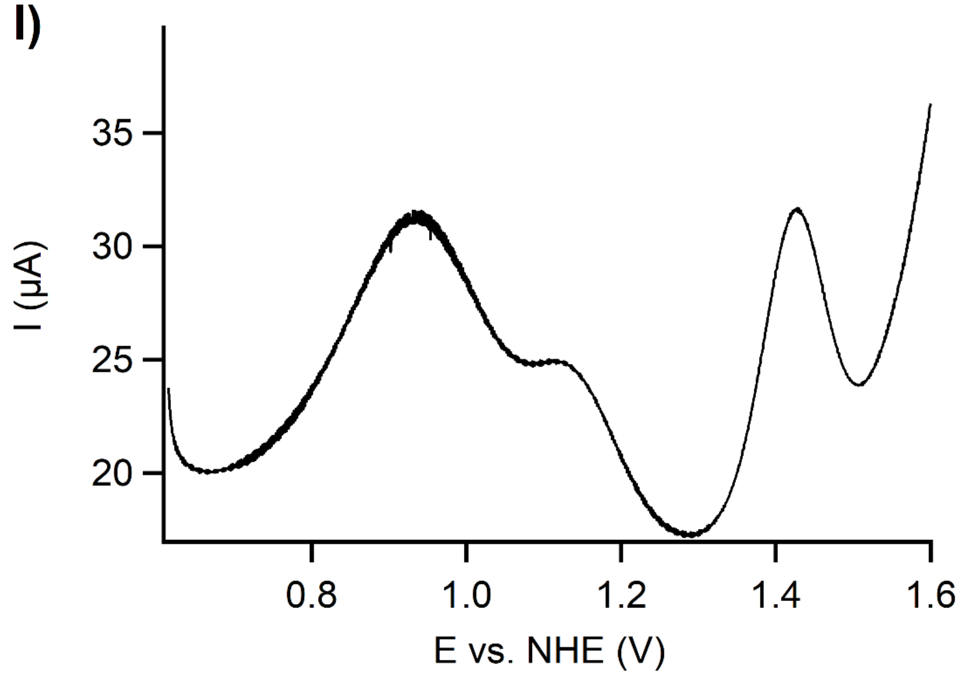
35
30
25
20
I (µA)
0.8
1.0
1.2
1.4
1.6
E vs. NHE (V)

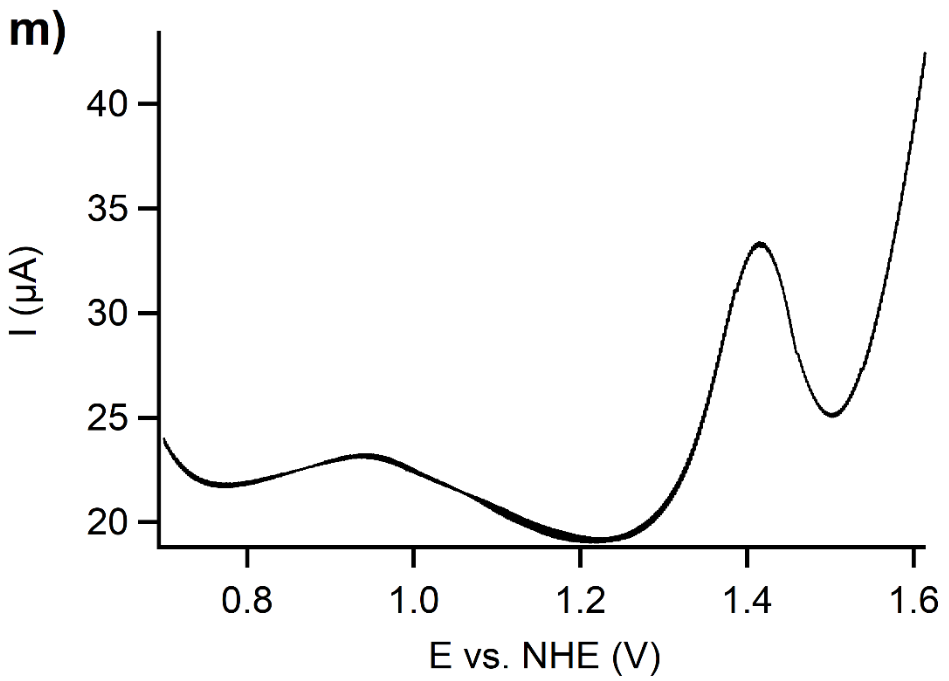

Figure S29. DPV data of *cis*-[Ru^{II}(4,4'-R_2-bpy)$_2$(H_2O)$_2$](OTf)$_2$ (**R=CF_3**) recorded in a 100 mM phosphate solution with the following pH values: a) 1.3 b) 2.539 c) 3.002 d) 3.780 e) 4.753 f) 5.383 g) 6.475 h) 6.900 i) 7.556 j) 7.967 k) 9.067 l) 10.043 m) 11.033. All figures were recorded at 0.5 mM catalyst concentration using a GC working electrode. An RHE reference electrode was used in a/h. In all other figures the reference electrode is Ag/AgCl. All potentials have been recalculated to NHE.

VII. References

(1) Durham, B.; Wilson, S. R.; Hodgson, D. J.; Meyer, T. J., Cis-trans photoisomerization in $Ru(bpy)_2(OH_2)_2^{2+}$. Crystal structure of trans-$[Ru(bpy)_2(OH_2)(OH)](ClO_4)_2$. *J. Am. Chem. Soc.* **1980,** *102*, 600-607.

(2) Ashford, D. L.; Brennaman, M. K.; Brown, R. J.; Keinan, S.; Concepcion, J. J.; Papanikolas, J. M.; Templeton, J. L.; Meyer, T. J., Varying the Electronic Structure of Surface-Bound Ruthenium(II) Polypyridyl Complexes. *Inorg. Chem.* **2015,** *54*, 460-469.

(3) Ito, A.; Kobayashi, N.; Teki, Y., Low-Energy and Long-Lived Emission from Polypyridyl Ruthenium(II) Complexes Having A Stable-Radical Substituent. *Inorg. Chem.* **2017,** *56*, 3794-3808.

(4) Nakagawa, A.; Ito, A.; Sakuda, E.; Fujii, S.; Kitamura, N., Emission Tuning of Heteroleptic Arylborane–Ruthenium(II) Complexes by Ancillary Ligands: Observation of Strickler–Berg-Type Relation. *Inorg. Chem.* **2018,** *57*, 9055-9066.

(5) Jackson, M. N.; Oh, S.; Kaminsky, C. J.; Chu, S. B.; Zhang, G.; Miller, J. T.; Surendranath, Y., Strong Electronic Coupling of Molecular Sites to Graphitic Electrodes via Pyrazine Conjugation. *J. Am. Chem. Soc.* **2018,** *140*, 1004-1010.

(6) Hue, R. J.; Vatassery, R.; Mann, K. R.; Gladfelter, W. L., Zinc oxide nanocrystal quenching of emission from electron-rich ruthenium-bipyridine complexes. *Dalton Trans.* **2015,** *44*, 4630-4639.

(7) Nußbaumer, W.; Gruber, H.; Greber, G. F., Wasserphotolyse mit Hilfe von funktionellen Tris-(2,2′-bipyridin)ruthenium(II)-Komplexen. *Monatsh. Chem.* **1988,** *119*, 1-15.

(8) Lennox, J. C.; Dempsey, J. L., Influence of Proton Acceptors on the Proton-Coupled Electron Transfer Reaction Kinetics of a Ruthenium–Tyrosine Complex. *J. Phys. Chem. B* **2017,** *121*, 10530-10542.

(9) Hoertz, P. G.; Staniszewski, A.; Marton, A.; Higgins, G. T.; Incarvito, C. D.; Rheingold, A. L.; Meyer, G. J., Toward Exceeding the Shockley−Queisser Limit: Photoinduced Interfacial Charge Transfer Processes that Store Energy in Excess of the Equilibrated Excited State. *J. Am. Chem. Soc.* **2006,** *128*, 8234-8245.

(10) Greaney, M. A.; Coyle, C. L.; Harmer, M. A.; Jordan, A.; Stiefel, E. I., Synthesis and characterization of mononuclear and dinuclear bis(2,2'-bipyridine)ruthenium(II) complexes containing sulfur-donor ligands. *Inorg. Chem.* **1989,** *28*, 912-920.

(11) Toyama, M.; Fujii, Y.; Endo, M., Bis-heteroleptic ruthenium(II) complex with 2-picolinamide: Synthesis, crystal structures, and spectroscopic study for anion recognition using the amide group. *Inorg. Chim. Acta* **2019,** *486*, 304-313.

(12) Qu, F.; Park, S.; Martinez, K.; Gray, J. L.; Thowfeik, F. S.; Lundeen, J. A.; Kuhn, A. E.; Charboneau, D. J.; Gerlach, D. L.; Lockart, M. M.; Law, J. A.; Jernigan, K. L.; Chambers, N.; Zeller, M.; Piro, N. A.; Kassel, W. S.; Schmehl, R. H.; Paul, J. J.; Merino, E. J.; Kim, Y.; Papish, E. T., Ruthenium Complexes are pH-Activated Metallo Prodrugs (pHAMPs) with Light-Triggered Selective Toxicity Toward Cancer Cells. *Inorg. Chem.* **2017,** *56*, 7519-7532.

(13) Dobson, J. C.; Meyer, T. J., Redox properties and ligand loss chemistry in aqua/hydroxo/oxo complexes derived from cis- and trans-[(bpy)2RuII(OH2)2]2+. *Inorg. Chem.* **1988,** *27*, 3283-3291.

(14) Bard, A. J.; Faulkner, L. R., *Electrochemical Methods: Fundamentals and Applications, 2nd Edition*. John Wiley & Sons, Incorporated: 2000.

(15) Costentin, C.; Drouet, S.; Robert, M.; Savéant, J.-M., Turnover Numbers, Turnover Frequencies, and Overpotential in Molecular Catalysis of Electrochemical Reactions. Cyclic Voltammetry and Preparative-Scale Electrolysis. *J. Am. Chem. Soc.* **2012,** *134*, 11235-11242.

(16) Elgrishi, N.; Rountree, K. J.; McCarthy, B. D.; Rountree, E. S.; Eisenhart, T. T.; Dempsey, J. L., A Practical Beginner’s Guide to Cyclic Voltammetry. *J. Chem. Educ.* **2018,** *95*, 197-206.
